# Genome‐Wide by Lifetime Environment Interaction Studies of Brain Imaging Phenotypes

**DOI:** 10.1002/advs.202500852

**Published:** 2025-11-29

**Authors:** Sijia Wang, Meiyun Wang, Peng Zhang, Jingliang Cheng, Longjiang Zhang, Wenzhen Zhu, Shijun Qiu, Zuojun Geng, Guangbin Cui, Yongqiang Yu, Weihua Liao, Xi‐Nian Zuo, Hui Zhang, Bo Gao, Xiaojun Xu, Tong Han, Zhenwei Yao, Quan Zhang, Feng Liu, Qiang Xu, Jiayuan Xu, Jilian Fu, Nana Liu, Yuan Ji, Jie Tang, Lining Guo, Mengge Liu, Xiaoxiao Xiao, Xiaoxuan Liu, Wei Li, Caihong Wang, Wei Wei, Dapeng Shi, Su Lui, Zhihan Yan, Feng Chen, Jing Zhang, Wen Shen, Yanwei Miao, Dawei Wang, Jia‐Hong Gao, Yunjun Yang, Kai Xu, Junfang Xian, Bing Zhang, Xiaochu Zhang, Zhaoxiang Ye, Le Yu, Wen Qin, Meng Liang, Chunshui Yu, the CHIMGEN Consortium

**Affiliations:** ^1^ Department of Radiology Tianjin Key Lab of Functional Imaging and State Key Laboratory of Experimental Hematology Tianjin Medical University General Hospital Tianjin 300052 China; ^2^ Department of Radiology Henan Provincial People's Hospital & Zhengzhou University People's Hospital Zhengzhou 450003 China; ^3^ Biomedical Institute Henan Academy of Sciences Zhengzhou 450199 China; ^4^ Department of Radiology Tianjin Medical University Cancer Institute & Hospital National Clinical Research Center for Cancer Tianjin's Clinical Research Center for Cancer Tianjin Medical University Ministry of Education Key Laboratory of Cancer Prevention and Therapy Tianjin 300060 China; ^5^ Department of Magnetic Resonance Imaging The First Affiliated Hospital of Zhengzhou University Zhengzhou 450052 China; ^6^ Department of Radiology Jinling Hospital, Affiliated Hospital of Medical School Nanjing University Nanjing 210002 China; ^7^ Department of Radiology Tongji Hospital Tongji Medical College Huazhong University of Science and Technology Wuhan 430030 China; ^8^ Department of Medical Imaging The First Affiliated Hospital of Guangzhou University of Traditional Chinese Medicine Guangzhou 510405 China; ^9^ Department of Medical Imaging The Second Hospital of Hebei Medical University Shijiazhuang 050000 China; ^10^ Functional and Molecular Imaging Key Lab of Shaanxi Province & Department of Radiology Tangdu Hospital Air Force Medical University Xi'an 710038 China; ^11^ Department of Radiology The First Affiliated Hospital of Anhui Medical University Hefei 230022 China; ^12^ Department of Radiology Xiangya Hospital Central South University Changsha 410008 China; ^13^ Molecular Imaging Research Center of Central South University Changsha 410008 China; ^14^ National Clinical Research Center for Geriatric Disorders Xiangya Hospital Central South University Changsha 410008 China; ^15^ Developmental Population Neuroscience Research Center at IDG/McGovern Institute for Brain Research Beijing Normal University Beijing 100875 China; ^16^ Institute of Psychology Chinese Academy of Sciences Beijing 100101 China; ^17^ Department of Radiology The First Hospital of Shanxi Medical University Taiyuan 030001 China; ^18^ Department of Radiology The Affiliated Hospital of Guizhou Medical University Guiyang 550004 China; ^19^ Department of Radiology Yantai Yuhuangding Hospital Yantai 264000 China; ^20^ Department of Radiology The Second Affiliated Hospital of Zhejiang University School of Medicine Hangzhou 310009 China; ^21^ Department of Radiology Tianjin Huanhu Hospital Tianjin 300350 China; ^22^ Department of Radiology Huashan Hospital Fudan University Shanghai 200040 China; ^23^ Department of Radiology Characteristic Medical Center of Chinese People's Armed Police Force Tianjin 300162 China; ^24^ School of Medical Imaging (School of Medical Technology) and Tianjin Key Laboratory of Functional Imaging Tianjin Medical University Tianjin 300203 China; ^25^ Ministry of Education Key Laboratory for Earth System Modeling Department of Earth System Science Tsinghua University Beijing 100084 China; ^26^ Department of Radiology Huaxi MR Research Center (HMRRC) Functional and Molecular Imaging key Laboratory of Sichuan Province West China Hospital of Sichuan University Chengdu 610041 China; ^27^ Research Unit of Psychoradiology Chinese Academy of Medical Sciences Chengdu 610041 China; ^28^ Department of Radiology The Second Affiliated Hospital and Yuying Children's Hospital of Wenzhou Medical University Wenzhou 325027 China; ^29^ Department of Radiology Hainan General Hospital (Hainan Affiliated Hospital of Hainan Medical University) Haikou 570311 China; ^30^ Department of Magnetic Resonance Lanzhou University Second Hospital Lanzhou 730030 China; ^31^ Gansu Province Clinical Research Center for Functional and Molecular Imaging Lanzhou 730030 China; ^32^ Department of Radiology Tianjin First Center Hospital Tianjin 300192 China; ^33^ Department of Radiology The First Affiliated Hospital of Dalian Medical University Dalian 116011 China; ^34^ Department of Radiology Qilu Hospital of Shandong University Jinan 250012 China; ^35^ Center for MRI Research Academy for Advanced Interdisciplinary Studies Peking University Beijing 100871 China; ^36^ Department of Radiology The First Affiliated Hospital of Wenzhou Medical University Wenzhou 325000 China; ^37^ Department of Radiology The Affiliated Hospital of Xuzhou Medical University Xuzhou 221006 China; ^38^ Department of Radiology Beijing Tongren Hospital Capital Medical University Beijing 100730 China; ^39^ Department of Radiology Drum Tower Hospital Medical School of Nanjing University Nanjing 210008 China; ^40^ Division of Life Science and Medicine University of Science & Technology of China Hefei 230027 China

**Keywords:** brain imaging phenotypes, gene‐environment interactions, main effects, sensitive periods

## Abstract

Brain structure and function show substantial individual differences, finely controlled by genes, environments, and their interactions. Despite the increasing knowledge about genetic and environmental main effects, gene‐environment interaction effects on brain phenotypes remain elusive. This study investigates genome‐wide by environment (41 exposures) interactions on 598 brain imaging phenotypes in 7084 healthy young adults. Both univariate and multivariate analyses identify 486 significant gene‐environment interactions, scattered across the genome, exposome, and phenome. These interactions explain more variances of phenotypes than genetic and environmental main effects (100% of genetic and 96% of environmental main effects are non‐significant). Variants with interactions are enriched in intronic and intergenic regions, comprising 79 regulatory variants and 145 associated with brain gene expression. Protein‐protein interaction network analyses reveal distinct interaction networks for genes associated with air pollution (hubs: *H4C6*, *SMARCA4*, and *RPS11*) and urbanicity (hubs: *CCND1*, *CALM3*, and *CDK2*) exposures. Genes that interacted with air pollution exposures exhibit enrichment in pathways related to metal ion detoxification and homeostasis. For time‐varying exposures, 144 interactions demonstrate sensitive periods, predominantly in childhood (ages 4–7) and adolescence (ages 12–15). These findings highlight the value of genome‐wide by exposome‐wide interaction studies, which may offer crucial information for optimizing brain health outcomes.

## Graphical abstract

This study explores genome‐wide by lifetime environment interactions on brain imaging phenotypes. Gene‐environment interactions explain more phenotypic variance than main effects, pinpoint regulatory variants, and reveal exposure‐specific biological pathways. Critical sensitive periods are identified in childhood and adolescence, offering novel insights for optimizing brain health.

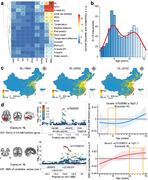

## 1 Introduction

Brain structure and function vary greatly between individuals, which can be measured by various non‐invasive brain magnetic resonance imaging (MRI) techniques. Normal variations in brain structure and function may account for the diversity of behavioral performance, such as individual differences in cognition and emotion.^[^ ^1^, ^2^, ^3^, ^4^, ^5^ ^]^ Abnormal brain structural and functional alterations are observed in neuropsychiatric disorders, such as the Alzheimer's disease,^[^ ^6^ ^]^ multiple sclerosis,^[^ ^7^ ^]^ schizophrenia,^[^ ^8^ ^]^ and depression.^[^ ^9^ ^]^ As both normal variations and abnormal alterations in brain structure and function are finely controlled by genes, environments, and their interactions,^[^ ^10^, ^11^, ^12^, ^13^ ^]^ MRI may provide enormous intermediate endophenotypes for clarifying how genetic and environmental factors influence human behavioral performance and neuropsychiatric disorders.

Brain MRI can provide abundant imaging‐derived phenotypes (IDPs) to evaluate different aspects of brain structure and function. For example, IDPs from structural MRI can measure the macrostructural properties, such as gray matter volume (GMV),^[^ ^14^ ^]^ cortical thickness (CT), and surface area (SA) of brain regions.^[^ ^15^ ^]^ IDPs from diffusion MRI can evaluate the microstructural integrity of white matter tracts using fractional anisotropy (FA).^[^ ^16^ ^]^ IDPs from resting‐state functional MRI (fMRI) can estimate the spontaneous neuronal activity within and temporal synchronization of fMRI signals between resting‐state networks (RSNs)^[^ ^17^ ^]^ using functional activity amplitude (Amp)^[^ ^17^ ^]^ and functional connectivity (FC),^[^ ^17^ ^]^ respectively. Additionally, the spontaneous neuronal activity of each gray matter voxel can be assessed by regional homogeneity (ReHo) of low‐frequency fMRI signal fluctuations.^[^ ^18^ ^]^

In the past decades, numerous studies have investigated the influence of genetic variants and environmental exposures on brain imaging phenotypes. As for the genetic effects, many genome‐wide association studies (GWASs) have identified thousands of genetic loci associated with brain IDPs^[^ ^10^, ^17^, ^19^, ^20^, ^21^, ^22^ ^]^ In terms of the environmental effects, brain IDPs have been associated with environmental exposures, such as air pollution,^[^ ^23^ ^]^ urbanicity,^[^ ^24^ ^]^ green space,^[^ ^25^ ^]^ socioeconomic status,^[^ ^26^ ^]^ childhood trauma,^[^ ^27^ ^]^ and lifestyle.^[^ ^28^ ^]^ In agreement with the temporally dynamic nature of exposome (totality of environmental exposures from the prenatal period onwards^[^ ^13^ ^]^), several studies have investigated the sensitive periods for environmental effects on brain IDPs. For instance, the urbanicity score during childhood shows the largest effect on cerebellar volume.^[^ ^24^ ^]^

In addition to independent genetic and environmental effects, gene‐environment (G × E) interactions may also contribute to the individual differences in brain IDPs, as some exposures can alter the genetic effects on brain IDPs via epigenetic mechanisms such as DNA methylation^[^ ^29^ ^]^ and histone modification.^[^ ^30^ ^]^ It is important to identify G × E interactions because they may inform precise interventions by identifying individuals with specific genotypes that are especially sensitive to a given exposure. The unbiased way to identify G × E interactions is by searching for them throughout the genome and exposome; however, such kind of studies are still lacking so far due to the challenge of simultaneously collecting the genome, exposome, and phenome data in a large sample of participants. The dilemma is also applied to G × E interaction studies on brain IDPs.^[^ ^31^, ^32^, ^33^ ^]^ Furthermore, G × E interactions may have sensitive periods similar to environmental effects, whereas studies on the sensitive periods of G × E interactions on brain IDPs are lacking because such studies additionally require the lifetime exposure data.

**Figure 1:**
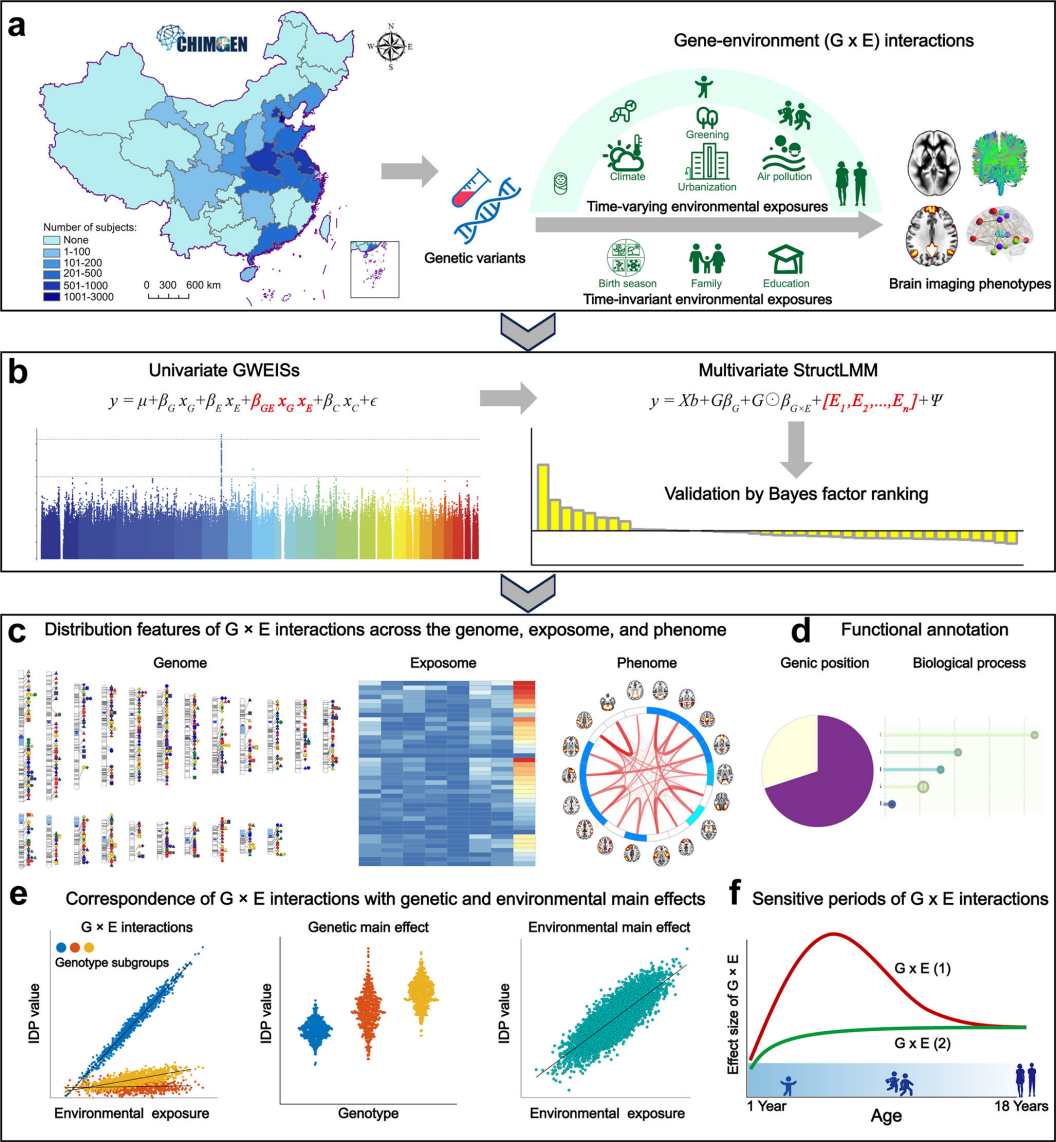
A schematic summary of the study design. **a)** We investigate G × E interactions between 54 61 901 autosomal and 14 7631 X‐chromosomal variants, and 41 exposures (21 time‐varying and 20 time‐invariant exposures) on 598 brain IDPs (seven categories from three MRI modalities) in 7306 participants from the CHIMGEN study. **b)** We identify reliable G × E interactions with three consecutive steps: discovering all genome‐wide significant G × E interactions and variant‐IDP pairs using the univariate GWEISs; confirming the variant‐IDP pairs with the score test in the multivariate structured linear mixed model (StructLMM); and verifying G × E interactions according to the Bayes factor ranking in the multivariate model. **c)** Investigating distribution features of G × E interactions across the genome, exposome, and phenome. **d)** Functional annotation and enrichment of genetic variants and genes with significant G × E interactions. **e)** Investigating the correspondence of G × E interactions with genetic and environmental main effects. **f)** Identifying the sensitive periods of G × E interactions on brain IDPs for the 21 time‐varying exposures. Abbreviations: GWEISs, genome‐wide by environment interaction studies; IDP, imaging‐derived phenotype.

The Chinese Imaging Genetics (CHIMGEN) study^[^ ^34^ ^]^ collected the environmental, genomic, and brain MRI data from 7306 Chinese Han participants aged 18‐30 years. Considering for the various categories of exposures and the large number of exposures with lifetime assessment, the CHIMGEN cohort may provide a unique opportunity for identifying the genome‐wide by lifetime environment interactions on brain IDPs and their sensitive periods. Here, we conducted genome‐wide by environment interaction studies (GWEISs) between 54 61 901 autosomal and 147631 X‐chromosomal variants with an imputation information score (INFO) ≥ 0.9 and minor allele frequency (MAF) ≥ 0.05, and 41 exposures on 598 brain IDPs to unbiasedly identify G × E interactions on brain IDPs and their sensitive periods. We also conducted functional annotations for genetic variants with significant interactions and investigated the correspondence of G × E interactions with genetic and environmental main effects. The study design is shown in **Figure** **1**.

## 2 Results

### 2.1 Participants, data, and scheme for G × E interaction studies

Among the 7306 CHIMGEN participants, we excluded 143 without qualified genomic data, 71 without qualified data for any MRI modalities, and eight without qualified data for any exposure variables, and finally included 7084 participants (Figure S1, Supporting Information). The details about the acquisition, preprocessing, and quality control of the genetic, environmental, and MRI data are described in **Experimental Section** and **Supporting Information**. Briefly, we extracted 598 brain IDPs from the structural, diffusion, and resting‐state fMRI data, including 127 GMV‐IDPs, 64 CT‐IDPs, 64 SA‐IDPs, 48 FA‐IDPs, 18 Amp‐IDPs, 153 FC‐IDPs, and 124 ReHo‐IDPs (Table S1, Supporting Information). We included 5461901 imputed autosomal variants and 14 7631 X‐chromosomal variants with INFO ≥ 0.9 and MAF ≥ 0.05 to ensure the high quality of genetic data imputation and the acceptable sample size in the smallest genotype subgroup for each G × E interaction analysis. We included 21 time‐varying exposures, mainly from satellite remote sensing data, and 20 time‐invariant exposures, primarily from paper‐based questionnaires (Table S2, Supporting Information). For time‐varying exposures, we extracted the average values of annual exposures for the first 18 years of age as environmental variables, including the population count, normalized difference built‐up index (NDBI), normalized difference vegetation index (NDVI), nighttime lights (NL), particular matter (PM_2.5_), nitrogen dioxide (NO_2_), Palmer drought severity index (PDSI), precipitation, humidity, surface pressure, wind speed, temperature, temperature difference (TD), bare‐land%, cropland%, grassland%, forest%, scrub‐land%, built‐up%, water body%, and land use intensity (LUI). The time‐invariant exposures included the altitude, latitude, and longitude of the birthplace, urbanicity score, four birth seasons, education years, mother's and father's ages at birth, only child status, parental divorce and death, and sum score and five subscale scores of childhood trauma questionnaire (CTQ). Based on the Spearman correlations among these environmental exposures, we conducted hierarchical clustering to categorize them into four distinct exposure categories (Figure S2, Supporting Information): air pollution (nine exposures: PM_2.5,_ NO_2_, wind speed, surface pressure, grassland%, scrub‐land%, forest%, and birthplace altitude and longitude), climate (seven exposures: temperature, TD, precipitation, humidity, PDSI, NDBI, and birthplace latitude), urbanicity (eight exposures: NDVI, LUI, built‐up%, NL, population count, cropland%, urbanicity score, and only‐child status), and family factors (17 exposures: spring born, summer born, autumn born, winter born, parental divorce, parental death, father's age at birth, mother's age at birth, emotional abuse, physical abuse, sexual abuse, emotional neglect, physical neglect, and total score of CTQ, education years, bare‐land%, and water body%).

We combined univariate and multivariate analyses to identify G × E interactions on brain IDPs. First, we conducted univariate GWEIS (inflation‐adjusted *P* < 5 × 10^−8^, without additionally correcting for the numbers of exposures and brain IDPs) to screen variant‐exposure pairs on each brain IDP, while controlling for age, sex, and the first 10 genetic principal components (PCs) for all IDPs, and additionally controlling for the total intracranial volume (TIV) for GMV‐IDPs, total SA for SA‐IDPs, mean CT for CT‐IDPs, and mean framewise displacement (FD) for all IDPs from fMRI data. Using an inflation‐adjusted genome‐wide significant threshold (*P* < 5 × 10^−8^), we identified the significant G × E interactions and variant‐IDP pairs. Then, for each screened significant variant‐IDP pair, a structured linear mixed model (StructLMM) was further applied to validate the replicability of this variant in a multivariate association condition,^[^ ^35^ ^]^ which included all exposures in the same model to estimate the overall interaction between the variant and all exposures on the IDP using a score test. We defined a variant‐IDP pair to be verified if the Benjamini‐Hochberg false discovery rate (BH‐FDR) *q* < 0.05 in the score test. The model can also output the Bayes factor (BF) of each exposure to represent the evidence for the exposure in driving the overall G × E interaction for each variant‐IDP pair. We defined a G × E interaction to be verified if the BF value of the exposure was ranked no larger than the number of significant interactions identified for the variant‐IDP pair in the univariate GWEISs. As the G × E interaction analyses require participants with complete genomic, environmental, and brain imaging data, we applied the principle of maximizing sample size to include as many participants as possible in the univariate GWEISs (*n* = 5066–7039; Table S3, Supporting Information). As the multivariate interaction analyses further require the participants with complete data for all exposures, we only included 5558 participants for structural, 5547 for diffusion, and 5006 for functional IDPs (Figure S1 and Table S3, Supporting Information).

### 2.2 Univariate G × E interactions on Brain IDPs

In 5066–7039 CHIMGEN participants, we conducted 24 518 univariate GWEISs for 41 environmental exposures and 598 brain IDPs across 54 61 901 autosomal variants using PLINK2.^[^ ^36^ ^]^ We found genome‐wide significant G × E interactions (*P* < 5 × 10^−8^) in 1121 GWEISs. As some GWEISs showed relatively large genomic control inflation factors^[^ ^37^ ^]^ (Figure S3, Supporting Information), we further applied the genomic control correction to the 1121 GWEIS summary statistics, from which we still identified 744 GWEISs with significant interactions (inflation‐adjusted *P* < 5 × 10^−8^), including 759 linkage disequilibrium (LD)‐independent G × E interactions, 703 independent G × E interactions (conditional analyses accounting for highly correlated exposures), and 703 LD‐independent variant‐IDP pairs (Table S4, Supporting Information). We further identified G × E interactions (*P*  <  5 × 10^−8^) between 147631 X‐chromosomal variants and 41 exposures on the 598 brain IDPs. We found 50 independent G × E interactions (accounting for both LD and correlated exposures), involving 49 unique variants, 43 LD‐independent variants, 28 LD‐independent loci, 28 exposures, and 45 brain IDPs (Table S5, Supporting Information). Considering the lack of putative approaches for X‐chromosomal analyses, all subsequent analyses were focused on the interactions derived from autosomal variants.

### 2.3 Multivariate G × E Interactions on Brain IDPs

**Figure 2:**
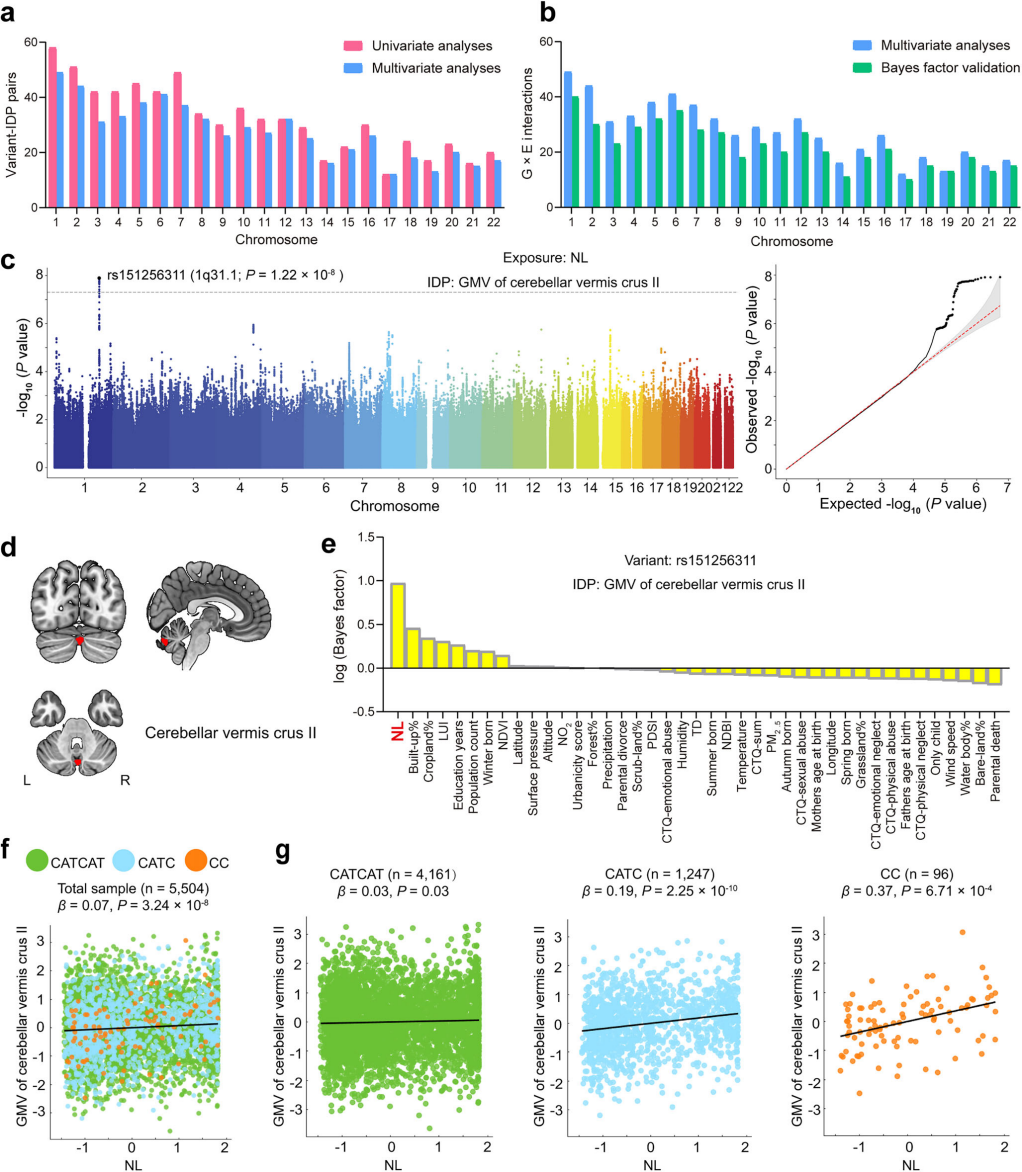
Genome‐wide significant G × E interactions on brain IDPs. **a)** The histogram shows the distribution of significant variant‐IDP interaction pairs across the autosomes. The pink bars represent the 703 significant variant‐IDP interaction pairs discovered in univariate G × E interaction analyses (inflation‐adjusted *P* < 5 × 10^−8^), while blue bars show the 602 significant variant‐IDP interaction pairs further validated in multivariate G × E interaction analyses (*q_c_* < 0.05, FDR corrected). **b)** The histogram shows the distribution of significant G × E interactions across the 22 autosomes. The blue bars represent the 602 significant G × E interactions identified by univariate analyses (inflation‐adjusted *P* < 5 × 10^−8^) and further included in variant‐IDP interaction pairs confirmed by multivariate analyses (*q_c_* < 0.05, FDR corrected). The green bars represent the 486 significant G × E interactions further verified by the rank of Bayes factors. **c–g)** An example of significant G × E interactions on brain IDPs. **c)** The Manhattan and quantile‐quantile plots show the interactions between genomic variants and NL on the GMV of cerebellar vermis crus II. The lead variant rs151256311 with significant interaction (*P* = 1.22 × 10^−8^) is marked in the Manhattan plot, in which the gray line indicates *P* = 5 × 10^−8^. **d**) Visualization of the cerebellar vermis crus II on the 3D brain structural images. **e,** NL shows the largest Bayes factor among the 41 exposures in the multivariate model for the variant‐IDP pair between rs151256311 and GMV of cerebellar vermis crus II. **f)** The scatter plot shows the overall correlation (*β* = 0.07, *P* = 3.24 × 10^−8^) between NL and GMV of cerebellar vermis crus II in the total sample (*n* = 5504). **g)** The scatter plots show the same correlation in the three genotype subgroups of rs151256311. The correlation is only significant in the CC (*β* = 0.37, *P* = 6.71 × 10^−4^, *n* = 96) and CATC (*β* = 0.19, *P* = 2.25 × 10^−10^, *n* = 1247) subgroups at *P* < 0.017 (Bonferroni‐adjusted for the three subgroups). Abbreviations: CTQ, childhood trauma questionnaire; FDR, false discovery rate; GMV, gray matter volume; IDP, imaging‐derived phenotype; L, left; LD, linkage disequilibrium; LUI, land use intensity; NDBI, normalized difference built‐up index; NDVI, normalized difference vegetation index; NL, night‐time light; NO_2_, nitrogen dioxide; PDSI, Palmer drought severity index; PM, particular matter; R, right; TD, temperature difference.

We applied the multivariate StructLMM to each of the 703 LD‐independent variant‐IDP pairs identified in the univariate GWEISs, and verified 602 (85.63%) variant‐IDP pairs (**Figure** **2**a; Table S6, Supporting Information) at *q* < 0.05 (BH‐FDR corrected) in the score test, including 602 independent G × E interactions (Table S7, Supporting Information) identified in the univariate GWEISs. By further considering the BF ranks of exposures in each variant‐IDP pair (Table S6, Supporting Information), we still confirmed 486 LD‐independent G × E interactions (Figure 2b), involving 484 unique variants, 474 LD‐independent variants, 401 LD‐independent loci, 39 exposures, and 335 brain IDPs (Table S7, Supporting Information). We found that interaction analyses provided information that could not be identified in the analyses of genetic and environmental main effects. For example, we found a significant interaction (GWEIS: *P* = 1.22 × 10^−8^; score test: *q* = 4.67 × 10^−5^; BF rank: NL = 1) between rs151256311 at 1q31.1 and NL on the GMV of cerebellar vermis crus II (Figure 2c–e). Consistent with a previous study,^[^ ^24^ ^]^ we found a positive correlation (*β* = 0.07, *P* = 3.24 × 10^−8^) between NL and cerebellar volume (Figure 2f). However, in the analyses of genotype subgroups, we found that this positive correlation was significant (*P* < 0.017, adjusted for the three subgroups) only in CATC (*β* = 0.19, *P* = 2.25 × 10^−10^, *n* = 1,247) and CC (*β* = 0.37, *P* = 6.71 × 10^−4^, *n* = 96) subgroups rather than in CATCAT (*β* = 0.03, *P* = 0.03, *n* = 4,161) subgroup (Figure 2g).

### 2.4 Sensitivity Analyses

In this study, ComBat harmonization was applied to brain IDPs to adjust for the scanner effect, although the inclusion of the scanner as a covariate is a commonly used method. To assess whether our findings are influenced by adjustment methods for the scanner effect, we recalculated the 468 independent G × E interactions by including the scanner as a covariate and identified a strong Spearman correlation (*rho* = 0.99, *P* < 1.00 × 10^−322^; Figure S4, Supporting Information) of the estimated effects (beta values) derived from the two methods. This result indicates that the identified interactions are not influenced by adjustment methods for the scanner effect.

Although conditional analyses were applied to account for correlated exposures in identifying independent interactions, we performed two sensitivity analyses on the 486 independent G × E interactions. In the first analysis, we included all other 40 exposures as covariates in the interaction model, and found that all 486 interactions remained significant after BH‐FDR correction (*q* < 0.05), with 268 also retaining genome‐wide significance (*P* < 5 × 10^−8^). In the second analysis, we further extended this model by additionally including all other 40 exposure‐by‐gene interaction terms. We found that 281 interactions remained significant after BH‐FDR correction (*q* < 0.05) (Table S8, Supporting Information), although only nine passed the stringent genome‐wide significance threshold, which is likely due to small sample size and increased model complexity.^[^ ^38^ ^]^

To evaluate whether sex influences the interpretations of G × E interactions, we conducted sex‐stratified analyses for the 486 G × E interactions by recalculating these interactions in males and females, respectively. All interactions showed a consistent direction of effects (the same effect allele and the same sign of beta values) across the three analyses (male‐specific, female‐specific, and sex‐combined analyses) (Table S9, Supporting Information). The effect sizes (beta values) of interactions derived from the sex‐combined analysis were highly correlated with those obtained from both male‐specific (*rho* = 0.93, *P* = 2.03 × 10^−^^207^) and female‐specific (*rho* = 0.97, *P* = 3.83 × 10^−306^) analyses. Furthermore, 464 (95.47%) interactions were significant (*q* < 0.05, BH‐FDR corrected) in males, while all remained significant (*q* < 0.05, BH‐FDR corrected) in females. These results suggest that sex has little influence on the observed G × E interactions.

### 2.5 Genetic Variants With G × E Interactions

**Figure 3:**
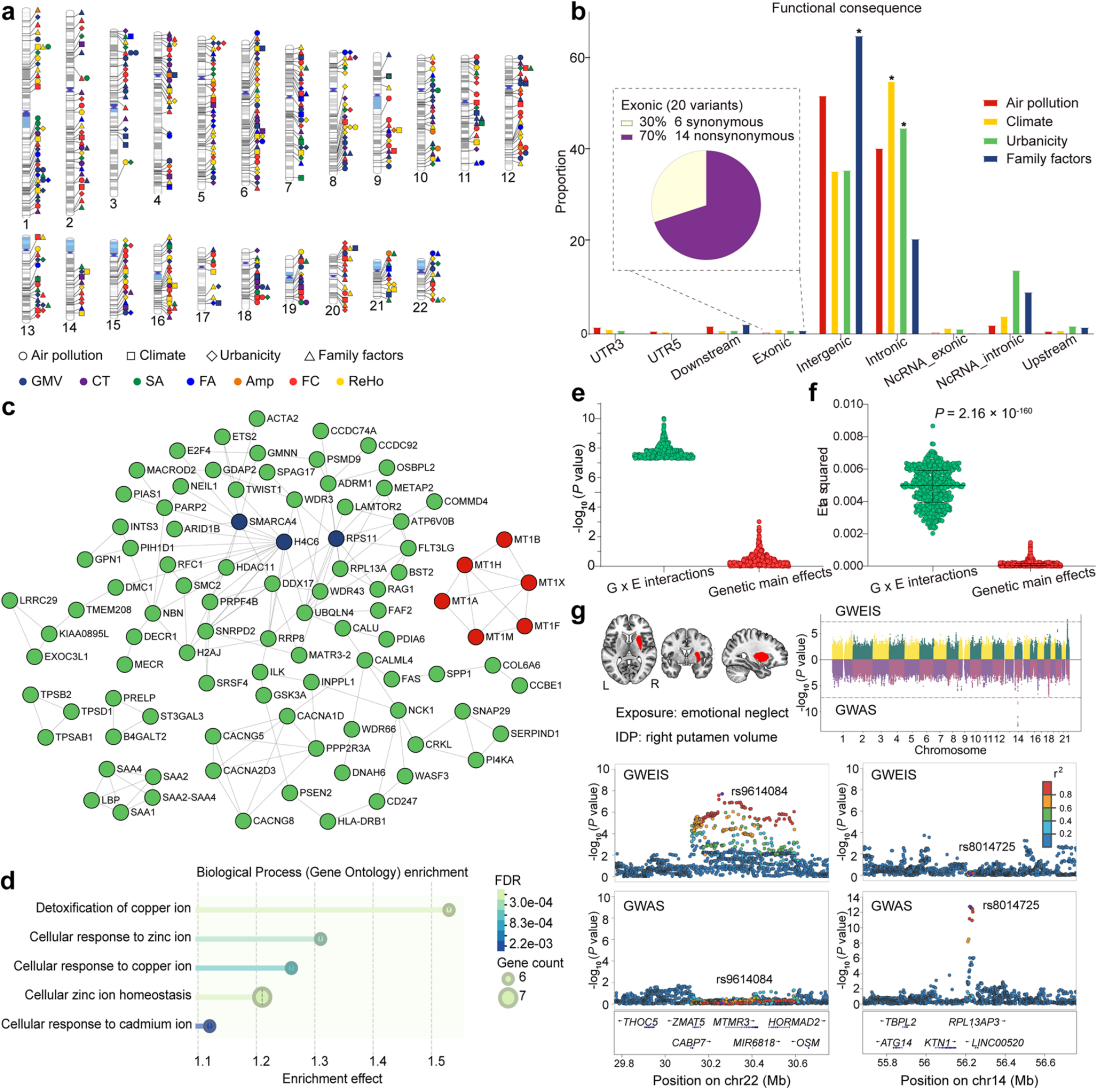
Genetic variants with significant G × E interactions on brain IDPs. The 486 verified G × E interactions involve 401 LD‐independent loci, including 461, 387, 621, and 1142 unique variants interacting with air pollution, climate, urbanicity, and family exposures, respectively (inflation‐adjusted *P* < 5 × 10^−8^ in the univariate GWEISs). **a)** The ideogram shows the genomic location of the 401 LD‐independent loci stratified by the MRI metrics (colors) and exposure categories (shapes). **b)** Categorizing variants interacting with each exposure category based on the genomic location and functional consequence. Color indicates exposure categories, while asterisks denote significance (*P* < 0.05/9/4 = 0.0014, Bonferroni correction for nine consequences and four exposure categories). **c)** PPI network for genes interacting with air pollution on brain IDPs. The network displays genes with the top 100 highest degrees of connectivity. The top three hub genes are highlighted with blue dots. Genes implicated in metal ion detoxification and homeostasis pathways are marked with red dots. **d)** The bubble plot shows the gene ontology terms of biological processes (*q_c_* < 0.05, FDR corrected) enriched by protein‐coding genes interacting with air pollution. The *x*‐axis shows the enrichment effect of each term on the *y*‐axis. The bubble size and color represent the number of genes enriched for each term and the significance of each term in the over‐representation analysis, respectively. **e, f,** The correspondence in significance **e**) and IDP explained variance **f**) between 486 independent G × E interactions (green) and 486 genetic main effects (red) in the verified variant‐IDP pairs. No genetic main effects survive at *P* < 5 × 10^−8^ **e**). The Wilcoxon sum rank test indicates that G × E interactions explain much larger proportions of IDP variances (*Z* = 26.99, *P* = 2.16 × 10^−160^) than genetic main effects **f**). The three lines from bottom to top in violin plots indicate the first quartile, median, and third quartile. **g)** An example of inconsistency between G × E interactions and genetic main effects in the exposure‐IDP pair between CTQ‐emotional neglect and right putamen volume (upper left). To match the exposure‐IDP GWEIS, we conduct a GWAS for the right putamen volume with the same covariates in the same 6858 participants. The Manhattan plots (upper right) show great differences between genetic variants with significant G × E interactions and those with significant genetic main effects. The four regional plots show details about the inconsistency. The lead variant rs9614084 (chr22) with significant interaction (*P* = 1.96 × 10^−8^) shows non‐significant genetic main effect (*P* = 0.81), while the lead variant rs8014725 (chr14) with significant genetic main effect (*P* = 1.75 × 10^−13^) shows non‐significant G × E interaction (*P* = 0.77). Abbreviations: Amp, amplitude; CT, cortical thickness; CTQ, childhood trauma questionnaire; FA, fractional anisotropy; FC, functional connectivity; FDR, false discovery rate; GMV, gray matter volume; GO, gene ontology; GWAS, genome‐wide association study; GWEIS, genome‐wide by environment interaction study; IDP, imaging‐derived phenotype; L, left; LD, linkage disequilibrium; R, right; ReHo, regional homogeneity; SA, surface area.

The 486 independent G × E interactions involved 401 LD‐independent loci, which were scattered throughout the autosomes (**Figure** **3**a), among which 72 loci were involved in different exposure‐IDP pairs. The most common sharing pattern was the same locus interacting with different exposures to influence different IDPs. For example, the locus 8p22 with lead variant rs13281312 interacted with autumn‐born to affect the ReHo of cerebellar vermis crus II, interacted with winter‐born to affect the FC between posterior default‐mode network and dorsal sensorimotor network, and interacted with NDVI to affect the CT of right inferior temporal cortex.

The 401 LD‐independent loci comprised 2611 unique variants with significant G × E interaction (inflation‐adjusted *P* < 5 × 10^−8^) in GWEISs, including 461, 387, 621, and 1142 variants for air pollution, climate, urbanicity, and family factors, respectively. We employed FUMA^[^ ^39^ ^]^ to perform functional annotations for these variants included in respective databases: assessed functional consequences using ANNOVAR^[^ ^40^ ^]^; identified deleterious variants using combined annotation‐dependent depletion (CADD) scores^[^ ^41^ ^]^; and searched for regulatory variants using RegulomeDB (RDB) scores.^[^ ^42^ ^]^ Of the 2569 variants included in the ANNOVAR database, 454, 383, 610, and 1122 variants were associated with air pollution, climate, urbanicity, and family factors, respectively. For each subgroup of variants, we assessed the enrichment of functional consequences (*P* < 0.05/9/4 = 0.0014, Bonferroni correction for nine consequences and four exposure categories) using ANNOVAR.^[^ ^40^ ^]^ Variants interacting with climate (55.09%, enrichment value = 1.50, *P* = 2.83 × 10^−13^) and urbanicity (44.92%, enrichment value = 1.23, *P* = 2.56 × 10^−5^) showed significant enrichment for intronic regions, while those interacting with family factors demonstrated significant enrichment for intergenic regions (65.06%, enrichment value = 1.40, *P* = 3.14 × 10^−36^; Figure 3b; Tables S10–S14, Supporting Information). We also found 14 non‐synonymous variants for 11 genes (Tables S10–S14, Supporting Information). Using a criterion of CADD > 12.37, from 2565 variants included in the CADD database, we identified 13, 5, 17, and 31 deleterious variants (Tables S10–S13, Supporting Information) for air pollution, climate, urbanicity, and family factors, respectively. Among the 2096 variants included in the RDB database, we found 18, 16, 17, and 28 variants with evidence (RDB < 4) for transcription factor binding (Tables S10–S13, Supporting Information) for air pollution, climate, urbanicity, and family factors, respectively.

We examined the overlap between variants with G × E interaction and expression quantitative trait loci (eQTL, *P* < 5 × 10^−8^) of brain tissues from a multi‐ancestry eQTL meta‐analysis.^[^ ^43^ ^]^ Among the 2611 variants, 1731 were included in the eQTL analysis. We found that 145 variants were eQTLs for 77 genes, of which 36, 18, 24, and 67 were interacted with air pollution, climate, urbanicity, and family factors, respectively (Table S15, Supporting Information). For instance, rs1202613, which interacts with only child status on right cerebellum VIIIa volume, is an eQTL for *ARV1* (*P* = 1.42 × 10^−59^). *ARV1* deficiency has been associated with autosomal recessive cerebellar ataxia, neurodegenerative disease, and developmental epileptic encephalopathy.^[^ ^38^, ^44^, ^45^ ^]^

### 2.6 Genes With G × E Interactions

Using Multivariate Analysis of Genomic Annotation (MAGMA),^[^ ^46^ ^]^ we conducted gene‐based interaction analyses to identify genes that exhibited significant interactions with exposures on brain IDPs (*P*  <  0.05/18 233 = 2.74 × 10^− 6^) based on GWEIS summary data. We found 1160 unique genes with G × E interaction on brain IDPs, including 257 genes with air pollution, 173 with climate, 285 with urbanicity, and 481 with family factors (Table S16, Supporting Information). For each set of genes, we further utilized STRING^[^ ^47^ ^]^ to conduct protein–protein interaction (PPI) analysis and gene ontology (GO) biological process enrichment analysis (*q* < 0.05, BH‐FDR corrected). Among the four PPI analyses, we identified two significant PPI networks. One significant PPI network (*P* = 0.0014) was observed for genes interacting with air pollution (Figure 3c), with *H4C6*, *SMARCA4*, and *RPS11* serving as the top three hubs. Another PPI network (*P* = 0.0011) was found for genes interacting with urbanicity exposures (Figure S5, Supporting Information), highlighting the top three hubs of *CCND1*, *CALM3*, and *CDK2*. Among the four gene sets, only genes interacting with air pollution showed significant enrichment for GO biological processes. These genes were mainly enriched in metal ion detoxification and homeostasis pathways, including detoxification of copper ion (*q* = 0.0019), cellular zinc ion homeostasis (*q* = 0.0021), cellular response to zinc ion (*q* = 0.0030), cellular response to copper ion (*q* = 0.0048), and cellular response to cadmium ion (*q* = 0.0223) (Figure 3d; Table S17, Supporting Information). These enrichments were driven primarily by the family of metallothionein genes, including *MT1X*, *MT1H*, *MT1M*, *MT1F*, *MT1A*, and *MT1B*, which formed a cohesive subnetwork (Figure 3c, red nodes) in the PPI analysis.

Among the 11 genes containing non‐synonymous variants, nine were significant in gene‐based analysis (*P* < 2.74 × 10^−^^6^), including *OR14C36*, *CEP85L*, *ASZ1*, *ZWINT*, *PDE2A*, *HHIPL1*, *ZNF286B*, *SRRM5*, and *A4GALT*. For example, rs28545014 (CADD = 22.5) at 1q44 showed an interaction with autumn‐born on the CT of the right lateral orbitofrontal cortex. The rs28545014 is a missense mutation in *OR14C36*, which is involved in odor perception, a critical function of the orbitofrontal cortex.^[^ ^48^ ^]^ Another example is rs341047 (CADD = 13.04) at 11q13.4, interacting with TD on the left hippocampal volume. This is a missense mutation in *PDE2A*, which plays a critical role in presynaptic short‐term plasticity in hippocampal neurons^[^ ^49^ ^]^ and demonstrates low hippocampal expression in patients with bipolar disorder.^[^ ^50^ ^]^

### 2.7 Relationship Between G × E Interactions and Genetic Main Effects

Based on the presumption that a genetic variant with a significant main effect on a given phenotype is more likely to show G × E interactions on this phenotype compared with those variants without significant main effects on this phenotype, several studies have investigated G × E interactions only for variants with significant main effects.^[^ ^51^, ^52^ ^]^ Here, we formally tested the hypothesis by examining the genetic main effects of the variants with significant G × E interactions (*P* < 5 × 10^−8^). Among the 486 independent G × E interactions (486 variant‐IDP pairs), we failed to identify any significant genetic main effects at *P* < 5 × 10^−8^ (Figure 3e; Table S18, Supporting Information). We estimated the proportion (*η* ^2^) of IDP variance explained by the genetic main effect for each of the 486 LD‐independent variant‐IDP pairs and the proportion (*η* ^2^) of IDP variance explained by G × E interaction for each of the 486 independent G × E interactions. We found that proportions (median: 0.50%; interquartile range (IQR): 0.40%–0.59%; Table S19, Supporting Information) of IDP variances explained by G × E interactions were larger (Wilcoxon sum rank test: *Z* = 26.99, *P* = 2.16 × 10^−160^; Figure 3f) than those (median: 0.0060%; IQR: 0.0015%–0.016%; Table S18, Supporting Information) explained by genetic main effects. These results indicate that the genetic variants with significant G × E interactions are not those with significant genetic main effects. For example, the variants with significant main effects on the right putamen volume were completely different from the variants influencing this IDP by interacting with CTQ‐emotional neglect (Figure 3g).

### 2.8 Environmental Exposures With G × E Interactions

**Figure 4:**
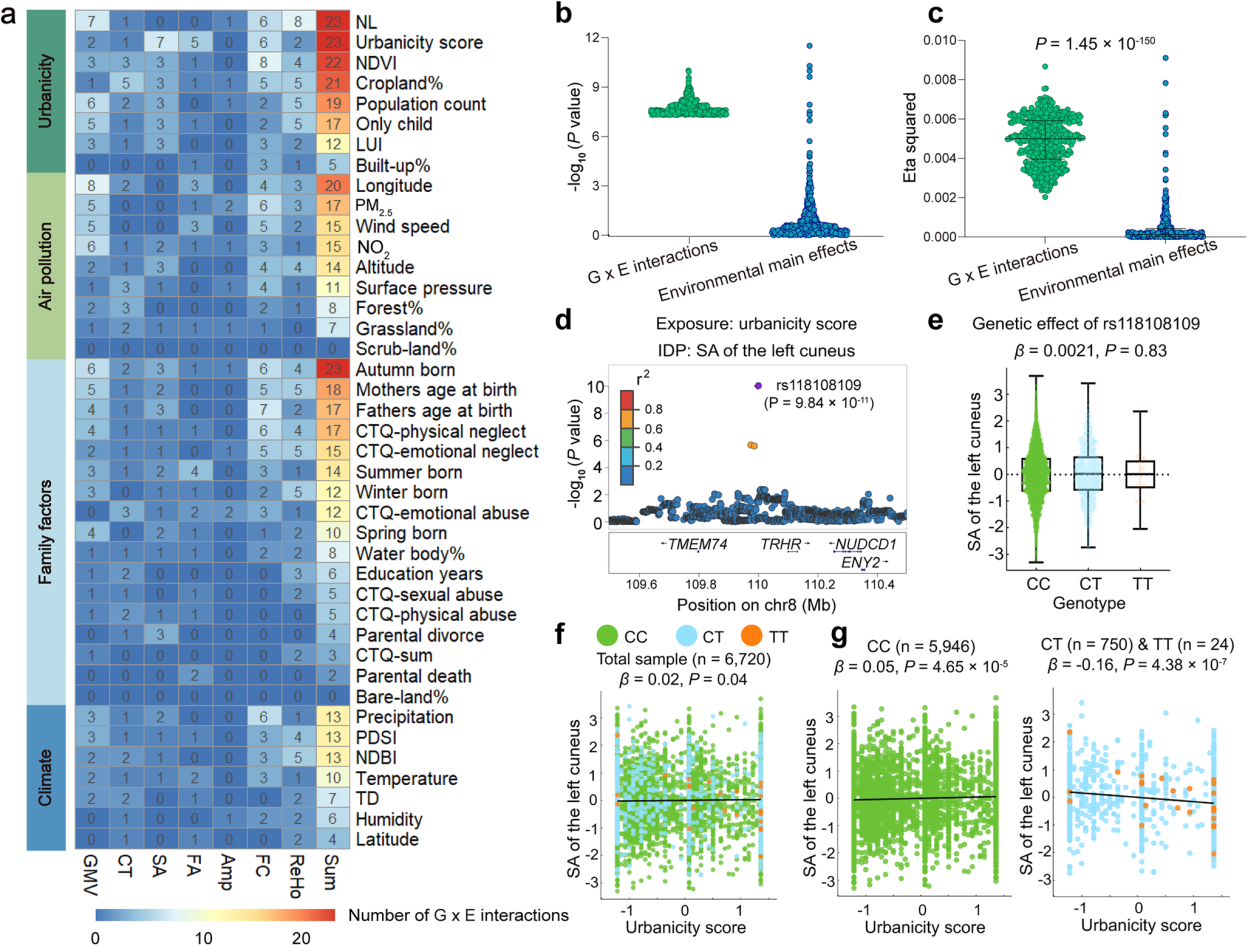
Environmental exposures with significant G × E interactions on brain IDPs. **a)** The heat map shows the number of significant G × E interactions of each exposure on each brain IDP category among the 486 independent G × E interactions. The exposures are ordered by exposure category. Among the 41 exposures, 39 show significant G × E interactions. **b,c)** The correspondence in significance **b**) and brain IDP explained variance **c**) between 486 G × E interactions (green) and 479 environmental main effects (blue). Only 17 (3.55%) exposures show significant correlations with the corresponding brain IDPs at a Bonferroni‐corrected *P* < 1.04 × 10^−4^ **b**). The Wilcoxon sum rank test indicates that G × E interactions explain much larger proportions of IDP variances (*Z* = 26.14, *P* = 1.45 × 10^−150^) than environmental main effects **c**). The three lines from bottom to top in violin plots indicate the first quartile, median, and third quartile. **d–g)** An example of inconsistency between G × E interaction and environmental main effect of urbanicity score in the variant‐IDP pair between rs118108109 (chr8) and left cuneus SA. Despite rs118108109 and urbanicity score show significant G × E interaction (*P* = 9.84 × 10^−11^) on the left cuneus SA **d**), neither genetic (*P* = 0.93; **e**) nor environmental (*P* = 0.04; **f**) main effect is significant. However, when testing the correlation between urbanicity score and left cuneus SA in genotype subgroups of rs118108109, we find a positive correlation (*β* = 0.05, *P* = 4.65 × 10^−5^; **g**) in CC‐carriers, but a negative correlation (*β* = −0.16, *P* = 4.38 × 10^−7^; **g**) in T‐allele carriers. Abbreviations: Amp, activity amplitude; CT, cortical thickness; CTQ, childhood trauma questionnaire; FA, fractional anisotropy; FC, functional connectivity; GMV, gray matter volume; IDP, imaging‐derived phenotype; LUI, land use intensity; NDBI, normalized difference built‐up index; NDVI, normalized difference vegetation index; NL, night‐time light; NO_2_, nitrogen dioxide; PDSI, Palmer drought severity index; PM, particulate matter; ReHo, regional homogeneity; SA, surface area; TD, temperature difference.

Except for the bare‐land% and scrub‐land%, 39 out of the 41 environmental exposures showed significant G × E interactions on brain IDPs, among which the NL, urbanicity score, and autumn born had the largest number (*n* = 23) of G × E interactions (**Figure** **4**a). Additionally, we calculated the interaction density for each exposure category, defined as the number of G × E interactions divided by the number of exposures in each category. The interaction densities, ranked from highest to lowest, were 17.75, 11.89, 10.06, and 9.43 for urbanicity, air pollution, family, and climate exposures, respectively. The 486 G × E interactions involved 479 exposure‐IDP pairs, of which six exposures showing significant interactions with different genetic variants on the same IDP. For example, NL interacted with rs1922046 at 7q21.11 (GWEIS: *P* = 2.34 × 10^−8^; score test: *q* = 3.43 × 10^−5^; BF rank: NL = 1) and rs112687750 at 9q34.11 (GWEIS: *P* = 1.68 × 10^−8^; score test: *q* = 2.14 × 10^−5^; BF rank: NL = 1) to influence the ReHo of the orbital part of right superior frontal gyrus (Figure S6, Supporting Information).

### 2.9 Relationship between G × E interactions and environmental main effects

For each of the 479 exposure‐IDP pairs, we investigated the main effect of the exposure on the IDP and identified 17 (3.55%) significant exposure‐IDP associations (*P* < 1.04 × 10^−4^, Bonferroni corrected for the 479 tests) (Figure 4b; Table S20 Supporting Information). For the 479 exposure‐IDP pairs, we estimated the proportions (*η* ^2^) of IDP variances explained by environmental main effects and compared with those explained by G × E interactions (*n* = 486). We also found that proportions (median: 0.50%; IQR: 0.40‐0.59%) of IDP variances explained by G × E interactions (Table S19, Supporting Information) were much larger (Wilcoxon sum rank test: *Z* = 26.14, *P* = 1.45 × 10^−150^; Figure 4c) than proportions of IDP variances (median: 0.013%; IQR: 0.0034%–0.043%) explained by environmental main effects (Table S20, Supporting Information). These results indicate most exposures show great inconsistency between G × E interactions and environmental main effects. Among the 486 independent G × E interactions, the most significant one was observed between rs118108109 at 8q23.1 and urbanicity score on the left cuneus SA (*P* = 9.84 × 10^−11^) in the univariate GWEISs, which was much more significant than the main effects of rs118108109 (*P* = 0.83) and urbanicity score (*P* = 0.04) (Figure 4d–f). The proportion (0.44%) of the phenotype variance explained by G × E interaction was much larger than those explained by the genetic (0.00045%) and environmental (0.043%) main effects. In *post‐hoc* analyses, we found a positive correlation (*β* = 0.05, *P* = 4.65 × 10^−5^) between urbanicity score and left cuneus SA in CC carriers, but a negative correlation (*β* = −0.16, *P* = 4.38 × 10^−7^) in carriers of the other two genotypes (CT and TT) (Figure 4g).

### 2.10 Brain IDPs With G × E Interactions

**Figure 5:**
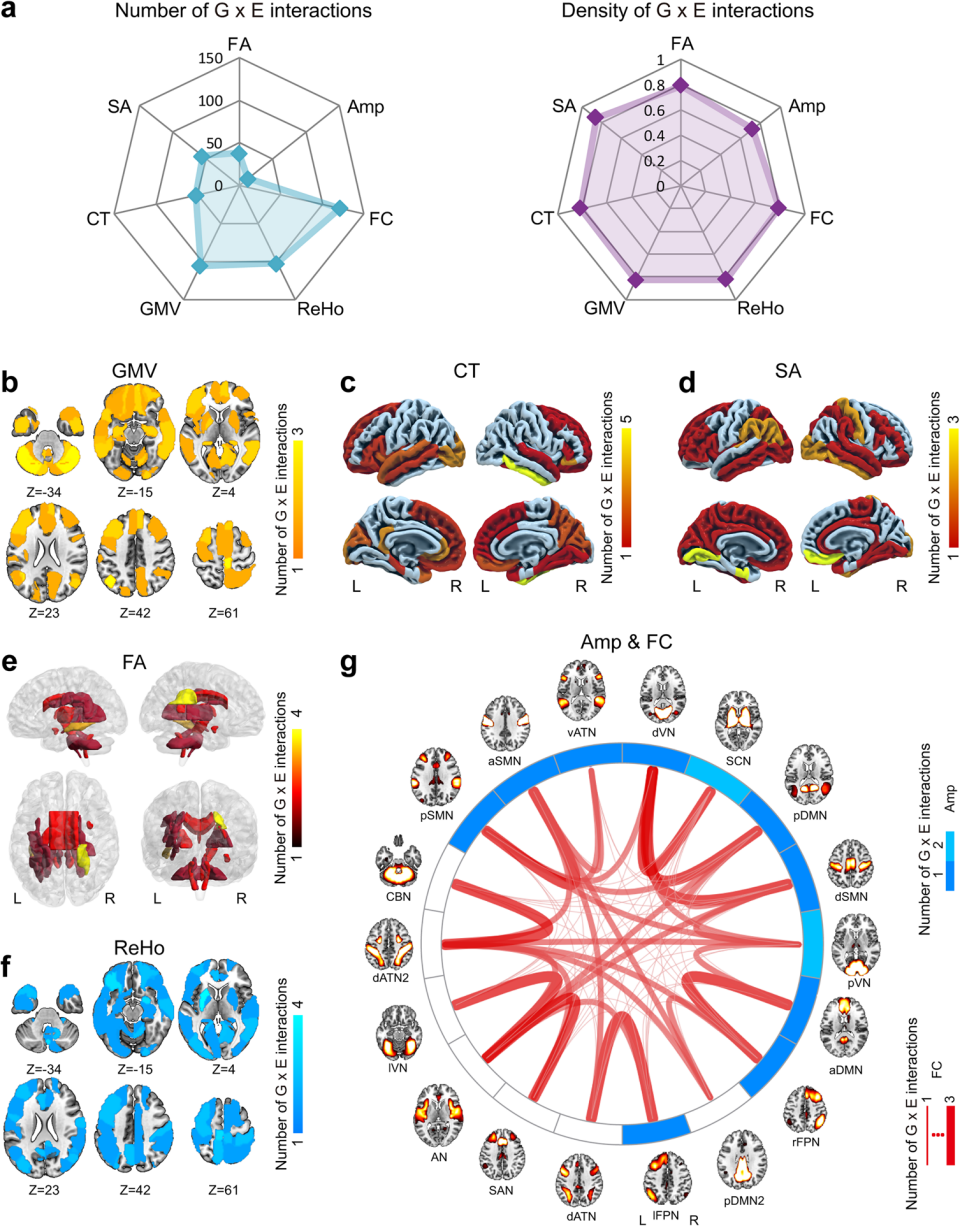
Spatial distribution of G × E interactions in the brain. **a)** The two radargrams show the number (left) and density (right) of G × E interactions for each IDP category among the 486 independent G × E interactions. For each IDP category, the interaction density is defined as the number of G × E interactions divided by the number of IDPs in this category. **b–g)** The spatial distribution of G × E interactions in the brain for each category of brain IDPs, including GMV **b**), CT **c**), SA **d**), FA **e**), ReHo **f**), Amp **g**), and FC **g**). In (**b–f)**, the color of each brain structure denotes the number of significant G × E interactions. In **g**, the circle color and the link thickness represent the number of significant G × E interactions on Amp within and FC between RSNs (brain maps around the circle). Abbreviations: aDMN: anterior default‐mode network; Amp: functional activity amplitude; AN: auditory network; aSMN: anterior sensorimotor network; CBN: cerebellar network; CT, cortical thickness; dATN: dorsal attentional network; dSMN: dorsal sensorimotor network; dVN: dorsal visual network; FA, fractional anisotropy; FC: functional connectivity; GMV, gray matter volume; IDP, imaging‐derived phenotype; L: left; lFPN: left frontal parietal network; lVN: lateral visual network; pDMN: posterior default mode network; pSMN: posterior sensorimotor network; pVN: primary visual network; R: right; rFPN: right frontal parietal network; ReHo, regional homogeneity; RSN: resting‐state network; SA, surface area; SAN: salience network; SCN: subcortical network; vATN: ventral attentional network.

This study included seven categories of MRI metrics and 598 brain IDPs (127 GMV, 64 CT, 64 SA, 48 FA, 18 Amp, 153 FC, and 124 ReHo), of which 335 IDPs (71 GMV, 31 CT, 43 SA, 24 FA, 11 Amp, 82 FC, and 73 ReHo) were identified to be influenced by G × E interactions. Among the 486 independent G × E interactions, 105, 52, 55, 38, 13, 121, and 102 influenced GMV, CT, SA, FA, Amp, FC, and ReHo IDPs, respectively (**Figure** **5**a). We also calculated the interaction density of each metric, which was defined as the number of significant interactions of this metric category divided by the number of all IDPs in this metric category. We found that the interaction densities of GMV, CT, SA, FA, Amp, FC, and ReHo were 0.83, 0.81, 0.86, 0.79, 0.72, 0.79, and 0.82, respectively, which showed approximately a uniform distribution (Figure 5a).

Among the 335 IDPs with significant G × E interactions, 116 were influenced by two or more G × E interactions. For example, the CT of right inferior temporal gyrus was influenced by interactions between rs2046096 (8p22) and NDVI (*P* = 9.75 × 10^−9^), rs11301877 (9q22.32) and forest% (*P* = 2.70 × 10^−8^), rs10774359 (12p13.31) and CTQ‐emotional abuse (*P* = 4.06 × 10^−8^), rs148904967 (15q21.1) and LUI (*P* = 4.97 × 10^−8^), and rs3746002 (19q13.31) and education years (*P* = 9.68 × 10^−9^). These significant G × E interactions involved five different autosomes and five different exposures, indicating the scattered distribution feature of G × E interactions across the genome and exposome. The spatial distribution of G × E interactions in the brain was summarized for each MRI metric (Figure 5b–g). We found that G × E interactions were generally scattered in the brain for all IDP categories, although some IDPs had more interactions.

### 2.11 Sensitive Periods of G × E Interactions

For each time‐varying exposure included in the verified G × E interactions, we used the distributed lag model (DLM)^[^ ^53^ ^]^ to identify the sensitive periods of G × E interactions in the first 18 years of age. DLM was conducted in the participants who had complete exposure data for the first 18 years of age (Figure S1 and Table S3, Supporting Information). Among the 486 verified G × E interactions, 245 interactions involved time‐varying exposures (*n* = 19), including 194 interactions (16 exposures) with sufficient samples (3049–5686) for the sensitive period analysis. We then applied DLM to the 194 G × E interactions, and defined the sensitive period as the age windows during which the estimated pointwise 95% confidence intervals (CI) did not include zero.

**Figure 6:**
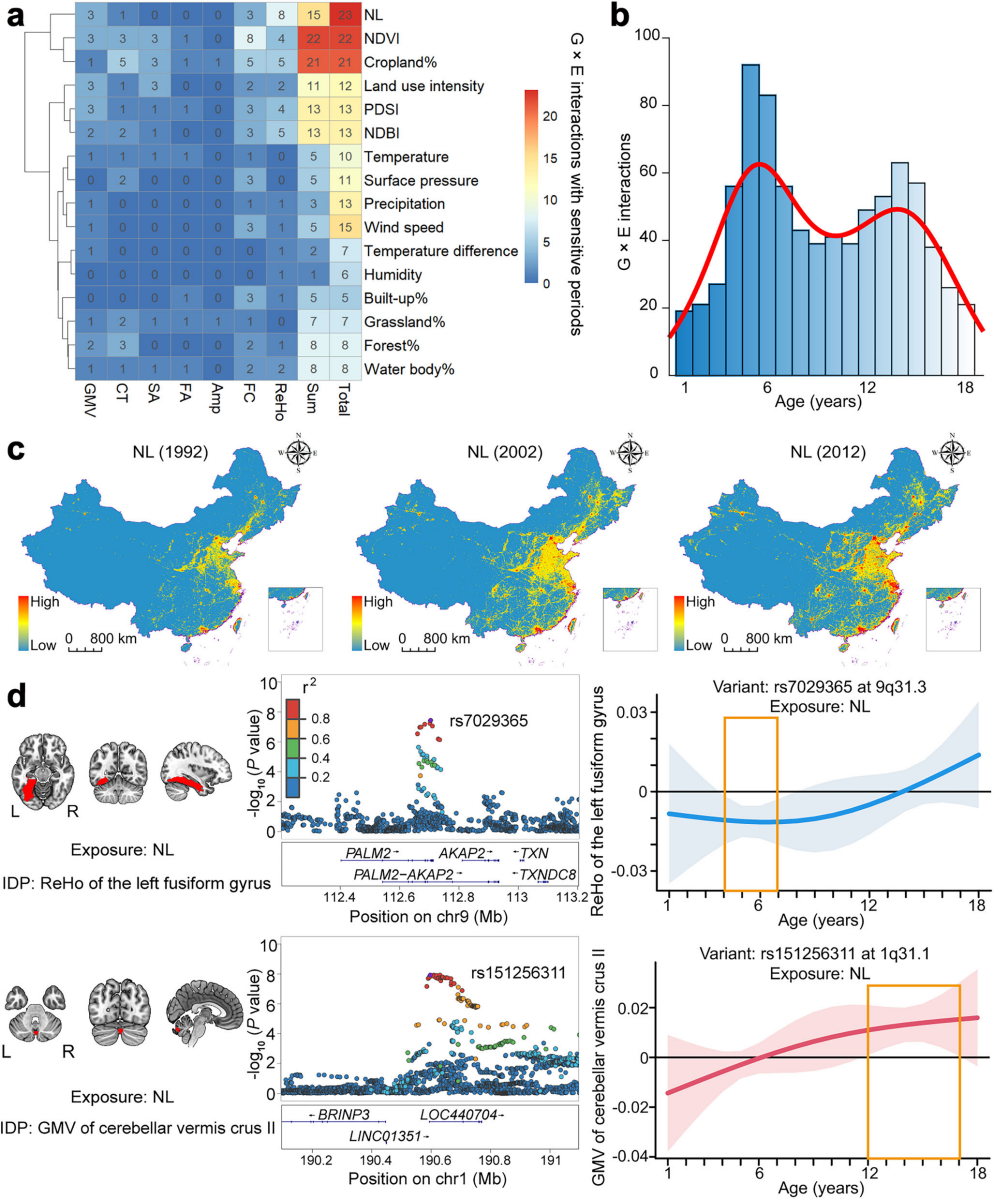
Sensitive periods of G × E interactions on brain IDPs. **a)** The heat map shows the distribution of 144 independent G × E interactions with sensitive periods across the 16 time‐varying exposures and the seven categories of brain IDPs. The exposures are ordered by hierarchical clustering. **b)** The histogram shows the overall frequency distribution of sensitive periods of the 144 G × E interactions in the first 18 years of age. Sensitive periods of G × E interactions are more frequently seen during childhood (4‐7 years) and adolescence (12‐15 years). **c)** The geographic maps show the spatial distribution of annually averaged NL data in China in 1992, 2002, and 2012. **d)** Two representative examples of sensitive periods of G × E interactions on brain IDPs. The sensitive period of the interaction between rs7029365 and NL on the ReHo of the left fusiform gyrus is observed in 4–7 years of age (upper panel), while the sensitive period of the interaction between rs151256311 and NL on the GMV of the cerebellar vermis Crus II is observed in 12–17 years of age (lower panel). The left panel shows the location of each brain IDP, the middle panel shows the variants with significant interaction with NL on the IDP, and the right panel shows the sensitive period for G × E interaction on the IDP. A sensitive period is defined as a period during which the estimated pointwise 95% confidence intervals (CI) does not include zero. Abbreviations: Amp, activity amplitude; CT, cortical thickness; FA, fractional anisotropy; FC, functional connectivity; GMV, gray matter volume; IDP, imaging‐derived phenotype; L, left; NDBI, normalized difference built‐up index; NDVI, normalized difference vegetation index; NL, night‐time light; PDSI, Palmer drought severity index; R, right; ReHo, regional homogeneity; SA, surface area.

Among the 194 G × E interactions (16 exposures), 144 (74.23%) showed sensitive periods (**Figure** **6**a; Figures S7–S13, and Table S21, Supporting Information), involving all the 16 exposures. However, the proportions of G × E interactions with sensitive periods were different across these exposures. For instance, the sensitive period was observed in all 22 (100%) G × E interactions involving NDVI, but only in 3 (23%) out of 13 G × E interactions involving precipitation (Figure 6a). Based on the 144 interactions, we calculated the appearance frequency of sensitive periods for each year of age (1–18 years), and identified two age windows (4–7 years and 12–15 years) with more frequent appearance of sensitive periods than other age windows (Figure 6b). We also stratified the frequency distribution of sensitive periods for G × E interactions by IDP categories, and found the typical two‐peak distribution for CT, FC, and ReHo metrics (Figure S14, Supporting Information). When we stratified the frequency distribution by exposures, we also identified the two‐peak distribution for several exposures (Figure S15, Supporting Information), such as the NL, cropland%, NDVI, and PDSI. Taking the time‐varying exposure NL (Figure 6c) as an example, we found that the sensitive period of the interaction between rs7029365 at 9q31.3 and NL on the ReHo of the left fusiform gyrus was in childhood (age 4–7 years; Figure 6d), while the sensitive period of the interaction between rs151256311 at 1q31.1 and NL on the GMV of the cerebellar vermis crus II was during adolescence (age 12–17 years; Figure 6d).

As for the other three time‐varying exposures (NO_2_, PM_2.5_, and population count) with 51 independent G × E interactions, only a few participants (*n* < 500) had complete exposure data for the first 18 years of age due to data missing for the earlier and/or later years of age (26% missing data for NO_2_, 27% for PM_2.5_, and 38% for population count). To ensure more than 500 participants in each DLM analysis, we had to remove the first three, one, and three years of age in the DLM analyses for NO_2_, PM_2.5_, and population count, respectively. We performed DLM analyses for the 51 verified G × E interactions associated with the three exposures in 546–800 participants, and found sensitive periods for 16 G × E interactions (Figure S16 and Table S22, Supporting Information).

### 2.12 Interaction Between Polygenic Risk Score (PRS) and Exposure

We also examined interactions between PRS scores for neuropsychiatric disorders and environmental exposures on brain IDPs. We included EAS‐GWAS summary statistics of seven major neuropsychiatric disorders: anxiety,^[^ ^54^ ^]^ Alzheimer's disease,^[^ ^55^ ^]^ bipolar disorder,^[^ ^56^ ^]^ major depressive disorder (MDD),^[^ ^57^ ^]^ Parkinson's disease,^[^ ^58^ ^]^ schizophrenia,^[^ ^59^ ^]^ and stroke.^[^ ^60^ ^]^ PRS scores for CHIMGEN participants were calculated using PRS‐CS^[^ ^61^ ^]^ and PLINK. We identified significant interactions (*P* < 0.05/7/41/598 = 2.91 × 10^−7^, Bonferroni corrected) between seven PRS scores and 41 environmental exposures on 598 brain IDPs, but failed to identify any significant interactions (Table S23, Supporting Information). The most significant interaction (*P* = 1.04 × 10^−6^) was found between stroke PRS and CTQ‐emotional abuse on the GMV of the left middle frontal gyrus (orbital).

## 3 Discussion

Although many studies have investigated the genetic or environmental effects on brain imaging phenotypes, little attention was paid to their complex interactions. All previous G × E interaction studies on brain IDPs were conducted for a few candidate variants, exposures, and IDPs in only hundreds of participants,^[^ ^31^, ^32^, ^33^ ^]^ which could not provide an unbiased understanding of G × E interactions on brain IDPs. Therefore, we conducted the genome‐wide G × E interaction studies with 41 distinct exposures on 598 brain IDPs in 7084 healthy young adults. We identified 703 genome‐wide significant interactions in univariate analyses and confirmed 486 interactions in multivariate analyses, scattered across the genome, exposome, and phenome. Among the 486 G × E interactions, most (96.45%) environmental and all genetic main effects were non‐significant. Of the 194 interactions involving the time‐varying exposures, we found sensitive periods for 144 interactions, mainly during childhood (4–7 years) and adolescence (12–15 years). Their inconsistencies with genetic and environmental main effects highlight the value of the genome‐wide by exposome‐wide interaction studies of brain phenotypes. The identified G × E interactions on brain structure and function and their sensitive periods may guide precise interventions for optimizing brain health.

This study investigated the relationships between main and interaction effects. A strategy of prior G × E interaction studies is to first identify the genetic variants with significant main effects on a given phenotype, and then investigate their interactions on this phenotype. This strategy is based on an unproven hypothesis that a genetic variant with a significant main effect is more likely to show significant G × E interactions. In this study, we rejected the hypothesis by showing that none of the genetic variants in the variant‐IDP pairs with G × E interactions showed genome‐wide significant main effects and that these genetic main effects explained less variance of brain IDPs than G × E interactions. Selecting exposures with environmental main effects for interaction studies is also problematic, as only 3.55% exposures with G × E interactions showed significant main effects, and that 486 interactions explained much more variances of IDPs than corresponding environmental main effects. The dissociation between G × E interactions and genetic and environmental main effects on brain IDPs highlights the importance of the unbiased identification of G × E interactions on brain phenotypes across the genome and the exposome.

We found that variants interacting with climate, urbanicity, and family factors were significantly enriched in intergenic and intronic regions. We also identified 79 and 145 variants with significant interactions involved in transcription factor binding and gene expression in brain tissues, respectively. These findings indicate that regulatory variants in non‐coding regions could influence brain structure and function through interacting with environmental exposures. A possible mechanism is that exposures may affect brain IDPs by regulating gene expression via epigenetic mechanisms.^[^ ^29^, ^30^ ^]^ Furthermore, we found 14 non‐synonymous and 66 deleterious variants that could affect brain IDPs by interacting with exposures. These variants deserve particular attention because their pathogenic effects have been established.^[^ ^41^ ^]^

We found that genes interacting with different exposure categories were involved in distinct PPI networks, featuring unique hub genes. *SMARCA4*, *H4C6*, and *RPS11* were hub genes for the PPI network associated with air pollution. *SMARCA4* mobilizes nucleosomes and plays key roles in gene transcription regulation, development, genome replication, and genome repair.^[^ ^62^ ^]^ *SMARCA4* has been linked to various brain tumors^[^ ^63^ ^]^ and autism spectrum disorders.^[^ ^64^ ^]^ *H4C6* belongs to the histone H4 family and involves in transcription regulation, DNA repair and replication, and maintaining chromosomal stability. Its dysfunction has been linked to intellectual disability and developmental delay.^[^ ^65^ ^]^ *RPS11* is involved in ribosome biogenesis, and has been related to Alzheimer's disease.^[^ ^66^ ^]^ In contrast, *CCND1*, *CALM3*, and *CDK2* were hub genes for the PPI network related to urbanicity. *CCND1* shows altered expression in neurodegenerative diseases, including Alzheimer's disease,^[^ ^67^ ^]^ vascular dementia,^[^ ^68^ ^]^ and Parkinson's disease.^[^ ^69^ ^]^ *CDK2* regulates cell cycle and shows elevated expression in the brains of individuals with mild cognitive impairment.^[^ ^70^ ^]^ *CALM3* encodes calmodulin, a key calcium‐binding protein that plays a crucial role in calcium‐mediated signaling.

Genes interacting with air pollution were enriched in metal ion detoxification and homeostasis pathways, which were primarily driven by metallothionein genes (*MT1X*, *MT1H*, *MT1M*, *MT1F*, *MT1A*, and *MT1B*), forming a cohesive PPI subnetwork. These genes, acting as key regulators of metal ion detoxification and homeostasis pathways, play an essential role in supporting neuronal regeneration and maintaining cognitive function.^[^ ^71^ ^]^ Air pollution contains enormous particulate metals that can enter the human body through inhalation, which may result in reactive oxidative stress and inflammation by disturbing metal ion homeostasis, a central mechanism underlying the toxicity of air pollution.^[^ ^72^ ^]^ Therefore, our findings indicate that interactions between metallothionein‐related variants and air pollution exposures may influence brain IDPs through the same pathogenic pathways. For instance, individuals with certain metallothionein‐related risk genotypes may show a reduced ability to mitigate metal‐induced neurotoxicity, leading to heightened susceptibility to neurotoxicity associated with air pollution. Approaches aimed at reducing air pollution exposure, enhancing metal chelation, and strengthening antioxidant capacity may help mitigate neurotoxic effects in this subpopulation.

We also revealed sensitive periods for 144 G × E interactions on brain IDPs. These sensitive periods for interactions on brain IDPs appeared mainly during childhood (4–7 years) and adolescence (12–15 years), which are consistent with the sensitive periods identified for the environmental main effects on human brain and behavior^[^ ^23^, ^24^, ^26^, ^73^, ^74^, ^75^, ^76^, ^77^, ^78^ ^]^ and critical periods for normal development of brain, cognition, and emotion.^[^ ^75^, ^79^, ^80^, ^81^ ^]^ Childhood (4–7 years) coincides with a period of rapid synaptic pruning and refinement in the prefrontal and association cortices, which underlie the development of executive functions, social cognition, and emotion regulation.^[^ ^80^, ^82^, ^83^ ^]^ Adolescence (12–15 years) is another critical period of development, characterized by enhanced synaptic plasticity, extensive brain reorganization, and an imbalance between the more mature subcortical areas and the less mature prefrontal areas.^[^ ^84^, ^85^ ^]^ Both periods are marked by heightened neural plasticity,^[^ ^86^ ^]^ which may make the brain particularly susceptible to both beneficial and adverse environmental influences, especially in individuals with specific genetic backgrounds. In addition, the two sensitive periods are consistent with the onset ages of common mental disorders,^[^ ^87^, ^88^, ^89^, ^90^ ^]^ such as childhood for autism spectrum disorder and attention deficit and hyperactivity disorder, and adolescence for schizophrenia and MDD. As several exposures and brain imaging phenotypes involving G × E interactions have been associated with these mental disorders,^[^ ^91^, ^92^, ^93^, ^94^, ^95^, ^96^ ^]^ the sensitive periods for G × E interactions on brain IDPs may facilitate the advancement of new hypotheses for mental disorders. For instance, we revealed that adolescence (13–16 years) was the sensitive period for the interaction between rs8074097 and built‐up% (a proxy of urbanicity) on the left insula volume, based on which we hypothesize that urbanicity may interact with the variant to facilitate the onset of schizophrenia and MDD because high urbanicity is a risk factor for and left insula volume reduction is a critical feature of these two disorders.^[^ ^91^, ^92^, ^93^, ^94^ ^]^ If this hypothesis is verified, we may propose more precise prevention for these disorders by reducing the risk exposures embedded in urbanicity^[^ ^97^ ^]^ at adolescence in genotype carriers that are more sensitive to urbanicity.

Our results highlight a promising path toward personalized intervention strategies, achieved through the precise identification of both genetically susceptible individuals and critical sensitive periods. For instance, we observed an interaction between NO_2_ and rs57154176 on FA in the right posterior limb of the internal capsule. FA serves as a biomarker of white matter microstructural integrity, and FA reduction in this tract has been linked to sensorimotor deficits. *Post‐hoc* analyses revealed that the association between NO_2_ and FA‐IDP was exclusively present among individuals carrying at least one minor allele (G) (AG/GG genotypes: *β* = −0.21, *P* = 5 × 10^−7^, *n* = 713), while it was absent in AA homozygotes (*β* = −0.01, *P* = 0.51, *n* = 4,788). This compelling contrast underscores how genetic susceptibility modulate the effects of environments on white matter microstructure. Furthermore, we identified adolescence (ages 12–15) as the most critical sensitive period for this G × E interaction, suggesting that neurodevelopmental processes during adolescence may heighten the brain's vulnerability to NO_2_ exposure in genetically predisposed individuals. Therefore, our analyses may provide not only the targeted individuals but also the timeframe for intervention. By integrating genetic profiling with temporally precise environmental assessments, our findings may shift from population‐wide recommendations toward strategies tailored to an individual's genetic background and developmental stage, ultimately advancing more effective and equitable preventive interventions.

Two significant limitations must be considered when interpreting our results. First, we did not correct for the number of brain IDPs and exposures in univariate GWEISs and gene‐based analyses due to the limited statistical power, which may result in false positive findings. Although we used multivariate methods to verify G × E interactions identified by the univariate GWEISs, we cannot entirely rule out the possibility of false positive results. Second, the absence of both internal and external independent data validation may impede our ability to generalize our results. We did not perform internal independent data validation due to the limited sample size, while we refrained from conducting external independent data validation because of substantial differences in age range, environmental assessments, and genetic ancestry between CHIMGEN and other available large‐scale datasets, such as the UK Biobank study^[^ ^98^ ^]^ and the Adolescent Brain Cognitive Development study.^[^ ^99^ ^]^ Despite these limitations, we believe that this study still represents significant progress in understanding G × E interaction effects on brain structure and function, as it is the first attempt to identify genome‐wide significant G × E interactions for various exposures and brain IDPs, especially sensitive periods.

## 4 Experimental Section

### Participants

All participants were recruited from the Chinese Imaging Genetics (CHIMGEN) study, which included the genome, exposome, and neuroimaging data of 7306 Chinese Han participants (2601 males and 4615 females) aged 18–30 years. They were recruited from 32 research centers (Table S24, Supporting Information) according to the predefined inclusion and exclusion criteria (Table S25, Supporting Information). Informed consent was obtained from each participant, and this study was approved by the local ethical committees of all research institutions.

### Genome Data Acquisition and Preprocessing

Among the 7306 CHIMGEN participants, 7195 with DNA samples were genotyped by Illumina ASA‐750K (Asian Screening Array) specially designed for Asian people. After aligning the genotyped data to the human reference genome (GRCh37/hg19), PLINK2^[^ ^36^ ^]^ (http://www.cog‐genomics.org/plink/2.0/) was used for performing genetic data quality control. Details for the sample‐level and variant‐level quality control, genetic principal component analysis (PCA), and genetic data imputation are provided in a prior study.^[^ ^100^ ^]^ A total of 7163 participants remained after quality control. Of the 11 137 0847 imputed autosomal variants, 54 61901 with minor allele frequency (MAF) ≥ 0.05 and imputation information score (INFO) ≥ 0.9 were included in our GWEISs. The MAF threshold was used to ensure that the minimal genotype subgroup had enough samples for conducting statistical analysis, while the INFO threshold was used to ensure the high imputation quality of the included variants. Among the imputed 3685445 X‐chromosomal variants, 14 53 70 variants in the non‐pseudoautosomal region (non‐PAR) and 2261 in the two pseudoautosomal regions (PARs) remained with filters of MAF ≥  0.05 and INFO  ≥  0.9. MAF and INFO values were obtained using QCTOOL (version 2.0.8; https://www.well.ox.ac.uk/~gav/qctool_v2/) with the ‘‐infer‐ploidy‐from sex’ option, which generated values for non‐PAR variants based on half‐contribution of males relative to females.

### Exposome Data Acquisition and Preprocessing

The exposome was assessed using 41 environmental exposures (21 time‐varying and 20 time‐invariant exposures; Table S2, Supporting Information). The time‐varying exposures were the population count,^[^ ^101^ ^]^ normalized difference built‐up index (NDBI),^[^ ^102^ ^]^ normalized difference vegetation index (NDVI),^[^ ^103^ ^]^ nighttime lights (NL),^[^ ^104^ ^]^ particular matter 2.5 (PM_2.5_),^[^ ^105^ ^]^ nitrogen dioxide (NO_2_),^[^ ^106^ ^]^ Palmer drought severity index (PDSI),^[^ ^107^ ^]^ precipitation,^[^ ^108^ ^]^ humidity,^[^ ^109^ ^]^ surface pressure,^[^ ^109^ ^]^ wind speed,^[^ ^108^ ^]^ temperature,^[^ ^109^ ^]^ temperature difference (TD), bare‐land%,^[^ ^110^ ^]^ cropland%,^[^ ^110^ ^]^ grassland%,^[^ ^110^ ^]^ forest%,^[^ ^110^ ^]^ scrub‐land%,^[^ ^110^ ^]^ built‐up%,^[^ ^110^ ^]^ water body%,^[^ ^110^ ^]^ and land use intensity (LUI).^[^ ^111^ ^]^ The 20 time‐invariant exposures included the four binary variables (spring, summer, autumn, and winter) of birth season, altitude, latitude, and longitude of birthplace, urbanicity score,^[^ ^24^ ^]^ education years, father's and mother's ages at birth, only‐child status, parental divorce, parental death, and emotional abuse, physical abuse, sexual abuse, emotional neglect, physical neglect, and total score of Childhood Trauma Questionnaire (CTQ).^[^ ^112^ ^]^ The distributions of exposure data are presented in Figure S17 (Supporting Information). Details about the assessment and quality control of the 41 environmental exposures are described in Supporting Information. Briefly, based on the annual residential coordinates of the first 18 years of age of each participant, the annual data of the 21 time‐varying exposures and altitude, latitude and longitude of birthplace was obtained from the satellite remote sensing data provided by Google Earth Engine (GEE),^[^ ^113^ ^]^ Social Economic Data and Applications Center (SEDAC), and annual China's land use/land cover datasets (CLUD‐A).^[^ ^110^, ^114^ ^]^ The other 17 time‐invariant exposures were obtained from the paper‐based questionnaires.

Based on the Spearman correlations among the 41 environmental exposures, hierarchical clustering was conducted to group them into distinct categories. The optimal number of exposure categories was determined using the Calinski‐Harabasz (CH) criterion,^[^ ^115^ ^]^ assessing how similar an exposure is to the category it belongs to compared to other categories. As a higher CH value indicates larger between‐category variance and smaller within‐category variance, the optimal number of categories corresponds to the highest CH value.

### Brain MRI Data Acquisition and Preprocessing

Brain MRI data were acquired by ten types of 3.0‐Tesla MRI scanners and 12 sets of scanning parameters (Tables S26–S28, Supporting Information). The structural MRI (sMRI) data were used to calculate gray matter volume (GMV), cortical thickness (CT), and surface area (SA) of each region to assess the macrostructural properties of brain gray matter. The diffusion MRI (dMRI) data were used to calculate the fractional anisotropy (FA) of each tract to assess the microstructural integrity of brain white matter. The resting‐state functional MRI (fMRI) data were used to calculate functional activity amplitude (Amp) within each resting‐state network (RSN), functional connectivity (FC) between RSNs, and regional homogeneity (ReHo) of spontaneous neuronal activity, to assess the brain functional properties. The details about MRI data preprocessing, quality control, and brain imaging‐derived phenotypes (IDPs) calculation were provided in the Supporting Information. The 598 IDPs, including 127 GMV‐IDPs, 64 CT‐IDPs, and 64 SA‐IDPs from 7182 participants, 48 FA‐IDPs from 7153 participants, and 18 Amp‐IDPs, 153 FC‐IDPs, and 124 ReHo‐IDPs from 6283 participants (Table S1, Supporting Information) were finally included.

In contrast to including imaging sites as a covariate, a more effective method (ComBat harmonization^[^ ^116^ ^]^) was utilized to reduce the scanner effect by harmonizing IDPs calculated based on the MRI data acquired by different scanners. Taking 64 CT‐IDPs and 124 ReHo‐IDPs as two examples, the effect of ComBat harmonization was tested in two participants who traveled across centers and were scanned at 28 MRI scanners. In each participant, these IDPs were calculated based on the MRI data acquired from each scanner and computed Spearman correlation coefficients of the 64 CT‐IDPs and the 124 ReHo‐IDPs between MR scanners. The Wilcoxon sum rank test was used to compare the correlation coefficient differences before and after harmonization, and found that the correlation coefficients of the IDPs after harmonization were much larger than those before harmonization (Figure S18, Supporting Information). The distribution of the seven IDP categories across different scanners before and after harmonization are presented in Figures S19–S25 (Supporting Information). Then, a normal score transformation was applied to the harmonized IDPs to make the data normally distributed and to reduce the undue influence of outliers.

### Univariate G × E Interaction Analyses

An univariate gene‐environment (G × E) interaction analyses were performed between each pair of 54 61 901 autosomal genetic variants and 41 exposures on 598 IDPs in all participants with complete genetic, environmental, and imaging data using PLINK2 (http://www.cog‐genomics.org/plink/2.0/).^[^ ^36^ ^]^ The univariate G × E interaction model could be written as Equation (1):

(1) $$y=\mu +\beta_{G}x_{G}+\beta_{E}x_{E}+\beta_{GE}x_{G}x_{E}+\beta_{C}x_{C}+\varepsilon$$

where *y* is an IDP, *μ* is the intercept, *x_G_* is a standardized variant dosage, *x_E_* is a standardized exposure, *x_G_x_E_* is the interaction term, *x_C_* is the standardized covariate matrix, *β*_*G*_, *β*_*E*_, *β*_*GE*_, *β*_*C*_ are their standardized coefficients, and *ε* is the residual. An additive interaction model was fit at each variant with allelic dosage from the imputed data. The age, sex, and first 10 genetic principal components (PCs) were included as nuisance covariates. Total intracranial volume (TIV), total surface area, mean cortical thickness, and mean framewise displacement (FD)^[^ ^117^ ^]^ were controlled for IDP analyses of GMV, SA, CT, and fMRI phenotypes, respectively. For X‐chromosomal variants, the only distinction from autosomal variants was the non‐PAR variants, where the dosages of variants for males were scaled by a factor of 2 (with the ‘–xchr‐model 2’ option), resulting in a range of 0–2. Considering the tremendous computational resources needed, these analyses were conducted on the TianHe‐1A supercomputer (https://www.nscc‐tj.cn).

After obtaining 24518 GWEIS summary statistics (598 IDPs × 41 exposures), the genomic‐control inflation factor (λ_GC_)^[^ ^37^ ^]^ for each significant GWEIS (*P* < 5 × 10^−8^) was estimated and adjusted *P* values using PLINK2 to correct the genome‐wide inflation and false positive rate. Based on the significant GWEISs (inflation‐adjusted *P* < 5 × 10^−8^, no further correction for the numbers of brain IDPs and exposures), the LD‐independent G × E interactions and LD‐independent variant‐IDP pairs by PLINK clumping were identified. To find LD‐independent G × E interactions from each significant GWEIS (exposure‐IDP pair), PLINK clumping was utilized to integrate all variants showing significant interaction (*P* < 5 × 10^−8^) with the exposure on the IDP into LD‐independent variants: a) all significant variants were included in a list of candidate variants; b) the most significant variant was defined as the lead variant and other variants within 500 kb from and in LD (*r^2^* > 0.1) with the lead variant were clumped; c) the remaining variants formed a new list of candidate variants and then step b) was repeated; and d) the iterative process stopped until the list was empty. The LD‐independent variant‐IDP pair was also identified by clumping genetic variants showing significant interaction (*P* < 5 × 10^−8^) with any exposure on this IDP.

Given the collinearity among exposures, a conditional G × E interaction test was used to identify independent G × E interactions.^[^ ^118^, ^119^ ^]^ Specifically, after obtained LD‐independent G × E interactions and LD‐independent variant‐IDP pairs, the following iterative conditional analyses was implemented to identified independent G × E interactions: a) all LD‐independent G × E interactions involved in the same variant‐IDP pair were included in a candidate list; b) the most significant interaction was selected as the independent G × E interaction, and all other candidate interactions were re‐tested while additionally conditioning on the most significant LD‐independent G × E interaction; c) the remaining significant interactions (*P* < 5 × 10^−^⁸) formed a new candidate list, and step b) was repeated; and d) the iteration continued until no significant interactions remained.

### Multivariate G × E Interaction Analyses

The multivariate structured linear mixed model (StructLMM)^[^ ^35^ ^]^ was used to validate the G × E interactions identified by univariate GWEISs. StructLMM included all these 41 exposures in the same model to estimate the overall interaction between one variant and all exposures on each IDP. The multivariate model could be written as Equation (2):

(2) $$y=Xb+G\beta_{G}+G⊙\beta_{G\times E}+e+\Psi$$

where *y* is an IDP, *X* is the covariate matrix, *b* is the fixed effect of covariates, *G* is the vector of variant dosage, *β*_*G*_ is the effect size of a conventional persistent genetic effect component, *β*_*G* × *E*_ is the vector of random effect accounting for the multivariate G × E interaction, ⊙ denotes the element‐wise (Hadamard) product, *e* is the random effect of the environment matrix, and *Ψ* is the noise term.

In this model, the score test can determine whether one variant shows significant interaction with the 41 exposures on a brain IDP, which was utilized to verify each LD‐independent variant‐IDP pair identified by the univariate analyses. In the score test, the statistical significance was adjusted for the total number of variant‐IDP pairs using a Benjamini‐Hochberg false discovery rate (BH‐FDR) approach, and *q* < 0.05 (BH‐FDR corrected) was considered as successful validation. For each variant‐IDP pair, the model can also output the Bayes factor (BF) of each exposure to represent the evidence for the exposure in driving the overall G × E interaction for the variant‐IDP pair. As a verified LD‐independent variant‐IDP pair may include several significant G × E interactions in the univariate analyses, an independent G × E interaction was considered to be verified if the BF value of the exposure was ranked no larger than the total number of significant interactions identified for the variant‐IDP pair in the univariate GWEISs.

Based on all the verified LD‐independent variant‐IDP pairs for each IDP, the LD‐independent locus‐IDP pairs for this IDP were identified by the following steps: a) creating loci for all LD‐independent variants for the IDP by adding 500 kb to both sides; b) merging overlapping loci; c) merging loci if an independent variant was in LD (*r* ^2^ > 0.1) with any LD‐independent variant of other loci; and d) merging loci overlapped with major histocompatibility complex (MHC) region (hg19 location chr 6: 25119106‐33854733) or chromosome 8p23.1 (7242715‐12483982) into one locus. For the verified variant‐IDP pairs, the LD‐independent variants were identified by clumping all variants in the variant‐IDP pairs and the LD‐independent loci by clumping all loci in the locus‐IDP pairs. The same strategies were used to identify LD‐independent variants and loci for the verified independent G × E interactions.

### Functional Annotation for Genetic Variants With G × E Interactions

For the verified LD‐independent loci, all autosomal variants significantly interacting with each category of exposures (inflation‐adjusted *P* < 5 × 10^−8^) were extracted from the univariate GWEISs. FUMA (https://fuma.ctglab.nl/)^[^ ^39^ ^]^ was applied to perform functional annotations for these variants included in respective databases. Specifically, ANNOVAR^[^ ^40^ ^]^ was utilized to evaluate functional consequences, combined annotation‐dependent depletion (CADD) scores^[^ ^41^ ^]^ was used to pinpoint deleterious variants (CADD score > 12.37),^[^ ^120^ ^]^ and RegulomeDB (RDB) scores^[^ ^42^ ^]^ was used to identify regulatory variants (RDB score < 4). In the ANNOVAR enrichment analysis, enrichment value was computed as (proportion of variants with a consequence) / (proportion of all available variants in the reference panel with the same consequence). The reference panel was the EAS‐LD reference from the 1000 Genomes Phase 3. Fisher's exact test was utilized to determine the significance of functional enrichments (*P* < 0.05/9/4 = 0.0014, corrected for nine consequences and four exposure categories). The overlap between genetic variants with significant interaction and expression quantitative trait loci (eQTL, *P* < 5 × 10^−8^) of brain tissues from a multi‐ancestry eQTL meta‐analysis (*n* = 3,154, including 474 non‐European individuals) were also examined.^[^ ^43^ ^]^ The meta‐analysis used the data from the dorsolateral prefrontal cortex (PsychENCODE and ROSMAP) and 13 brain regions (GTEx).

### Gene‐Based Interactions Analyses and Enrichment Analyses

The variants included in the GWEIS summary statistics were mapped to 18233 protein‐coding genes based on location. Gene‐based association analyses was then conducted to identify the genes with G × E interaction on brain IDPs (*P* < 0.05/18233 = 2.74 × 10^− 6^) based on the GWEIS summary data and the EAS‐LD reference from 1000 Genomes Project Phase3 using Multivariate Analysis of Genomic Annotation (MAGMA) v1.10.^[^ ^46^ ^]^ The “snp‐wise = mean” option was used, and the gene window was set as 35 kb upstream and 10 kb downstream to include regulatory elements. Based on the genes with G × E interactions on brain IDPs identified by MAGMA, protein–protein interaction (PPI) and enrichment analyses of gene ontology (GO) biological process (*q* < 0.05, BH‐FDR corrected) were also conducted for genes interacting with each exposure category using STRING 12.0.^[^ ^47^ ^]^

### Spatial Distribution of G × E Interactions in the Exposome and Phenome

Based on the number of verified G × E interactions for each exposure category, the interaction density of each exposure category was defined as the number of interactions of all exposures in the given category divided by the number of exposures of this category. The interaction density of each IDP category was defined using the same strategy. The spatial distribution of verified G × E interactions in the brain was summarized in terms of IDP categories.

### Estimating the Main Effects of Genetic Variants and Environmental Exposures

For each verified G × E interaction, it was tested whether the variant and the exposure also showed significant main effects. As the statistical significance of these main effects in interaction models (Equation 1) depends on the arbitrary coding of the variables,^[^ ^121^ ^]^ separate analyses were performed for estimating genetic (Equation 3) and environmental (Equation 4) main effects.

(3) $$y=\mu +\beta_{G}x_{G}+\beta_{C}x_{C}+\varepsilon$$

(4) $$y=\mu +\beta_{E}x_{E}+\beta_{C}x_{C}+\varepsilon$$

where *β*_*G*_ is the main effect of a given variant (*x_G_*), *β*_*E*_ is the main effect of a given exposure (*x_E_*), and *x_C_* is the same set of nuisance variables as in Equation (1). Multiple tests were corrected using the Bonferroni method (*P* < 0.05/the number of tests).

For each G × E interaction, *post‐hoc* analyses was also performed to calculate the environmental effect on the IDP in each genotype subgroup based on Equation (4). As the imputed genotype data were continuous variables ranging from 0 to 2, hard‐called thresholds were used to define the genotype subgroups (variant dosage: 0–0.2 for group 1; 0.8–1.2 for group 2; and 1.8–2.0 for group 3).^[^ ^122^ ^]^ If a variant of a participant with dosage out of the three ranges, the participant was defined with missing genotype for the variant. In Equation (4), there were 15 variables, including the intercept term, exposure, the first ten PCs, sex, age, and imaging covariate (e.g., TIV). As sample sizes of all subgroups should be two times larger than the number of variables for adequate estimation in linear regression analyses,^[^ ^123^ ^]^ the *post‐hoc* analyses were conducted when all genotype subgroups contained 30 or more participants. If participants in the genotype subgroup with two minor alleles was less than 30, this subgroup and the subgroup with the heterozygous genotype were combined.

### Estimating IDP Variances Explained by Main and Interaction Effects

Eta‐squared (η^2^) was used to estimate the effect sizes of the G × E interaction effects, genetic main effects, and environmental main effects based on the variances obtained from Equations (1), (3) and (4), respectively. Eta‐squared describes the ratio of the variance explained by the specific variable to the total variance:

(5) $$\eta^{2}=\frac{Var_{effect}}{Var_{T}}$$

where *Var_effect_* is the sum of squares explained by the environmental main effect, genetic main effect, or G × E interaction, and *Var_T_* is the total sum of squares in the full model.^[^ ^124^ ^]^ Wilcoxon sum rank test was used to compare the Eta‐squared difference between genetic or environmental main effects and G × E interactions.

### Identifying Sensitive Periods of G × E Interactions

For each verified G × E interaction involving time‐varying exposures, it was investigated in which periods the G × E interaction was significant. A distributed lag model (DLM) was applied to identify the sensitive periods of G × E interactions using the R package dlnm.^[^ ^53^ ^]^ DLM can evaluate the dependency between one longitudinal exposure and an outcome lagged in time. A bi‐dimensional structure was defined to estimate both the linear interaction‐response relationship and nonlinear lag‐response relationship across lags.^[^ ^53^ ^]^ Specifically, the DLM was built as the following equation:

(6) $$y=\mu +\sum_{j=1}^{18}\left[\alpha_{j}E_{j}\right]+\sum_{j=1}^{18}\left[\gamma_{j}I_{j}\right]+\beta_{G}G+\beta_{C}C+\varepsilon$$

Here, *E_j_* is the exposure at age *j* and *I_j_* = *E_j_* × *G* is the G × E interaction term at age *j*. *β*_*G*_ and *β*_*C*_ denote the effects of genetic variants and covariates, respectively. *α*_*j*_ and *γ*_*j*_ were the lag effects of exposure‐response and interaction‐response associations at age *j*, respectively. The DLM model can simultaneously integrate environmental data at all time points and estimate the G × E interaction effects at a given time point while controlling for the interactions at other time points. The smooth function of the lag‐response relationship was modeled using a natural cubic spline with three degrees of freedom. The knots were set at equally spaced values in the original lag scale (age 1 to 18 years with an interval of one year). A sensitive period was defined as a period during which the estimated pointwise 95% confidence intervals (CI) did not include zero.

In addition to the identification of the sensitive periods for each G × E interaction, the frequency of each year of age that was identified as a sensitive period across all the G × E interactions was also assessed. As population count, PM_2.5_, and NO_2_ showed large proportions of data missing (37.99%, 27.18%, and 26.00%; Supporting Information), only a few participants had complete data for the first 18 years. Several years of age had to remove in DLM analyses for these exposures to increase the sample size. Specifically, DLM analyses was performed at sample size > 500 for the three exposures. G × E interactions for these exposures were not included in the calculation of sensitive period frequency.

### Interactions Between Polygenic Risk Scores (PRSs) and Exposures

To identify interactions between PRSs for neuropsychiatric disorders and exposures on brain IDPs, EAS‐GWAS summary data of seven major neuropsychiatric disorders were included: anxiety (*n* = 5083),^[^ ^54^ ^]^ Alzheimer's disease (*n* = 8036),^[^ ^55^ ^]^ bipolar disorder (*n* = 15 18 80),^[^ ^56^ ^]^ major depressive disorder (*n* = 19 45 48),^[^ ^57^ ^]^ Parkinson's disease (*n* = 31575),^[^ ^58^ ^]^ schizophrenia (*n* = 30761),^[^ ^59^ ^]^ and stroke (*n* = 26 46 55).^[^ ^60^ ^]^ PRS scores for CHIMGEN participants were calculated using PRS‐CS^[^ ^61^ ^]^ and PLINK. Significant interactions were identified between seven PRS scores and 41 environmental exposures on 598 brain IDPs (*P* < 0.05/7/41/598 = 2.91 × 10^−7^, Bonferroni corrected).

## Conflict of Interest

The authors declare no conflict of interest.

## Author Contributions

S.W., M.W., P.Z. contributed equally to this work. C.Y., W.Q., M.L. and S.W. designed the study and wrote the manuscript. S.W. analyzed the data. C.Y., M.W., L.Y. supervised this work. P.Z., L.Y., J.C., L.Z., W.Z., S.Q., Z.G., G.C., Y.Y., W.L., X.‐N.Z., H.Z., B.G., X.X., T.H., Z.Y., Q.Z., F.L., Q.X., J.X., J.F., N.L., Y.J., J.T., L.G., M.L., X.X., X.L., W.L., C.W., W.W., D.S., S.L., Z.Y., F.C., J.Z., W.S., Y.M., D.W., J.‐H.G., Y.Y., K.X., J.X., B.Z., X.Z., and Z.Y. acquired the data. All authors critically reviewed the manuscript.

## Supporting information



Supporting Information

Supplemental Table 1‐28

## Data Availability

All GWEIS summary statistics based on the 7,084 CHIMGEN participants are freely available online (https://www.synapse.org/Synapse:syn71779380). We made use of publicly available software, tools and algorithms. Software used includes the boundary‐based registration (BBR) algorithm in FSL version 5.0.10 (https://fsl.fmrib.ox.ac.uk/fsl/docs/#/), BGENIE version 1.3 (https://jmarchini.org/bgenie/), CAT12 r1364 (http://dbm.neuro.uni‐jena.de/cat), ComBat harmonization (https://github.com/precision‐medicine‐um/ComBatHarmonization), the Diffeomorphic Anatomical Registration Through Exponentiated Lie Algebra (DARTEL) algorithm in SPM12 r7771 (https://www.fil.ion.ucl.ac.uk/spm/software/spm12/), DPARBI v3.0 (http://rfmri.org/DPABI), FUMA version 1.5.2 (https://fuma.ctglab.nl/), FreeSurfer version 6.0.0 (http://surfer.nmr.mgh.harvard.edu/), FSL version 5.0.10 (http://www.fmrib.ox.ac.uk/fsl), FSLNets in FSL version 5.0.10 (https://fsl.fmrib.ox.ac.uk/fsl/docs/#/other/other_software), GEE (https://earthengine.google.com/), ICA‐AROMA in FSL version 5.0.10 (https://fsl.fmrib.ox.ac.uk/fsl/docs/#/other/other_software), MAGMA v1.10 (https://cncr.nl/research/magma/), MELODIC in FSL version 5.0.10 (https://fsl.fmrib.ox.ac.uk/fsl/docs/#/resting_state/melodic), PLINK version 2.0.0a (https://www.cog‐genomics.org/plink/2.0/), PRS‐CS (https://github.com/getian107/PRScs), QCTOOL version 2.0.8 (https://www.well.ox.ac.uk/~gav/qctool_v2/), R package dlnm version 2.4.7 (https://github.com/gasparrini/dlnm), SEDAC (https://sedac.ciesin.columbia.edu/), SPM12 r7771 (https://www.fil.ion.ucl.ac.uk/spm/software/spm12/), STRING 12.0 (https://cn.string‐db.org/), and StructLMM (https://github.com/limix/struct‐lmm).

## References

[1] E. S. Finn , X. Shen , D. Scheinost , M. D. Rosenberg , J. Huang , M. M. Chun , X. Papademetris , R. T. Constable , Nat. Neurosci. 2015, 18, 1664. 26457551 10.1038/nn.4135 PMC5008686

[2] S. Mueller , D. Wang , M. D. Fox , B. T. T Yeo , J. Sepulcre , M. R. Sabuncu , R. Shafee , J. Lu , H. Liu , Neuron 2013, 77, 586. 23395382 10.1016/j.neuron.2012.12.028 PMC3746075

[3] K. Zilles , K. Amunts , Trends Cognit. Sci. 2013, 17, 153. 23507449 10.1016/j.tics.2013.02.003

[4] M. Schurz , L. Q. Uddin , P. Kanske , C. Lamm , J. Sallet , B. C. Bernhardt , R. B. Mars , D. Bzdok , Cerebral Cortex 2021, 31, 4612. 33982758 10.1093/cercor/bhab109 PMC8408465

[5] S. Genon , S. B. Eickhoff , S. Kharabian , Nat. Rev. Neurosci. 2022, 23, 307. 35365814 10.1038/s41583-022-00584-7

[6] Y. J. Woo , P. Roussos , V. Haroutunian , P. Katsel , S. Gandy , E. E. Schadt , J. Zhu , BMC Med. 2020, 18, 23. 32024511 10.1186/s12916-019-1488-1 PMC7003435

[7] M. Filippi , W. Brück , D. Chard , F. Fazekas , J. J. G. Geurts , C. Enzinger , S. Hametner , T. Kuhlmann , P. Preziosa , À. Rovira , K. Schmierer , C. Stadelmann , M. A. Rocca , Lancet. Neurol. 2019, 18, 198. 30663609 10.1016/S1474-4422(18)30451-4

[8] L.‐B. Cui , X.‐Y. Wang , Y.‐F. Fu , X.‐F. Liu , Y. Wei , S.‐W. Zhao , Y.‐W. Gu , J.‐W. Fan , W.‐J. Wu , H. Gong , B. D. Lin , H. Yin , F. Guan , X. Chang , BMC Med. 2023, 21, 250. 37424013 10.1186/s12916-023-02963-y PMC10332052

[9] C. G. Yan , X. Chen , L. Li , F. X. Castellanos , T. J. Bai , Q‐J Bo , J Cao , G.‐M. Chen , N.‐X. Chen , W. Chen , C. Cheng , Y.‐Q. Cheng , X.‐L. Cui , J. Duan , Y.‐R. Fang , Q.‐Y. Gong , W.‐B. Guo , Z.‐H. Hou , L. Hu , L. Kuang , F. Li , K.‐M. Li , T. Li , Y.‐S. Liu , Z.‐N. Liu , Y.‐C. Long , Q.‐H. Luo , H.‐Q. Meng , D.‐H. Peng , H.‐T. Qiu , et al., Proc Natl Acad Sci U S A. 2019, 116, 9078. 30979801 10.1073/pnas.1900390116 PMC6500168

[10] L. T. Elliott , K. Sharp , F. Alfaro‐Almagro , S. Shi , K. L. Miller , G. Douaud , J. Marchini , S. M. Smith , Nature 2018, 562, 210. 30305740 10.1038/s41586-018-0571-7 PMC6786974

[11] P. M. Thompson , T. D. Cannon , K. L. Narr , T. van Erp , V.‐P. Poutanen , M. Huttunen , J. Lönnqvist , C.‐G. Standertskjöld‐Nordenstam , J. Kaprio , M. Khaledy , R. Dail , C. I. Zoumalan , A. W. Toga , Nat. Neurosci. 2001, 4, 1253. 11694885 10.1038/nn758

[12] B. S. McEwen , Proc Natl Acad Sci U S A 2012, 109, 17180. 23045648 10.1073/pnas.1121254109 PMC3477378

[13] F. Liu , J. Xu , L. Guo , W. Qin , M. Liang , G. Schumann , C. Yu , Mol. Psychiatry 2023, 28, 17. 35790874 10.1038/s41380-022-01669-6

[14] A. Mechelli , C. Price , K. J. Friston , J. Ashburner , Curr. Med. Imaging Rev. 2005, 1, 105.

[15] A. M. Winkler , P. Kochunov , J. Blangero , L. Almasy , K. Zilles , P. T. Fox , R. Duggirala , D. C. Glahn , Neuroimage 2010, 53, 1135. 20006715 10.1016/j.neuroimage.2009.12.028 PMC2891595

[16] C. Lebel , S. Deoni , Neuroimage 2018, 182, 207. 29305910 10.1016/j.neuroimage.2017.12.097 PMC6030512

[17] B. Zhao , T. Li , S. M. Smith , D. Xiong , X. Wang , Y. Yang , T. Luo , Z. Zhu , Y. Shan , N. Matoba , Q. Sun , Y. Yang , M. E. Hauberg , J. Bendl , J. F. Fullard , P. Roussos , W. Lin , Y. Li , J. L. Stein , H. Zhu , Nat. Genet. 2022, 54, 508. 35393594 10.1038/s41588-022-01039-6 PMC11987081

[18] L. Jiang , X. N. Zuo , The Neuroscientist 2016, 22, 486. 26170004 10.1177/1073858415595004 PMC5021216

[19] S. M. Smith , G. Douaud , W. Chen , T. Hanayik , F. Alfaro‐Almagro , K. Sharp , L. T. Elliott , Nat. Neurosci. 2021, 24, 737. 33875891 10.1038/s41593-021-00826-4 PMC7610742

[20] B. Zhao , T. Li , Y. Yang , X. Wang , T. Luo , Y. Shan , Z. Zhu , D. Xiong , M. E. Hauberg , J. Bendl , J. F. Fullard , P. Roussos , Y. Li , J. L. Stein , H. Zhu , Science 2021, 372, abf3736. 10.1126/science.abf3736 PMC8370718 34140357

[21] K. L. Grasby , N. Jahanshad , J. N. Painter , L. Colodro‐Conde , J. Bralten , D. P. Hibar , P. A. Lind , F. Pizzagalli , C. R. K. Ching , M. A. Mahon , N. Shatokhina , L. C. P. Zsembik , S. I. Thomopoulos , A. H. Zhu , L. T. Strike , I. Agartz , S. Alhusaini , M. A. A. Almeida , D. Alnæs , I. K. Amlien , M. Andersson , T. Ard , N. J. Armstrong , A. Ashley‐Koch , J. R. Atkins , M. Bernard , R. M. Brouwer , E. E. L. Buimer , R. Bülow , C. Bürger , et al., Science 2020, 367, aay6690.

[22] C. Makowski , D. van der Meer , W. Dong , H. Wang , Y. Wu , J. Zou , C. Liu , S. B. Rosenthal , D. J. Hagler , C. C. Fan , W. S. Kremen , O. A. Andreassen , T. L. Jernigan , A. M. Dale , K. Zhang , P. M. Visscher , J. Yang , C.‐H. Chen , Science 2022, 375, 522. 35113692 10.1126/science.abe8457 PMC9469470

[23] M. Guxens , M. J. Lubczyńska , R. L. Muetzel , A. Dalmau‐Bueno , V. W. V. Jaddoe , G. Hoek , A. V. D. Lugt , F. C. Verhulst , T. White , B. Brunekreef , H. Tiemeier , H. E. Marroun , Biol. Psychiatry 2018, 84, 295. 29530279 10.1016/j.biopsych.2018.01.016

[24] J. Xu , X. Liu , Q. Li , R. Goldblatt , W. Qin , F. Liu , C. Chu , Q. Luo , A. Ing , L. Guo , N. Liu , H. Liu , C. Huang , J. Cheng , M. Wang , Z. Geng , W. Zhu , B. Zhang , W. Liao , S. Qiu , H. Zhang , X. Xu , Y. Yu , B. Gao , T. Han , G. Cui , F. Chen , J. Xian , J. Li , J. Zhang , et al., Nat. Human Behav. 2022, 6, 279. 34711977 10.1038/s41562-021-01204-7

[25] H. Tost , M. Reichert , U. Braun , I. Reinhard , R. Peters , S. Lautenbach , A. Hoell , E. Schwarz , U. Ebner‐Priemer , A. Zipf , A. Meyer‐Lindenberg , Nat. Neurosci. 2019, 22, 1389. 31358990 10.1038/s41593-019-0451-y

[26] K. G. Noble , S. M. Houston , N. H. Brito , H. Bartsch , E. Kan , J. M. Kuperman , N. Akshoomoff , D. G. Amaral , C. S. Bloss , O. Libiger , N. J. Schork , S. S. Murray , B. J. Casey , L. Chang , T. M. Ernst , J. A. Frazier , J. R. Gruen , D. N. Kennedy , P. Van Zijl , S. Mostofsky , W. E. Kaufmann , T. Kenet , A. M. Dale , T. L. Jernigan , E. R. Sowell , Nat. Neurosci. 2015, 18, 773. 25821911 10.1038/nn.3983 PMC4414816

[27] M. Yu , K. A. Linn , R. T. Shinohara , D. J. Oathes , P. A. Cook , R. Duprat , T. M. Moore , M. A. Oquendo , M. L. Phillips , M. McInnis , M. Fava , M. H. Trivedi , P. McGrath , R. Parsey , M. M. Weissman , Y. I. Sheline , Proc. Natl. Acad. Sci. USA 2019, 116, 8582. 30962366 10.1073/pnas.1900801116 PMC6486762

[28] N. Bittner , C. Jockwitz , T. W. Mühleisen , F. Hoffstaedter , S. B. Eickhoff , S. Moebus , U. J. Bayen , S. Cichon , K. Zilles , K. Amunts , S. Caspers , Nat. Commun. 2019, 10, 621. 30728360 10.1038/s41467-019-08500-x PMC6365564

[29] M. Kundakovic , K. Gudsnuk , J. B. Herbstman , D. Tang , F. P. Perera , F. A. Champagne , Proc. Natl. Acad. Sci. USA 2015, 112, 6807. 25385582 10.1073/pnas.1408355111 PMC4460453

[30] N. Kuzumaki , D. Ikegami , R. Tamura , N. Hareyama , S. Imai , M. Narita , K. Torigoe , K. Niikura , H. Takeshima , T. Ando , K. Igarashi , J. Kanno , T. Ushijima , T. Suzuki , M. Narita , Hippocampus 2011, 21, 127. 20232397 10.1002/hipo.20775

[31] J. S. Richards , A. Arias Vásquez , B. Franke , P. J. Hoekstra , D. J. Heslenfeld , J. Oosterlaan , S. V. Faraone , J. K. Buitelaar , C. A. Hartman , PLoS One 2016, 11, 0155755. 10.1371/journal.pone.0155755 PMC4878752 27218681

[32] H. Schneider‐Hassloff , B. Straube , A. Jansen , B. Nuscheler , G. Wemken , S. H. Witt , M. Rietschel , T. Kircher , Neuroimage 2016, 134, 671. 27109357 10.1016/j.neuroimage.2016.04.009

[33] C. P. Zeni , B. Mwangi , B. Cao , K. M. Hasan , C. Walss‐Bass , G. Zunta‐Soares , J. C. Soares , J. Affect. Disord 2016, 189, 94. 26432032 10.1016/j.jad.2015.09.031 PMC4733573

[34] Q. Xu , L. Guo , J. Cheng , M. Wang , Z. Geng , W. Zhu , B. Zhang , W. Liao , S. Qiu , H. Zhang , X. Xu , Y. Yu , B. Gao , T. Han , Z. Yao , G. Cui , F. Liu , W. Qin , Q. Zhang , M. J. Li , M. Liang , F. Chen , J. Xian , J. Li , J. Zhang , X.‐N. Zuo , D. Wang , W. Shen , Y. Miao , F. Yuan , et al., Mol. Psychiatry 2020, 25, 517. 31827248 10.1038/s41380-019-0627-6 PMC7042768

[35] R. Moore , F. P. Casale , M. Jan Bonder , D. Horta , L. Franke , I. Barroso , O. Stegle , Nat. Genet. 2019, 51, 180. 30478441 10.1038/s41588-018-0271-0 PMC6354905

[36] C. C. Chang , C. C. Chow , L. C. Tellier , S. Vattikuti , S. M. Purcell , J. J. Lee , GigaScience 2015, 4, 7. 25722852 10.1186/s13742-015-0047-8 PMC4342193

[37] B. Devlin , K. Roeder , Biometrics 1999, 55, 997. 11315092 10.1111/j.0006-341x.1999.00997.x

[38] R. Shi , X. Chang , T. Banaschewski , G. J. Barker , A. L. W. Bokde , S. Desrivières , H. Flor , A. Grigis , H. Garavan , P. Gowland , A. Heinz , R. Brühl , J.‐L. Martinot , M.‐L. P. Martinot , E. Artiges , F. Nees , D. P. Orfanos , L. Poustka , S. Hohmann , N. Holz , M. N. Smolka , N. Vaidya , H. Walter , R. Whelan , G. Schumann , X. Lin , J. Feng , Sci. Adv. 2024, 10, adp3751. 10.1126/sciadv.adp3751 PMC11567010 39546599

[39] K. Watanabe , E. Taskesen , A. van Bochoven , D. Posthuma , Nat. Commun. 2017, 8, 1826. 29184056 10.1038/s41467-017-01261-5 PMC5705698

[40] K. Wang , M. Li , H. Hakonarson , Nucleic Acids Res. 2010, 38, 164. 10.1093/nar/gkq603 PMC2938201 20601685

[41] M. Kircher , D. M. Witten , P. Jain , B. J. O'Roak , G. M. Cooper , J. Shendure , Nat. Genet. 2014, 46, 310. 24487276 10.1038/ng.2892 PMC3992975

[42] A. P. Boyle , E. L. Hong , M. Hariharan , Y. Cheng , M. A. Schaub , M. Kasowski , K. J. Karczewski , J. Park , B. C. Hitz , S. Weng , J. M Cherry , M. Snyder , Genome Res. 2012, 22, 1790. 22955989 10.1101/gr.137323.112 PMC3431494

[43] B. Zeng , J. Bendl , R. Kosoy , J. F. Fullard , G. E. Hoffman , P. Roussos , Nat. Genet. 2022, 54, 161. 35058635 10.1038/s41588-021-00987-9 PMC8852232

[44] F. Darra , T. Lo Barco , R. Opri , E. Parrini , C. Bianchini , E. Fiorini , A. Simonati , B. Dalla Bernardina , G. Cantalupo , R. Guerrini , Neurol. Genet. 2021, 7, 593. 10.1212/NXG.0000000000000593 PMC8131096 34017911

[45] M. Kamate , T. Basavanagowda , Cerebellum 2024, 23, 1239. 37749428 10.1007/s12311-023-01606-5

[46] C. A. de Leeuw , J. M. Mooij , T. Heskes , D. Posthuma , PLoS Comput. Biol. 2015, 11, 1004219. 10.1371/journal.pcbi.1004219 PMC4401657 25885710

[47] D. Szklarczyk , R. Kirsch , M. Koutrouli , K. Nastou , F. Mehryary , R. Hachilif , A. L. Gable , T. Fang , N. T. Doncheva , S. Pyysalo , Nucleic Acids Res. 2023, 51, D638. 36370105 10.1093/nar/gkac1000 PMC9825434

[48] V. Sagar , L. K. Shanahan , C. M. Zelano , J. A. Gottfried , T. Kahnt , Nat. Neurosci. 2023, 26, 1595. 37620443 10.1038/s41593-023-01414-4 PMC10726579

[49] D. Fernandez‐Fernandez , H. Rosenbrock , K. S. Kroker , Synapse 2015, 69, 484. 26178667 10.1002/syn.21840

[50] R. Farmer , S. D. Burbano , N. S. Patel , A. Sarmiento , A. J. Smith , M. P. Kelly , Cellular Signall. 2020, 70, 109592. 10.1016/j.cellsig.2020.109592 PMC7153982 32119913

[51] A. F. Wright , A. D. Carothers , H. Campbell , Pharmacogenomics J. 2002, 2, 75. 12049178 10.1038/sj.tpj.6500085

[52] S. B. Manuck , J. M. McCaffery , Annu. Rev. Psychol. 2014, 65, 41. 24405358 10.1146/annurev-psych-010213-115100

[53] A. Gasparrini , J. Stat. Softw. 2011, 43, 1. PMC3191524 22003319

[54] E. Friligkou , S. Løkhammer , B. Cabrera‐Mendoza , J. Shen , J. He , G. Deiana , M. D. Zanoaga , Z. Asgel , A. Pilcher , L. Di Lascio , A. Makharashvili , D. Koller , D. S. Tylee , G. A. Pathak , R. Polimanti , Nat. Genet. 2024, 56, 2036. 39294497 10.1038/s41588-024-01908-2 PMC12139100

[55] D. Shigemizu , R. Mitsumori , S. Akiyama , A. Miyashita , T. Morizono , S. Higaki , Y. Asanomi , N. Hara , G. Tamiya , K. Kinoshita , T. Ikeuchi , S. Niida , K. Ozaki , Translat. Psych. 2021, 11, 151. 10.1038/s41398-021-01272-3 PMC7925686 33654092

[56] K. S. O'Connell , M. Koromina , T. van der Veen , T. Boltz , F. S. David , J. M. K. Yang , K.‐H. Lin , X. Wang , J. R. I. Coleman , B. L. Mitchell , C. C. McGrouther , A. V. Rangan , P. A. Lind , E. Koch , A. Harder , N. Parker , J. Bendl , K. Adorjan , E. Agerbo , D. Albani , S. Alemany , N. Alliey‐Rodriguez , T. D. Als , T. F. M. Andlauer , A. Antoniou , H. Ask , N. Bass , M. Bauer , E. C. Beins , T. B. Bigdeli , et al., Nature 2025, 639, 968.39843750

[57] O. Giannakopoulou , K. Lin , X. Meng , M.‐H. Su , P.‐H. Kuo , R. E. Peterson , S. Awasthi , A. Moscati , J. R. I. Coleman , N. Bass , I. Y. Millwood , Y. Chen , Z. Chen , H.‐C. Chen , M.‐L. Lu , M.‐C. Huang , C.‐H. Chen , E. A. Stahl , R. J. F. Loos , N. Mullins , R. J. Ursano , R. C. Kessler , M. B. Stein , S. Sen , L. J. Scott , M. Burmeister , Y. Fang , J. Tyrrell , Y. Jiang , C. Tian , et al., JAMA psychiatry 2021, 78, 1258. 34586374 10.1001/jamapsychiatry.2021.2099 PMC8482304

[58] J. N. Foo , E. G. Y. Chew , S. J. Chung , R. Peng , C. Blauwendraat , M. A. Nalls , K. Y. Mok , W. Satake , T. Toda , Y. Chao , L. C. S. Tan , M. Tandiono , M. M. Lian , E. Y. Ng , K‐M. Prakash , W.‐L. Au , W.‐Y. Meah , S. Q. Mok , A. A. Annuar , A. Y. Y. Chan , L. Chen , Y. Chen , B. S. Jeon , L. Jiang , J. L. Lim , J.‐J. Lin , C. Liu , C. Mao , V. Mok , Z. Pei , et al., JAMA neurology 2020, 77, 746. 32310270 10.1001/jamaneurol.2020.0428 PMC7171584

[59] V. Trubetskoy , A. F. Pardiñas , T. Qi , G. Panagiotaropoulou , S. Awasthi , T. B. Bigdeli , J. Bryois , C.‐Y. Chen , C. A. Dennison , L. S. Hall , M. Lam , K. Watanabe , O. Frei , T. Ge , J. C. Harwood , F. Koopmans , S. Magnusson , A. L. Richards , J. Sidorenko , Y. Wu , J. Zeng , J. Grove , M. Kim , Z. Li , G. Voloudakis , W. Zhang , M. Adams , I. Agartz , E. G. Atkinson , E. Agerbo , et al., Nature 2022, 604, 502. 35396580 10.1038/s41586-022-04434-5 PMC9392466

[60] A. Mishra , R. Malik , T. Hachiya , T. Jürgenson , S. Namba , D. C. Posner , F. K. Kamanu , M. Koido , Q. Le Grand , M. Shi , Y. He , M. K. Georgakis , I. Caro , K. Krebs , Y.‐C. Liaw , F. C. Vaura , K. Lin , B. S. Winsvold , V. Srinivasasainagendra , L. Parodi , H.‐J. Bae , G. Chauhan , M. R. Chong , L. Tomppo , R. Akinyemi , G. V. Roshchupkin , N. Habib , Y. H. Jee , J. Q. Thomassen , V. Abedi , et al., Nature 2022, 611, 115. 36180795 10.1038/s41586-022-05165-3 PMC9524349

[61] T. Ge , C. Y. Chen , Y. Ni , Y. A. Feng , J. W. Smoller , Nat. Commun. 2019, 10, 1776. 30992449 10.1038/s41467-019-09718-5 PMC6467998

[62] R. Sundaramoorthy , T. Owen‐Hughes , F1000Research 2020, 9, F1000. 10.12688/f1000research.21933.1 PMC7445562 32864100

[63] S. M. Navickas , K. A. Giles , K. H. Brettingham‐Moore , P. C. Taberlay , Oncogene 2023, 42, 2363. 37433987 10.1038/s41388-023-02773-9 PMC10374441

[64] E. T. Lim , M. Uddin , S. De Rubeis , Y. Chan , A. S. Kamumbu , X. Zhang , A. M. D'Gama , S. N. Kim , R. S. Hill , A. P. Goldberg , C. Poultney , N. J. Minshew , I. Kushima , B. Aleksic , N. Ozaki , M. Parellada , C. Arango , M. J. Penzol , A. Carracedo , A. Kolevzon , C. M. Hultman , L. A. Weiss , M. Fromer , A. G. Chiocchetti , C. M. Freitag , G. M. Church , S. W. Scherer , J. D. Buxbaum , C. A. Walsh , Nat. Neurosci. 2017, 20, 1217. 28714951 10.1038/nn.4598 PMC5672813

[65] F. Tessadori , K. Duran , K. Knapp , M. Fellner , S. Smithson , A. B. Meireles , M. W. Elting , Q. Waisfisz , A. O'Donnell‐Luria , C. Nowak , J. Douglas , A. Ronan , T. Brunet , U. Kotzaeridou , S. Svihovec , M. S. Saenz , I. Thiffault , F. D. Viso , P. Devine , S. Rego , J. Tenney , A. V. Haeringen , C. A. L. Ruivenkamp , S. Koene , S. P. Robertson , C. Deshpande , R. Pfundt , N. Verbeek , J. M. V. D. Kamp , J. M. M. Weiss , et al., Am. J. Hum. Genet. 2022, 109, 750. 35202563 10.1016/j.ajhg.2022.02.003 PMC9069069

[66] Md. R Rahman , T. Islam , B. Turanli , T. Zaman , H. Md. Faruquee , Md. M Rahman , Md. N. H Mollah , R. K. Nanda , K. Y. Arga , E. Gov , M. A. Moni , Comput. Biol. Chem. 2019, 78, 431. 30606694 10.1016/j.compbiolchem.2018.12.011

[67] P. Zeng , H. F. Su , C. Y. Ye , S. W. Qiu , Q. Tian , Front. Pharmacol. 2021, 12, 806984. 34975502 10.3389/fphar.2021.806984 PMC8715940

[68] Z. Ning , X. Zhong , Y. Wu , Y. Wang , D. Hu , K. Wang , M. Deng , Phytomedicine 2024, 123, 155215. 38039902 10.1016/j.phymed.2023.155215

[69] B. L. Santos‐Lobato , A. F. Vidal , A. Ribeiro‐Dos‐Santos , Cells 2021, 10, 1410. 34204164 10.3390/cells10061410 PMC8228551

[70] J. T. R. Keeney , A. M. Swomley , J. L. Harris , A. Fiorini , M. I. Mitov , M. Perluigi , R. Sultana , D. A Butterfield , Neurotoxicity Res. 2012, 22, 220. 10.1007/s12640-011-9287-2 22083458

[71] A. K. West , J. Hidalgo , D. Eddins , E. D. Levin , M. Aschner , Neurotoxicology 2008, 29, 489. 18313142 10.1016/j.neuro.2007.12.006 PMC2486367

[72] L.‐C. Chen , P. Maciejczyk , D. Thurston , in Metals and Air Pollution, Academic Press, London, 2022, pp. 137–182.

[73] E. A. Crone , E. A. Konijn , Nat. Commun. 2018, 9, 588. 29467362 10.1038/s41467-018-03126-x PMC5821838

[74] M. Yuan , S. J. Cross , S. E. Loughlin , F. M. Leslie , J. Physiol. 2015, 593, 3397. 26018031 10.1113/JP270492 PMC4560573

[75] M. M. Herting , X. Chu , Birth Defects Res. 2017, 109, 1672. 29251839 10.1002/bdr2.1178 PMC5973814

[76] L. P. Spear , Nat. Rev. Neurosci. 2018, 19, 197. 29467469 10.1038/nrn.2018.10

[77] S. Goksan , F. Argyri , J. D. Clayden , F1000Res. 2020, 9, 370. 32528666 10.12688/f1000research.23216.1 PMC7262573

[78] A. E. Margolis , S. Banker , D. Pagliaccio , E. De Water , P. Curtin , A. Bonilla , J. B. Herbstman , R. Whyatt , R. Bansal , A. Sjödin , M. P. Milham , B. S. Peterson , P. Factor‐Litvak , M. K. Horton , Environm. Int. 2020, 134, 105212. 10.1016/j.envint.2019.105212 PMC7048018 31743804

[79] D. Fuhrmann , L. J. Knoll , S.‐J. Blakemore , Trends Cognit. Sci. 2015, 19, 558. 26419496 10.1016/j.tics.2015.07.008

[80] T. T. Brown , T. L. Jernigan , Neuropsychol. Rev. 2012, 22, 313. 23007644 10.1007/s11065-012-9214-1 PMC3511633

[81] J. N. Giedd , J. Blumenthal , N. O. Jeffries , F. X. Castellanos , H. Liu , A. Zijdenbos , T. S. Paus , A. C. Evans , J. L. Rapoport , Nat. Neurosci. 1999, 2, 861. 10491603 10.1038/13158

[82] G. Tang , K. Gudsnuk , S.‐H. Kuo , M. L. Cotrina , G. Rosoklija , A. Sosunov , M. S. Sonders , E. Kanter , C. Castagna , A. Yamamoto , Z. Yue , O. Arancio , B. S. Peterson , F. Champagne , A. J. Dwork , J. Goldman , D. Sulzer , Neuron 2014, 83, 1131. 25155956 10.1016/j.neuron.2014.07.040 PMC4159743

[83] S. M. Kolk , P. Rakic , Neuropsychopharmacology 2022, 47, 41. 34645980 10.1038/s41386-021-01137-9 PMC8511863

[84] L. D. Selemon , Translat. Psych. 2013, 3, 238.

[85] K. Konrad , C. Firk , P. J. Uhlhaas , Deutsches Arzteblatt Int. 2013, 110, 425. 10.3238/arztebl.2013.0425 PMC3705203 23840287

[86] B. J. Ellis , M. A. Sheridan , J. Belsky , K. A. McLaughlin , Developm. Psychopathol. 2022, 34, 447. 10.1017/S0954579421001838 35285791

[87] E. Harstad , E. Hanson , S. J. Brewster , R. DePillis , A. L. Milliken , G. Aberbach , G. Sideridis , W. J. Barbaresi , JAMA Pediatr. 2023, 177, 1197. 37782510 10.1001/jamapediatrics.2023.4003 PMC10546296

[88] A. Thapar , M. Cooper , Lancet 2016, 387, 1240. 26386541 10.1016/S0140-6736(15)00238-X

[89] J. van Os , S. S Kapur , Lancet 2009, 374, 635. 19700006 10.1016/S0140-6736(09)60995-8

[90] K. Georgiades , P. M. Lewinsohn , S. M. Monroe , J. R. Seeley , J. American Acad. Child Adolesc. Psych. 2006, 45, 936. 10.1097/01.chi.0000223313.25536.47 16865036

[91] E. Hannon , S. M. Anselimus , N. Bardikoff , B. Bulc , S. Germann , P. P. Gonsalves , G. J. Melendez‐Torres , L. Ospina‐Pinillos , M. Sinha , M. Wanjiru , Lancet 2023, 403, 708. 37827186 10.1016/S0140-6736(23)02238-9

[92] A. J. Fett , I. L. J. Lemmers‐Jansen , L. Krabbendam , Curr. Opin. Psychiatry 2019, 32, 232. 30724751 10.1097/YCO.0000000000000486 PMC6493678

[93] Z. Romeo , M. Biondi , L. Oltedal , C. Spironelli , F. Craig , Depression and Anxiety 2024, 2024, 1. 10.1155/2024/6673522 PMC11919126 40226746

[94] A. M. Shepherd , S. L. Matheson , K. R. Laurens , V. J. Carr , M. J. Green , Biol. Psychiatry 2012, 72, 775. 22621997 10.1016/j.biopsych.2012.04.020

[95] M. Hoogman , J. Bralten , D. P. Hibar , M. Mennes , M. P. Zwiers , L. S. J. Schweren , K. J. E. V. Hulzen , S. E. Medland , E. Shumskaya , N. Jahanshad , P. D. Zeeuw , E. Szekely , G. Sudre , T. Wolfers , A. M. H. Onnink , J. T. Dammers , J. C. Mostert , Y. Vives‐Gilabert , G. Kohls , E. Oberwelland , J. Seitz , M. Schulte‐Rüther , S. Ambrosino , A. E. Doyle , M. F. Høvik , M. Dramsdahl , L. Tamm , T. G. M. V. Erp , A. Dale , A. Schork , et al., Psychiatry 2017, 4, 310.28219628

[96] M. Aas , T. Ueland , T. V. Lagerberg , I. Melle , S. R. Aminoff , M. C. Hoegh , S. H. Lunding , J. F. Laskemoen , N. E. Steen , O. A. Andreassen , Psych. Res. 2023, 320, 115045. 10.1016/j.psychres.2022.115045 36621206

[97] B. Giles‐Corti , A. Vernez‐Moudon , R. Reis , G. Turrell , A. L. Dannenberg , H. Badland , S. Foster , M. Lowe , J. F. Sallis , M. Stevenson , N. Owen , Lancet 2016, 388, 2912. 27671668 10.1016/S0140-6736(16)30066-6

[98] C. Bycroft , C. Freeman , D. Petkova , G. Band , L. T. Elliott , K. Sharp , A. Motyer , D. Vukcevic , O. Delaneau , J. O'Connell , A. Cortes , S. Welsh , A. Young , M. Effingham , G. McVean , S. Leslie , N. Allen , P. Donnelly , J. Marchini , Nature 2018, 562, 203. 30305743 10.1038/s41586-018-0579-z PMC6786975

[99] T. L. Jernigan , S. A. Brown , G. J. Dowling , J. Res. Adolesc. 2018, 28, 154. 29460352 10.1111/jora.12374 PMC7477916

[100] J. Fu , Q. Zhang , J. Wang , M. Wang , B. Zhang , W. Zhu , S. Qiu , Z. Geng , G. Cui , Y. Yu , W. Liao , H. Zhang , B. Gao , X. Xu , T. Han , Z. Yao , W. Qin , F. Liu , M. Liang , S. Wang , Q. Xu , J. Xu , P. Zhang , W. Li , D. Shi , C. Wang , S. Lui , Z. Yan , F. Chen , J. Zhang , et al., Nat. Genet. 2024, 56, 1110. 38811844 10.1038/s41588-024-01766-y

[101] C. T. Lloyd , H. Chamberlain , D. Kerr , G. Yetman , L. Pistolesi , F. R. Stevens , A. E. Gaughan , J. J. Nieves , G. Hornby , K. MacManus , P. Sinha , M. Bondarenko , A. Sorichetta , A. J. Tatem , Big Earth Data 2019, 3, 108. 31565697 10.1080/20964471.2019.1625151 PMC6743742

[102] Y. Zha , J. Gao , S. Ni , Int. J. Remote Sens. 2003, 24, 583.

[103] S. Huang , L. Tang , J. P. Hupy , Y. Wang , G. Shao , J. For. Res. 2020, 32, 1.

[104] C. D. Elvidge , K. E. Baugh , E. A. Kihn , H. W. Kroehl , E. R. Davis , Photogramm. Eng. & Remote Sensing 1997, 63, 727.

[105] A. van Donkelaar , R. V. Martin , M. Brauer , N. C Hsu , R. A. Kahn , R. C. Levy , A. Lyapustin , A. M. Sayer , D. M. Winker , Environmental Science, Technol. 2016, 50, 3762. 10.1021/acs.est.5b05833 26953851

[106] J. A. Geddes , R. V. Martin , B. L. Boys , A. van Donkelaar , Environm. Health Perspect. 2016, 124, 281. 10.1289/ehp.1409567 PMC4786989 26241114

[107] W. M. Alley , J. Appl. Meteorol. Climatol. 1984, 23, 1100.

[108] J. T. Abatzoglou , S. Z. Dobrowski , S. A. Parks , K. C. Hegewisch , Scientific Data 2018, 5, 170191. 29313841 10.1038/sdata.2017.191 PMC5759372

[109] E. Becker , S. Saha , S. Moorthi , X. Wu , J. Wang , S. Nadiga , P. Tripp , D. Behringer , Y. ‐T. Hou , H. ‐Y. Chuang , M. Iredell , M. Ek , J. Meng , R. Yang , M. P. Mendez , H. V. D. Dool , Q. Zhang , W. Wang , M. Chen , Journal of Climate 2014, 27, 2185.

[110] Y. Xu , L. Yu , D. Peng , J. Zhao , Y. Cheng , X. Liu , W. Li , R. Meng , X. Xu , P. Gong , Sci. China Earth Sci. 2020, 63, 1390.

[111] L. Jiang , L. Yu , Glob. Ecol. Conserv. 2019, 20, 00789.

[112] D. P. Bernstein , T. Ahluvalia , D. Pogge , L. Handelsman , J. Am. Acad. Child Adolesc. Psychiatry 1997, 36, 340. 9055514 10.1097/00004583-199703000-00012

[113] N. Gorelick , M. Hancher , M. Dixon , S. Ilyushchenko , R. Moore , Remote Sens. Environm. 2017, 202, 18.

[114] L. Yu , Z. Du , X. Li , J. Gu , X. Li , L. Zhong , Duojiweise , H. Wu , Q. Zhao , X. Ma , J. Zheng , Y. Yang , W. Song , P. Wang , Z. Zhao , L. Liao , Y. Long , Y. Zhang , J. Peng , M. Shen , T. Li , Z. Sun , Y. Zhao , C. Wu , G. Lin , Y. Luo , D. Peng , J. Rem. Sens. 2022, 59, 1026.

[115] T. Calinski , J. Harabasz , Commun. Stat. ‐ Theory Methods 1974, 3, 1.

[116] J.‐P. Fortin , D. Parker , B. Tunç , T. Watanabe , M. A. Elliott , K. Ruparel , D. R. Roalf , T. D. Satterthwaite , R. C. Gur , R. E. Gur , R. T. Schultz , R. Verma , R. T. Shinohara , Neuroimage 2017, 161, 149. 28826946 10.1016/j.neuroimage.2017.08.047 PMC5736019

[117] M. Jenkinson , P. Bannister , M. Brady , S. Smith , Neuroimage 2002, 17, 825. 12377157 10.1016/s1053-8119(02)91132-8

[118] K. E. Westerman , T. D. Majarian , F. Giulianini , D.‐K. Jang , J. Miao , J. C. Florez , H. Chen , D. I. Chasman , M. S. Udler , A. K. Manning , J. B. Cole , Nat. Commun. 2022, 13, 3993. 35810165 10.1038/s41467-022-31625-5 PMC9271055

[119] R. F. Hillary , D. A. Gadd , Z. Kuncheva , T. Mangelis , T. Lin , K. Ferber , H. McLaughlin , H. Runz , E. Marshall , R. E. Marioni , C. N. Foley , B. B. Sun , Nat. Commun. 2024, 15, 7346. 39187491 10.1038/s41467-024-51744-5 PMC11347662

[120] L. M. Amendola , M. O. Dorschner , P. D. Robertson , J. S. Salama , R. Hart , B. H. Shirts , M. L. Murray , M. J. Tokita , C. J. Gallego , D. S. Kim , J. T. Bennett , D. R. Crosslin , J. Ranchalis , K. L. Jones , E. A. Rosenthal , E. R. Jarvik , A. Itsara , E. H. Turner , D. S. Herman , J. Schleit , A. Burt , S. M. Jamal , J. L. Abrudan , A. D. Johnson , L. K. Conlin , M. C. Dulik , A. Santani , D. R. Metterville , M. Kelly , A. K. M. Foreman , et al., Genome Res. 2015, 25, 305. 25637381 10.1101/gr.183483.114 PMC4352885

[121] A. F. Hayes , C. J. Glynn , M. E. Huge , Communicat. Meth. Measur. 2012, 6, 1.

[122] J. A. Collister , X. Liu , L. Clifton , Front. Genet. 2022, 13, 818574. 35251129 10.3389/fgene.2022.818574 PMC8894758

[123] P. C. Austin , E. W. Steyerberg , J Clin Epidemiol. 2015, 68, 627. 25704724 10.1016/j.jclinepi.2014.12.014

[124] L. P. Ly , D. J. Handelsman , Clin. Exp. Pharmacol. Physiol. 2002, 29, 39.

