# Supplementary material for: Genome‐Wide by Lifetime Environment Interaction Studies of Brain Imaging Phenotypes
Source: Adv Sci (Weinh). 2025 Nov 29;13(8):e00852. doi: 10.1002/advs.202500852 (PMC12884788; doi:10.1002/advs.202500852)
Supplement: Supplementary file 1 — Supporting Information [file ADVS-13-e00852-s001.docx]

Supplementary Materials for

**Genome-wide by lifetime environment interaction studies of brain imaging phenotypes**

**Authors:** Sijia Wang^1,#^, Meiyun Wang^2,3,#^, Peng Zhang^4,#^, Jingliang Cheng^5^, Longjiang Zhang^6^, Wenzhen Zhu^7^, Shijun Qiu^8^, Zuojun Geng^9^, Guangbin Cui^10^, Yongqiang Yu^11^, Weihua Liao^12,13,14^, Xi-Nian Zuo^15,16^, Hui Zhang^17^, Bo Gao^18,19^, Xiaojun Xu^20^, Tong Han^21^, Zhenwei Yao^22^, Quan Zhang^23^, Feng Liu^1^, Qiang Xu^1^, Jiayuan Xu^1^, Jilian Fu^1^, Nana Liu^1^, Yuan Ji^1^, Jie Tang^1^, Lining Guo^1^, Mengge Liu^1^, Xiaoxiao Xiao^24^, Xiaoxuan Liu^25^, Wei Li^4^, Caihong Wang^5^, Wei Wei^2^, Dapeng Shi^2^, Su Lui^26,27^, Zhihan Yan^28^, Feng Chen^29^, Jing Zhang^30,31^, Wen Shen^32^, Yanwei Miao^33^, Dawei Wang^34^, Jia-Hong Gao^35^, Yunjun Yang^36^, Kai Xu^37^, Junfang Xian^38^, Bing Zhang^39^, Xiaochu Zhang^40^, Zhaoxiang Ye^4^, Le Yu^25*^, Wen Qin^1*^, Meng Liang^24*^, Chunshui Yu^1,24*^ and the CHIMGEN Consortium

^#^These authors contributed equally to this work: Sijia Wang, Meiyun Wang, Peng Zhang

^*^Correspondence to:

Chunshui Yu, e-mail: chunshuiyu@tmu.edu.cn

Meng Liang, e-mail: liangmeng@tmu.edu.cn

Wen Qin, e-mail: qinwen@tmu.edu.cn

Le Yu, e-mail: leyu@tsinghua.edu.cn

**The PDF file includes:**

Supplementary Text

Figs. S1 to S27

References

**Other Supplementary Materials for this manuscript include the following:**

Tables S1 to S28

# Supplementary text

## Satellite-based environmental assessments

We asked each participant to recall his/her precise residential coordinates of the years from birth to the recruitment. If the participant moved in a year, the address with the longest living time was defined as the residential coordinate of that year. To minimize recall bias, the residential addresses of each participant was confirmed in a separate visit. If inconsistency was found between the two visits, we would ask the participant to confirm which one was correct. Finally, the annual residential addresses from birth to recruitment were obtained from 5,851 participants. As the age of Chinese Imaging Genetics (CHIMGEN) participants was ranged from 18 to 30 years[^1^](#_ENREF_1_1), we only acquired the environmental exposures from birth to the age of 18 to keep the consistency of the timespan of exposures across the participants.

Based on the residential coordinates of each participant, we obtained the annual data of external environmental exposures based on the satellite remote sensing data provided by the publicly available databases, such as the Google Earth Engine (GEE, https://earthengine.google.com/)[^2^](#_ENREF_1_2), the Social Economic Data and Applications Center (SEDAC, <https://sedac.ciesin.columbia.edu/>), and the annual China's land use/land cover dataset (CLUD-A)[^3^](#_ENREF_1_3)^,^[^4^](#_ENREF_1_4). These environmental exposures included 21 time-varying and three time-invariant exposures. The 21 time-varying exposures included the population count[^5^](#_ENREF_1_5), normalized difference built-up index (NDBI)[^6^](#_ENREF_1_6), normalized difference vegetation index (NDVI)[^7^](#_ENREF_1_7), nighttime lights (NL)[^8^](#_ENREF_1_8), particular matter 2.5 (PM_2.5_)[^9^](#_ENREF_1_9), nitrogen dioxide (NO_2_)[^10^](#_ENREF_1_10), Palmer drought severity index (PDSI)[^11^](#_ENREF_1_11), precipitation[^12^](#_ENREF_1_12), humidity[^13^](#_ENREF_1_13), surface pressure[^13^](#_ENREF_1_13), wind speed[^12^](#_ENREF_1_12), temperature[^13^](#_ENREF_1_13), temperature difference (TD), bare-land%[^3^](#_ENREF_3), cropland%[^3^](#_ENREF_3), grassland%[^3^](#_ENREF_3), forest%[^3^](#_ENREF_3), scrub-land%[^3^](#_ENREF_3), built-up%[^3^](#_ENREF_3), water body%[^3^](#_ENREF_3), and land use intensity (LUI)[^14^](#_ENREF_1_14). The three time-invariant exposures included the altitude, latitude, and longitude of the birthplace. Based on the individual’s residential coordinates of the calendar years of his/her first 18 years of age, we obtained the annual data of these external physical exposures for this participant. Data resource and description of each exposure were introduced as follows:

The annual data of NDVI and NDBI of each participant were downloaded from GEE (https://earthengine.google.com/). NDVI and NDBI were calculated based on the satellite images acquired by Landsat-5 Thematic Mapper (TM) sensor (1986-1999) (<https://developers.google.com/earth-engine/datasets/catalog/LANDSAT_LT05_C02_T1_L2>) and Landsat-7 Enhanced Thematic Mapper Plus (ETM+) sensor (2000-2019) (<https://developers.google.com/earth-engine/datasets/catalog/LANDSAT_LE07_C02_T1_L2>). With the near-infrared (NIR), shortwave infrared (SWIR), and red (RED) reflectance data of each satellite image, NDVI was calculated by (NIR ‒ RED) / (NIR + RED)[^7^](#_ENREF_1_7), while NDBI was computed by (SWIR ‒ NIR) / (SWIR + NIR)[^6^](#_ENREF_1_6). Both NDVI and NDBI values were ranged from -1 to 1, with higher values indicating a higher density of greenness[^7^](#_ENREF_1_7)^,^[^15^](#_ENREF_1_15) or built-up areas[^6^](#_ENREF_1_6)^,^[^15^](#_ENREF_1_15). The NDVI and NDBI data had a spatial resolution of 30 m, a temporal resolution of 16 days, and a timespan from January 1986 to December 2019.

The annual NL data for the first 18 years of age were also downloaded from GEE (<https://developers.google.com/earth-engine/datasets/catalog/NOAA_DMSP-OLS_NIGHTTIME_LIGHTS>) for each participant. NL captures the stable nighttime radiance from anthropogenic sources such as streetlights and exterior building lights, which is used to assess the intensity of local human economic activities[^16^](#_ENREF_1_16). NL was calculated from visible and near-infrared emission sources at night based on the satellite images from the defense meteorological program operational line-scan system (DMSP-OLS)[^8^](#_ENREF_1_8). NL data had a spatial resolution of 30 arc seconds (approximate 924 m), a temporal resolution of one year, and a timespan from January 1992 to December 2013. NL values were ranged from 0 to 63, with higher values indicating more intense nighttime lights.

The individual’s annual data of population count were downloaded from GEE (<https://developers.google.com/earth-engine/datasets/catalog/WorldPop_GP_100m_pop>). The global high-resolution population data are deposited at the world population (WorldPop) platform (<https://hub.worldpop.org/geodata/listing?id=29>)[^17^](#_ENREF_1_17), in which the population count was defined as the estimated number of people residing in a grid-cell (3 arc seconds, 92.4 m at the equator) and the annual population count data were available from 2000 to 2020[^5^](#_ENREF_1_5).

The annual data of PDSI, precipitation, and wind speed were obtained from GEE (<https://developers.google.com/earth-engine/datasets/catalog/IDAHO_EPSCOR_TERRACLIMATE>). TerraClimate (<http://www.climatologylab.org/terraclimate.html>)[^12^](#_ENREF_1_12) is a dataset of monthly climate data for global terrestrial surfaces with a spatial resolution of 4.625 km from 1958 onwards, which is deposited at GEE. PDSI is a standardized index with PDSI ≥ 4 indicating extremely wet weather and PDSI ≤ -4 indicating extremely drought weather[^11^](#_ENREF_1_11).

We also downloaded surface pressure, humidity, and temperature data from GEE (<https://developers.google.com/earth-engine/datasets/catalog/NOAA_CFSV2_FOR6H#bands>). These climate data were recorded by the National Centers for Environmental Prediction (NCEP) (<https://cfs.ncep.noaa.gov/>) climate forecast system (CFS, version 2)[^13^](#_ENREF_1_13). The CFSv2 dataset provided 6-hour climate data with a spatial resolution of 0.2 arc degrees (about 22.2 km) from 1979 to present. The surface pressure was measured at earth surface, while the humidity and temperature were measured at 2 m above the ground[^18^](#_ENREF_1_18). Based on the temperature data, we calculated the average temperature of each month. The temperature difference of each year was defined as the difference between the highest and lowest monthly temperatures in this year.

The annual ground-level PM_2.5_ concentration data were obtained from SEDAC (https://beta.sedac.ciesin.columbia.edu/data/set/sdei-global-annual-gwr-pm2-5-modis-misr-seawifs-aod). Global near-surface PM_2.5_ concentrations (ug/m^3^) were estimated based on the aerosol optical depth (AOD) data from multiple satellite algorithms including the National Aeronautics and Space Administration (NASA) Moderate Resolution Imaging Spectroradiometer (MODIS), Sea-Viewing Wide Field-of-View Sensor (SeaWiFS), and Multi-angle Imaging Spectroradiometer (MISR)[^9^](#_ENREF_1_9)^,^[^19^](#_ENREF_1_19). The PM_2.5_ data had a spatial resolution of 0.01 degree (about 1.1 km) and a timespan from 1998 to 2016[^19^](#_ENREF_1_19).

The annual ground-level NO_2_ concentrations were downloaded from SEDAC (<https://sedac.ciesin.columbia.edu/data/set/sdei-global-3-year-running-mean-no2-gome-sciamachy-gome2>). In SEDAC, the global ground-level NO_2_ concentration (parts per billion) data were provided by the Global 3-Year Running Mean Ground-Level Nitrogen Dioxide Grids, which were constructed based on the satellite retrievals (1996-2012) from Global Ozone Monitoring Experiment (GOME), Scanning Imaging Absorption Spectrometer for Atmospheric Chartography (SCIAMACHY), and Global Ozone Monitoring Experiment-2 (GOME-2)[^10^](#_ENREF_1_10)^,^[^20^](#_ENREF_1_20). For each calendar year, the mean NO_2_ level of three years centered at this year was defined as the NO_2_ level of this year. The annual NO_2_ data had a spatial resolution of six arc-minutes (0.1 degree, or approximately 11 km at the equator) and a timespan from 1997 to 2011.

The annual data of bare-land%, cropland%, grassland%, forest%, scrub-land%, built-up%, and water body% of each participant were extracted from the annual China's land use/land cover dataset (CLUD-A)[^3^](#_ENREF_1_3)^,^[^4^](#_ENREF_1_4), which was created based on satellite images of multiple resources and provided the information of land cover and land use in China with a spatial resolution of 30 m and a timespan from 1980 to 2015[^3^](#_ENREF_3). The bare-land%, cropland%, grassland%, forest%, scrub-land%, built-up%, and water body% were the percentage of certain type of land cover. According to a prior study[^14^](#_ENREF_1_14), we calculated LUI to reflect human activities based on the land cover data. LUI had the same spatial and temporal resolution as land cover%, and LUI values were ranged from 100 to 400, with higher values indicating more intense human activities[^14^](#_ENREF_1_14).

The latitude and longitude of birthplace of each participant were extracted from the WGS84 (World Geodetic System 1984) coordinates. The birthplace altitude data provided by the Shuttle Radar Topography Mission (SRTM) were obtained from GEE (<https://developers.google.cn/earth-engine/datasets/catalog/CGIAR_SRTM90_V4#description>). The SRTM digital elevation dataset was developed by NASA and provided high-quality elevation data nearly across the globe with a spatial resolution of 90 m[^21^](#_ENREF_1_21).

As the time-varying exposure data had different spatial and temporal resolutions, we resampled these data into a consistent spatial resolution of 1 km and a temporal resolution of one year. For the 5,851 participants with lifetime residential information, the timespan of the calendar years of their first 18 years of age was ranged from 1986 to 2018. Due to the availability of satellite data, the time-varying exposure data were missing for certain years of age in these participants. The complete exposure data of the participants should include 105,318 spatiotemporal points (5,851 participants × 18 years), based on which we defined the missing rate of each exposure as the percentage of its unavailable spatiotemporal points. For example, NL data were available from 1992 to 2013, and thus NL data of 1986-1991 and 2014-2018 were missing, resulting in 4,809 missing spatiotemporal points and a missing rate of 4.57% (4,809/105,318). In the 5,851 participants, there were no missing data for precipitation, PDSI, humidity, temperature, temperature difference, surface pressure, and wind speed; 0.01% missing data for NDBI and NDVI; 0.24% for bare-land%, cropland%, grassland%, forest%, scrub-land%, built-up%, water body% and LUI; 4.57% for NL; 26.00% for NO_2_; 27.18% for PM_2.5_; and 37.99% for population count. As the missing exposure data imputation with robust approaches such as the multiple imputation via chained equations (MICE) would generate multiple sets of exposure data and greatly increase the computation burden in genome-wide by environment interaction studies (GWEIS), we calculated the average exposure for each participant by averaging the available annual exposure data of the first 18 years of age. In these 5,851 participants, the mean available years of time-varying exposures was 17.16 years (with a standard deviation of 2.16 years). More details are shown in **Fig. S26**.

## Paper-based environmental assessments

The paper-based environmental questionnaires were used to assess other 17 exposures, including the seven binary variables (yes = 1 and no = 0) of four birth seasons, only child status, parental divorce and death, and ten continuous variables of the urbanicity score, education years, mother’s and father’s ages at birth, and total and five subscale scores of the childhood trauma questionnaire (CTQ)[^22^](#_ENREF_1_22) (**Table S2**). To assess the urbanicity score, we asked each participant to recall the residential addresses and duration of residence in the first 18 years of life, and classified these addresses into categories of rural, town, and city according to the definition of the National Bureau of Statistics of China. By assigning a score to each category (rural = 1, town = 2, and city = 3), we defined the urbanicity score of each participant as the summed score of the first 18 years of age. The urbanicity score was ranged from 18 to 54 with higher scores indicating higher urbanicity[^23^](#_ENREF_1_23). The CTQ consists of 28 items and 5 subscales: emotional abuse, physical abuse, sexual abuse, emotional neglect and physical neglect. The scores of each subscale range from 5 to 25 points, with a total score of 25-125 points, and higher scores correspond to more severe abuse. CTQ has been translated into Chinese and showed good reliability and validity[^24^](#_ENREF_1_24). To reduce the measurement errors, several questions were designed to validate the accuracy and correctness of answers from each participant. For instance, we asked the same or related question in different visits, and then checked the consistency of the answers.

## Brain MRI data acquisition and preprocessing

In the CHIMGEN study, brain MRI data were acquired by ten types of 3.0-Tesla MRI scanners and 12 sets of scanning parameters (**Tables S26-28**). The structural MRI data were used to calculate cortical thickness (CT), surface area (SA), and gray matter volume (GMV) to assess the macrostructural properties of gray matter. The diffusion MRI data were utilized to calculate fractional anisotropy (FA) of each voxel. FA reflects the directionality of diffusion of water molecules, which was used to assess the microstructural integrity of brain white matter (WM). The resting-state functional MRI (fMRI) data were used to calculate regional homogeneity (ReHo) of each gray matter (GM) voxel and to construct resting-state networks (RSNs). ReHo was used to assess the regional homogeneity of spontaneous neuronal activity. We calculated functional activity amplitude (Amp) within and functional connectivity (FC) between RSNs to evaluate the spontaneous neuronal activity within and the temporal coherence of spontaneous neuronal activity between RSNs, respectively. MRI data preprocessing and calculation of imaging-derived phenotypes (IDPs) were described in the following sections.

### Structural MRI data preprocessing for GMV-IDP calculation

We preprocessed structural MRI data using the computational anatomy toolbox (CAT 12, version r1364, <http://dbm.neuro.uni-jena.de/cat>) with the standard pipeline. After correcting for image inhomogeneity caused by B1-field bias, structural MR images were segmented into GM, WM, and cerebrospinal fluid (CSF) using an adaptive Maximum A Posterior (MAP) technique[^25^](#_ENREF_1_25). The tissue probability templates for GM, WM, and CSF in Montreal Neurological Institute (MNI) space were obtained from 6,000 CHIMGEN participants using the Diffeomorphic Anatomical Registration Through Exponentiated Lie Algebra (DARTEL)[^26^](#_ENREF_1_26) algorithm implemented in statistical parametric mapping (SPM12; <https://www.fil.ion.ucl.ac.uk/spm/software/spm12/>). We normalized the segmented GM images to the population-specific GM template using the DARTEL algorithm and resampled into a cubic voxel of 1.5 mm. Modulation was performed on the GM images to preserve absolute GMV. Based on the segmented GM, WM, and CSF images, we calculated the total intracranial (GM+WM+CSF), GM, and WM volumes for each participant. Based on the individual’s voxel-wise GMV map, we extracted the GMVs of 90 cerebral regions defined by the anatomical automatic labeling (AAL) atlas[^27^](#_ENREF_1_27) and 34 cerebellar regions defined by the spatially unbiased infra-tentorial (SUIT) template[^28^](#_ENREF_1_28) (<https://diedrichsenlab.org/imaging/propatlas.htm>).

### Structural MRI data preprocessing for cortical IDP calculation

The brain structural MRI data were preprocessed by the FreeSurfer v6.0.0 package (<http://surfer.nmr.mgh.harvard.edu/>) with the default pipeline. We separated the brain from non-brain tissues in structural MR images and applied intensity normalization before and after skull stripping to correct intensity non-uniformity due to variations in the sensitivity of reception coils and gradient-driven eddy currents. Based on intensity and neighbor constraints, tissue segmentation was conducted to obtain the subcortical structures and GM-WM boundary. After the construction of the WM and pial surfaces, we performed topology correction to repair defects and calculated the CT and SA of each vertex. Individual’s surfaces were inflated into a spherical space and normalized to the fsaverage template to obtain spherical normalization parameters. Here, we used the cerebral cortex parcellation scheme of the Desikan-Killiany-Tourville (DKT) atlas ([https://freesurfer.net/fswiki/CorticalParcellation)](https://freesurfer.net/fswiki/CorticalParcellation)17) , which divided the cerebral cortex into 62 distinct cortical regions. After converting the DKT surface atlas from standard to individual space using the inverse spherical normalization parameters, we extracted the CT and SA of the 62 cortical regions and the total SA and mean CT of the left or right hemisphere.

### Diffusion MRI data preprocessing and FA-IDP calculation

FSL 5.0.10 (<https://fsl.fmrib.ox.ac.uk/fsl/fslwiki/>) was used to preprocess the diffusion MRI data. The non-brain tissues were removed from the b = 0 images to generate a binary mask for tensor and metric calculation. The eddy_openmp program was used to evaluate and repair image displacement and signal dropout caused by head motion, and image distortion caused by eddy current. A linear least square algorithm was used to estimate diffusion tensor and calculate FA of each voxel from the tensor using the DTIFIT program. Two steps were used to estimate normalization parameters between individual and MNI space. Individual b = 0 images were aligned to structural images using boundary-based registration (BBR). The BBR transformation matrix was then concatenated with the DARTEL deformation field from individual to MNI space generated in the structural MRI preprocessing. After normalizing individual FA image into the MNI space using the merged deformation field, the mean FA image of all individuals was created and “thinned” to generate a mean white matter skeleton to represent the centers of white matter tracts common to all individuals. The aligned FA image of each participant was then projected onto the mean white matter skeleton by filling the mean skeleton with FA values from the nearest tract center, which was achieved by searching perpendicular to the local skeleton structure for maximal value. From the FA skeleton map of each participant, we extracted the FA values of 48 white matter fiber tracts from the atlas of the Johns Hopkins University (JHU) white matter labels (<https://fsl.fmrib.ox.ac.uk/fsl/fslwiki/Atlases>).

### Resting-state fMRI data preprocessing and metric calculation

The resting-state fMRI data were preprocessed by calling functions from SPM 12 and DPARBI v3.0 (<http://rfmri.org/DPABI>). After discarding functional volumes acquired at about the first ten seconds, the remaining volumes were corrected for intra-volume temporal differences using sinc-interpolation and the inter-volume head motion was estimated and corrected by realigning each volume to mean volume using rigid-body transformation. We conducted independent component analysis based automatic removal of motion artifacts (ICA-AROMA)[^29^](#_ENREF_1_29), from which we identified and removed the independent components for motion artifacts. After removing non-brain tissues, the functional images were co-registered to the structural images using the BBR method. All co-registered functional volumes were normalized to the MNI space using the DARTEL deformation field derived from structural MRI data preprocessing and resampled to 3-mm voxels. After regressing out linear trend and fMRI signals from WM and CSF, a temporal band-pass filtering (0.01-0.08 Hz) was used to reduce low-frequency drift and high-frequency noise.

The ReHo of each voxel was defined as the Kendall’s coefficient concordance of the time series of this voxel with those of its nearest neighbors (26 voxels)[^30^](#_ENREF_1_30). We calculated ReHo values for all GM voxels to create a voxel-wise ReHo map for each participant. For standardization, ReHo of each voxel was subtracted from the mean and divided by the standard deviation (SD) of ReHo values of all GM voxels. From the voxel-wise ReHo map of each participant, we extracted the ReHo values of 90 cerebral regions defined by the AAL atlas[^27^](#_ENREF_1_27) and 34 cerebellar regions defined by the SUIT atlas[^28^](#_ENREF_1_28).

Based on the preprocessed resting-state fMRI data from all participants, group independent component analysis (group-ICA) was performed using FSL’s MELODIC (<https://fsl.fmrib.ox.ac.uk/fsl/fslwiki/MELODIC>). Of the 25 independent components (ICs) generated by group-ICA, the 18 non-artefactual ICs were defined as the RSNs **(Fig. S27).** For each participant, the spatial map and time series of each IC was obtained by mapping the IC spatial map into the participant’s fMRI time series using dual-regression (<https://fsl.fmrib.ox.ac.uk/fsl/fslwiki/DualRegression>). With the FSLNets toolbox (<http://fsl.fmrib.ox.ac.uk/fsl/fslwiki/FSLNets>), we regressed out the signals of the seven artefactual components from the 18 RSNs, and then calculated the Amp of each RSN and the FC between RSNs. Here, FC was defined as the Pearson’s correlation between RSNs, and Fisher’s r-to-z transformation was applied to improve normality. We extracted 18 Amp-IDPs and 153 FC-IDPs.

## Quality control (QC) of MRI data acquisition and processing

Since different scanners were applied to acquire MRI data, we implemented a series of QC procedures during the whole process of MRI data acquisition and processing.

### QC before scanning

**Scanner selection.** This study utilized only 3.0-Tesla scanners to ensure the quality of MRI data. MRI scanners were exclusively chosen from GE, Siemens, and Philips to minimize scanner-related variability. GE MR 750 scanners were preferred for their outstanding image quality and widespread use in China.

**Sequence selection.** Considering participant tolerance constraints and the popularity of imaging sequences across MRI scanners, we selected structural MRI, diffusion tensor imaging, and resting-state fMRI as essential sequences.

**Imaging parameters.** A team of researchers with extensive experience in developing protocols for MRI studies from Tianjin Medical University General Hospital (TMUGH) were responsible for creating imaging parameters for various MRI scanners. We then obtained MRI data from three volunteers using each scanner. The QC team evaluated the contrast, signal-to-noise ratio (SNR), homogeneity, and artefacts of these images, making iterative parameter adjustments until the image quality satisfied the criteria for high-quality MRI studies. The finalized imaging parameters were maintained for the scanner during the entire MRI data acquisition period.

**Training workshops.** Pre-acquisition workshops were conducted to train researchers from different sites on the pipeline and procedural precautions of MRI data acquisition, scanner QC, and image quality assessments.

### QC during scanning

**Scanner QC.** To ensure scanner performance, researches performed regular phantom-based quality assessments for each scanner. The American College of Radiology (ACR) phantom was used to assess geometric distortion, slice positioning and thickness, high contrast spatial resolution, intensity uniformity, low contrast object detectability, and ghosting artefact, based on of T1- and T2-weighted ACR images. For detailed methods and criteria, please refer to the phantom guidance (https://www.acraccreditation.org/-/media/ACRAccreditation/Documents/MRI/LargePhantomGuidance.pdf?la=en). The custom phantom was used to evaluate temporal stability of fMRI data acquisition, based on 200 volumes obtained from the middle part of the phantom. For detailed methods and criteria, please refer to the documentation of the Function Biomedical Informatics Research Network (FBIRN) Stability phantom quality assurance procedures (https://www.nitrc.org/frs/download.php/275/fBIRN_phantom_qaProcedures.pdf). MRI data collection will be paused until any imaging quality issues are resolved.

**Checking imaging parameters.** Before MRI examination, researchers should confirm imaging parameters, which especially important for MRI scanners that were also used for clinical purposes.

**Confirming participant ID.** Researchers should confirm participant identity.

**Safety assessments.** Researchers should check safety items before participants entered the MRI room.

**Preventing head motion.** Participants were instructed to remain moveless, with foam padding minimizing movement. Rest breaks between sequences were allowed.

**Reducing scanner noise.** Participants wore MRI-compatible earplugs/headphones to reduce scanner noise.

**Standardizing participant instructions.** Standard instructions were provided to each participant. For example, we instructed participants to close their eyes, stay still, and avoid focused thoughts or sleep during the resting-state fMRI examination. After the examination, we asked the participants to confirm their compliance. The resting-state fMRI data should be re-acquired if the instructions were not followed.

**Screening brain abnormality.** T1-weighted and T2-weighted images were used for identifying lesions and structural abnormalities. Affected participants were excluded and informed.

**Inspecting image quality.** Images from each sequence were checked for artefacts, and the defective sequences should be re-acquired.

**Onsite parameter verification.** DICOM headers were reviewed to confirm parameters (e.g., FOV, slice thickness) matched pre-defined settings. Non-compliant images should be re-acquired.

**Head motion assessment.** The head motion of the resting-state fMRI data should be checked by the realign function of SPM 12. If the maximum displacement in any of the three orthogonal directions is more than 3 mm or a maximum rotation is greater than 3 degrees, the resting-state fMRI data should be re-acquired.

### QC immediately after scanning

**Confirming participant ID.** Researchers copied DICOM neuroimaging data from the scanner to a mobile disk, with each data type stored in a separate sub-folder. For each participant, researchers should verify the participant’s ID linked to neuroimaging data to prevent misassignment.

**Checking image counts.** To ensure complete data transfer (given rare slice loss during copying), researchers should check the number of slices for each sequence. MRI data should be stored on at least two independent disks for safety.

### QC before preprocessing

The TMUGH team was responsible for data QC before and during preprocessing.

**DICOM-to-NIFTI transformation.** To guarantee the accuracy of DICOM-to-NIFTI conversion, we developed a batch program for the conversion using dicm2nii version 2018.08.08 (<https://github.com/xiangruili/dicm2nii>), supporting multi-cluster parallel processing and standardized output.

**Brain abnormalities.** As a second check (following initial screening during scanning), experienced radiologists reviewed T2-weighted images and structural MRI images for lesions or abnormalities. Participants with confirmed abnormalities were excluded.

**Imaging quality.** We applied both automated and manual pipelines to assess imaging quality. We checked sequence parameters in the dcmHearders.mat file and excluded the MRI data with header parameters differing from the predefined ones. We also checked the coverage of the entire brain and excluded the participants with incomplete coverage. We then checked various artefacts in brain-containing slices, including aliasing artefact, ghosting artefact, metal artefact, and rare artefacts (zipper noise, spikes, radiofrequency overflow). The affected imaging data were excluded from the following analyses. For diffusion MRI, the "eddy" toolbox in FSL 5.0.10 was used to evaluate and correct motion-induced displacement, signal dropout, and eddy-current distortion. For resting-state fMRI, SPM 12 was used to evaluate head motion, participants with excessive head motion (maximum displacement > 3 mm in any direction or maximum rotation > 3 degrees) were excluded. We also calculated frame-wise displacement (FD) to index volume-to-volume changes in head position using the Jenkinson method[^31^](#_ENREF_1_31). When one volume’s FD exceeded 0.5 mm, this volume, its preceding volume, and the subsequent two volumes were defined as affected volumes. Participants were excluded if their mean FD exceeded 0.5 mm or if affected volumes accounted for more than one-third of the total volumes.

### Quality control during preprocessing

We developed a CHIMGEN pipeline to preprocess all multi-model neuroimaging data. The pipeline integrated various preprocessing procedures and tools with multi-cluster parallel computation. The total computing time was greatly reduced by using the Tianhe super-computer. The preprocessing pipeline for each type of MRI data were described before. We also checked the preprocessed MRI data to identify errors or imperfections emerged during imaging data preprocessing, including poor segmentation and spatial normalization during structural image preprocessing for GMV calculation; incorrect removal of non-brain tissue, normalization intensity error, misplacement of the pial surface, and topological defects during structural image preprocessing for CT and SA calculation; improper removal of non-brain tissue, imperfect spatial normalization during diffusion MRI data preprocessing; and flawed spatial normalization during fMRI data preprocessing. When an error was identified, we tried to find the reason and then repeated the pipeline after fixing the error. If the error still existed, we had to exclude the participant in the IDP extraction from the problematic imaging modality. As mentioned in the fMRI data preprocessing, we also excluded the participants with over excessive head motion. After quality control, we extracted 127 GMV-IDPs, 64 CT-IDPs, and 64 SA-IDPs from 7,182 participants, 18 Amp-IDPs, 153 FC-IDPs, and 124 ReHo-IDPs from 6,283 participants, and 48 FA-IDPs from 7,153 participants (**Table S1**).

## References:

1 Xu, Q. *et al.* CHIMGEN: a Chinese imaging genetics cohort to enhance cross-ethnic and cross-geographic brain research. *Molecular psychiatry* **25**, 517-529, doi:10.1038/s41380-019-0627-6 (2020).

2 Gorelick, N., Hancher, M., Dixon, M., Ilyushchenko, S. & Moore, R. Google Earth Engine: Planetary-scale geospatial analysis for everyone. *Remote Sensing of Environment* **202** (2017).

3 Xu, Y. *et al.* Annual 30-m land use/land cover maps of China for 1980–2015 from the integration of AVHRR, MODIS and Landsat data using the BFAST algorithm. *Science China Earth Sciences* **63**, 1390-1407, doi:10.1007/s11430-019-9606-4 (2020).

4 Yu, L. *et al.* FROM-GLC Plus: toward near real-time and multi-resolution land cover mapping. *GIScience & Remote Sensing* **59**, 1026-1047, doi:10.1080/15481603.2022.2096184 (2022).

5 Lloyd, C. T. *et al.* Global spatio-temporally harmonised datasets for producing high-resolution gridded population distribution datasets. *Big earth data* **3**, 108-139, doi:10.1080/20964471.2019.1625151 (2019).

6 Zha, Y., Gao, J. & Ni, S. Use of normalized difference built-up index in automatically mapping urban areas from TM imagery. *International journal of remote sensing* **24**, 583-594 (2003).

7 Huang, S., Tang, L., Hupy, J. P., Wang, Y. & Shao, G. A commentary review on the use of normalized difference vegetation index (NDVI) in the era of popular remote sensing. *Journal of Forestry Research* **32**, 1-6, doi:10.1007/s11676-020-01155-1 (2020).

8 Elvidge, C. D., Baugh, K. E., Kihn, E. A., Kroehl, H. W. & Davis, E. R. Mapping city lights with nighttime data from the DMSP Operational Linescan System. *Photogrammetric Engineering and Remote Sensing* **63**, 727-734 (1997).

9 Van Donkelaar, A. *et al.* Global estimates of fine particulate matter using a combined geophysical-statistical method with information from satellites, models, and monitors. *Environmental science & technology* **50**, 3762-3772 (2016).

10 Geddes, J. A., Martin, R. V., Boys, B. L. & van Donkelaar, A. Long-term trends worldwide in ambient NO2 concentrations inferred from satellite observations. *Environmental health perspectives* **124**, 281-289 (2016).

11 Alley, W. M. The Palmer Drought Severity Index: Limitations and Assumptions. *Journal of Applied Meteorology and Climatology* **23**, 1100-1109, doi:https://doi.org/10.1175/1520-0450(1984)023<1100:TPDSIL>2.0.CO;2 (1984).

12 Abatzoglou, J. T., Dobrowski, S. Z., Parks, S. A. & Hegewisch, K. C. TerraClimate, a high-resolution global dataset of monthly climate and climatic water balance from 1958-2015. *Scientific data* **5**, 170191, doi:10.1038/sdata.2017.191 (2018).

13 Becker, E. *et al.* The NCEP Climate Forecast System Version 2. *Journal of Climate* **27**, 2185-2208, doi:10.1175/jcli-d-12-00823.1 (2014).

14 Jiang, L. & Yu, L. Analyzing land use intensity changes within and outside protected areas using ESA CCI-LC datasets. *Global Ecology and Conservation* **20**, e00789, doi:10.1016/j.gecco.2019.e00789 (2019).

15 Zhang, Y., Odeh, I. O. A. & Han, C. Bi-temporal characterization of land surface temperature in relation to impervious surface area, NDVI and NDBI, using a sub-pixel image analysis. *International Journal of Applied Earth Observation and Geoinformation* **11**, 256-264, doi:10.1016/j.jag.2009.03.001 (2009).

16 Henderson, V., Squires, T., Storeygard, A. & Weil, D. THE GLOBAL DISTRIBUTION OF ECONOMIC ACTIVITY: NATURE, HISTORY, AND THE ROLE OF TRADE. *The quarterly journal of economics* **133**, 357-406, doi:10.1093/qje/qjx030 (2018).

17 Tatem, A. J. WorldPop, open data for spatial demography. *Scientific data* **4**, 170004, doi:10.1038/sdata.2017.4 (2017).

18 Saha, S. *et al.* The NCEP Climate Forecast System Reanalysis. *Bulletin of the American Meteorological Society* **91**, 1015-1058, doi:10.1175/2010bams3001.1 (2010).

19 Van Donkelaar, A. *et al.* (NASA Socioeconomic Data and Applications Center (SEDAC), Palisades, NY, 2018).

20 Geddes, J. A., Martin, R. V., Boys, B. L. & van Donkelaar, A. (NASA Socioeconomic Data and Applications Center (SEDAC), Palisades, New York, 2017).

21 Jarvis, A., Reuter, H., Nelson, A. & Guevara, E. Hole-filled seamless SRTM data v4. *International Centre for Tropical Agriculture (CIAT)* (2008).

22 Bernstein, D. P., Ahluvalia, T., Pogge, D. & Handelsman, L. Validity of the Childhood Trauma Questionnaire in an adolescent psychiatric population. *Journal of the American Academy of Child and Adolescent Psychiatry* **36**, 340-348, doi:10.1097/00004583-199703000-00012 (1997).

23 Xu, J. *et al.* Global urbanicity is associated with brain and behaviour in young people. *Nature human behaviour* **6**, 279-293, doi:10.1038/s41562-021-01204-7 (2022).

24 Zhao, X. F., Zhang, Y. L., Li, L. F., Zhou, Y. F. & Yang, S. C. Reliability and validity of the Chinese version of childhood trauma questionnaire. *Chinese Journal of Clinical Rehabilitation* **9**, 105-107 (2005).

25 Rajapakse, J. C., Giedd, J. N. & Rapoport, J. L. Statistical approach to segmentation of single-channel cerebral MR images. *IEEE transactions on medical imaging* **16**, 176-186, doi:10.1109/42.563663 (1997).

26 Ashburner, J. A fast diffeomorphic image registration algorithm. *Neuroimage* **38**, 95-113, doi:10.1016/j.neuroimage.2007.07.007 (2007).

27 Tzourio-Mazoyer, N. *et al.* Automated anatomical labeling of activations in SPM using a macroscopic anatomical parcellation of the MNI MRI single-subject brain. *Neuroimage* **15**, 273-289, doi:10.1006/nimg.2001.0978 (2002).

28 Diedrichsen, J., Balsters, J. H., Flavell, J., Cussans, E. & Ramnani, N. A probabilistic MR atlas of the human cerebellum. *Neuroimage* **46**, 39-46, doi:10.1016/j.neuroimage.2009.01.045 (2009).

29 Pruim, R. H. R. *et al.* ICA-AROMA: A robust ICA-based strategy for removing motion artifacts from fMRI data. *Neuroimage* **112**, 267-277, doi:10.1016/j.neuroimage.2015.02.064 (2015).

30 Jiang, L. & Zuo, X. N. Regional Homogeneity: A Multimodal, Multiscale Neuroimaging Marker of the Human Connectome. *The Neuroscientist : a review journal bringing neurobiology, neurology and psychiatry* **22**, 486-505, doi:10.1177/1073858415595004 (2016).

31 Jenkinson, M., Bannister, P., Brady, M. & Smith, S. Improved optimization for the robust and accurate linear registration and motion correction of brain images. *Neuroimage* **17**, 825-841, doi:10.1016/s1053-8119(02)91132-8 (2002).

# Supplementary figures

**
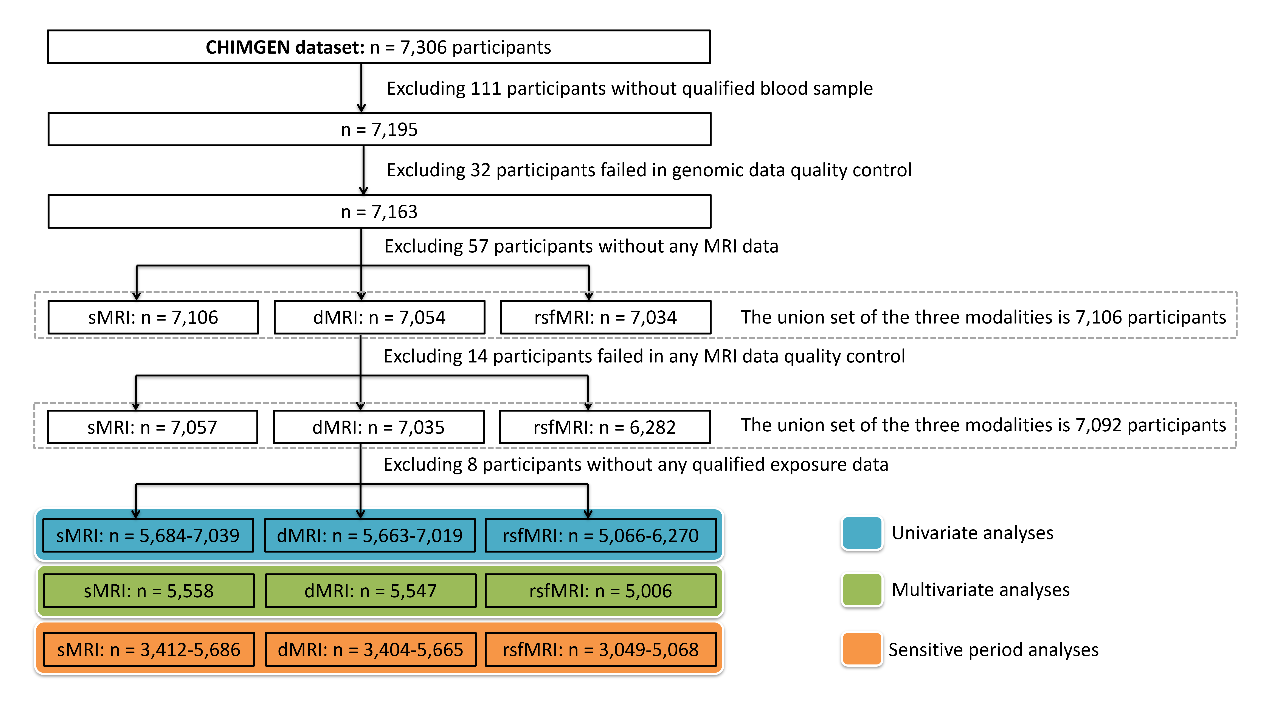
**

**Fig. S1*.* Participant selection for each statistical analysis.** We apply the principle of maximizing sample size to include as many participants as possible in each statistical analysis, and finally include 5,066-7,039 participants with complete genomic, environmental, and brain imaging data for univariate analyses, 5,006-5,558 for multivariate analyses, and 3,049-5,686 for sensitive period analyses. Abbreviations: CHIMGEN, Chinese Imaging Genetics; dMRI, diffusion magnetic resonance imaging; rsfMRI: resting-state functional MRI; sMRI: structural MRI.


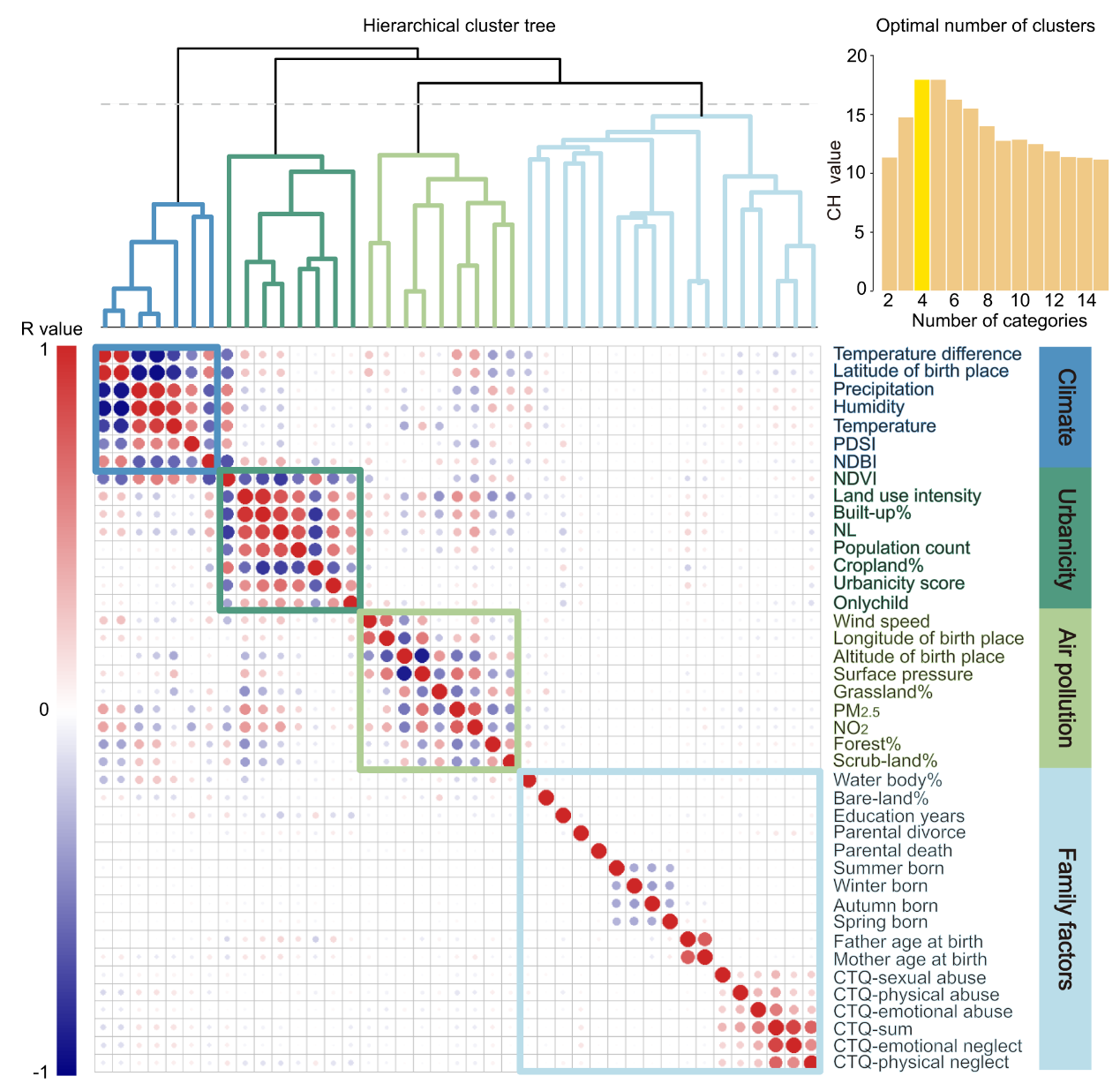


**Fig. S2. Hierarchical clustering analysis of 41 environmental exposures.** The heat map illustrates the Spearman correlation coefficient matrix for the 41 environmental exposures. Different colored boxes indicate distinct categories. The upper left panel presents the clustering tree, while the upper right panel displays CH values at different numbers of categories. The highest CH value (17.98) is observed when all exposures are grouped into four distinct categories.


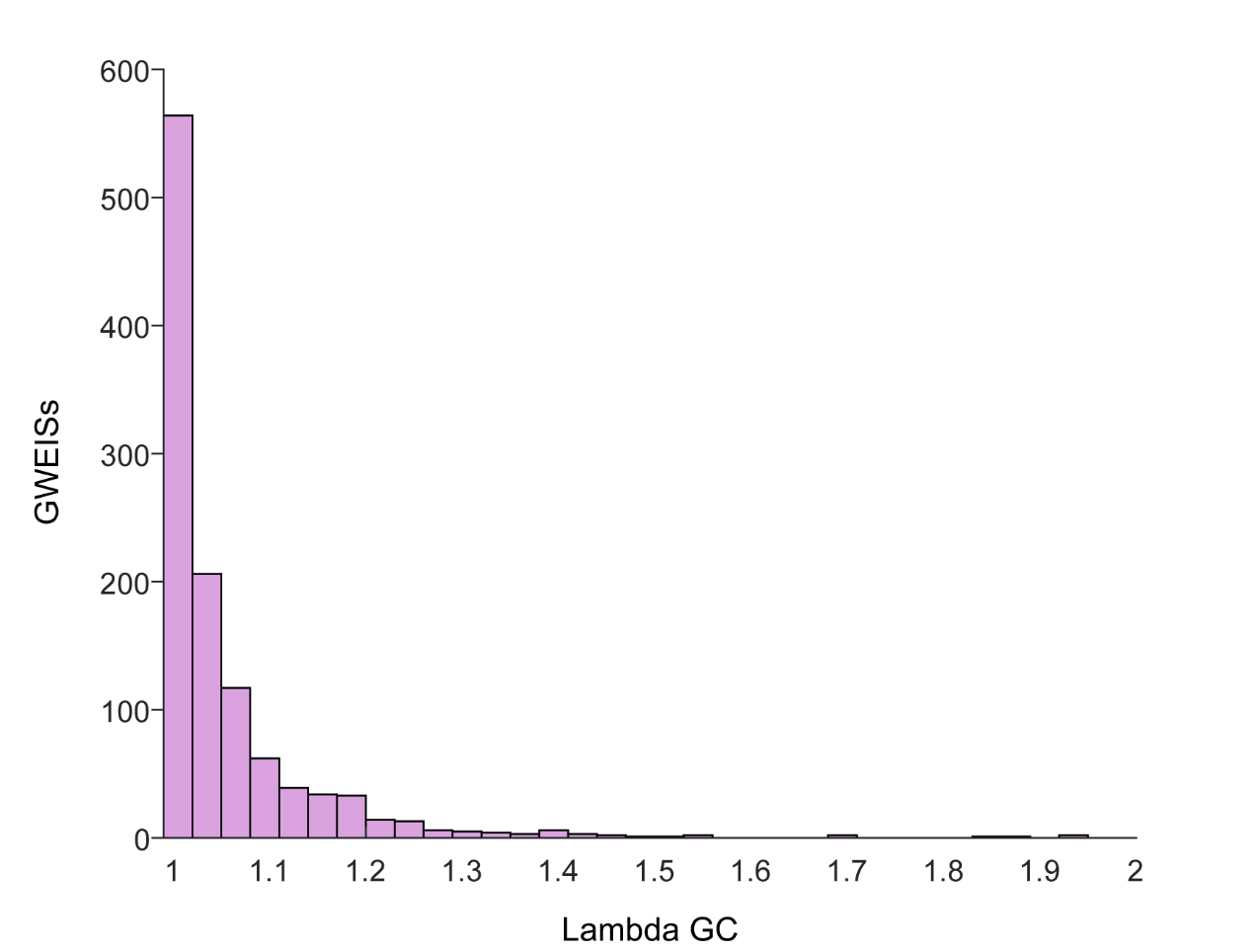


**Fig. S3. Quality control of univariate GWEISs of brain IDPs.** The histogram shows the lambda GC distribution of the 1,121 GWEISs with genome-wide significant G × E interactions (*P* < 5 × 10^-8^) on brain IDPs. Some GWEISs have lambda GC values deviated from one, indicating genomic inflation. Abbreviations: GWEISs, genome-wide by environment interaction studies; IDP, imaging-derived phenotype; lambda GC, genomic control inflation factor.


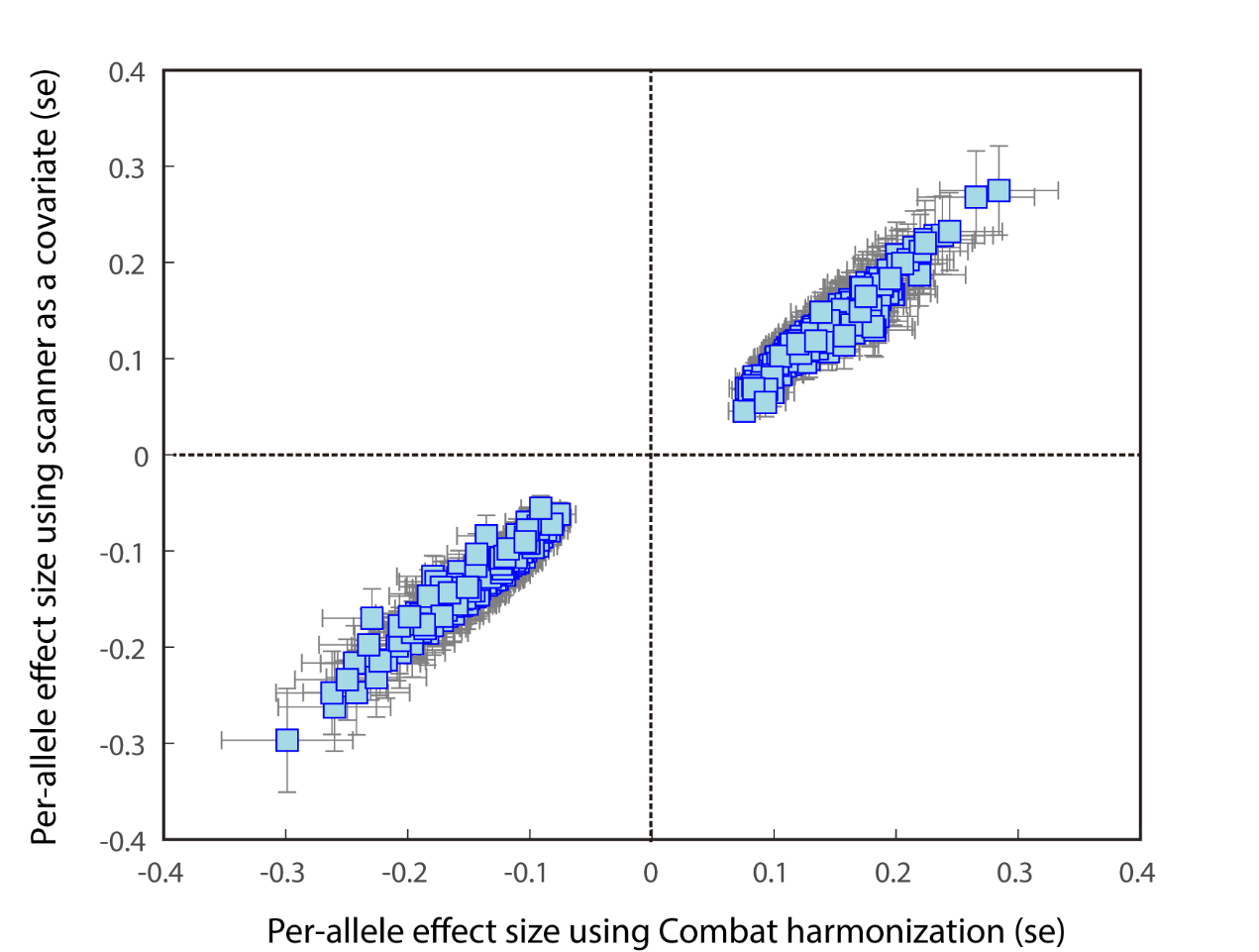


**Fig. S4. Consistency between adjustment methods for reducing the scanner effect.** Combat harmonization is applied to brain IDPs to reduce the scanner effect, allowing us to identify 486 genome-wide significant G × E interactions. We recalculate these interactions using IDPs without Combat harmonization, while including the scanner as a covariate. Scatter plots show a strong correlation (*rho* = 0.99, *P* < 1.00 × 10^-322^) of the effect sizes (beta values) derived from the two adjustment methods. Data are presented as beta values ± SE.


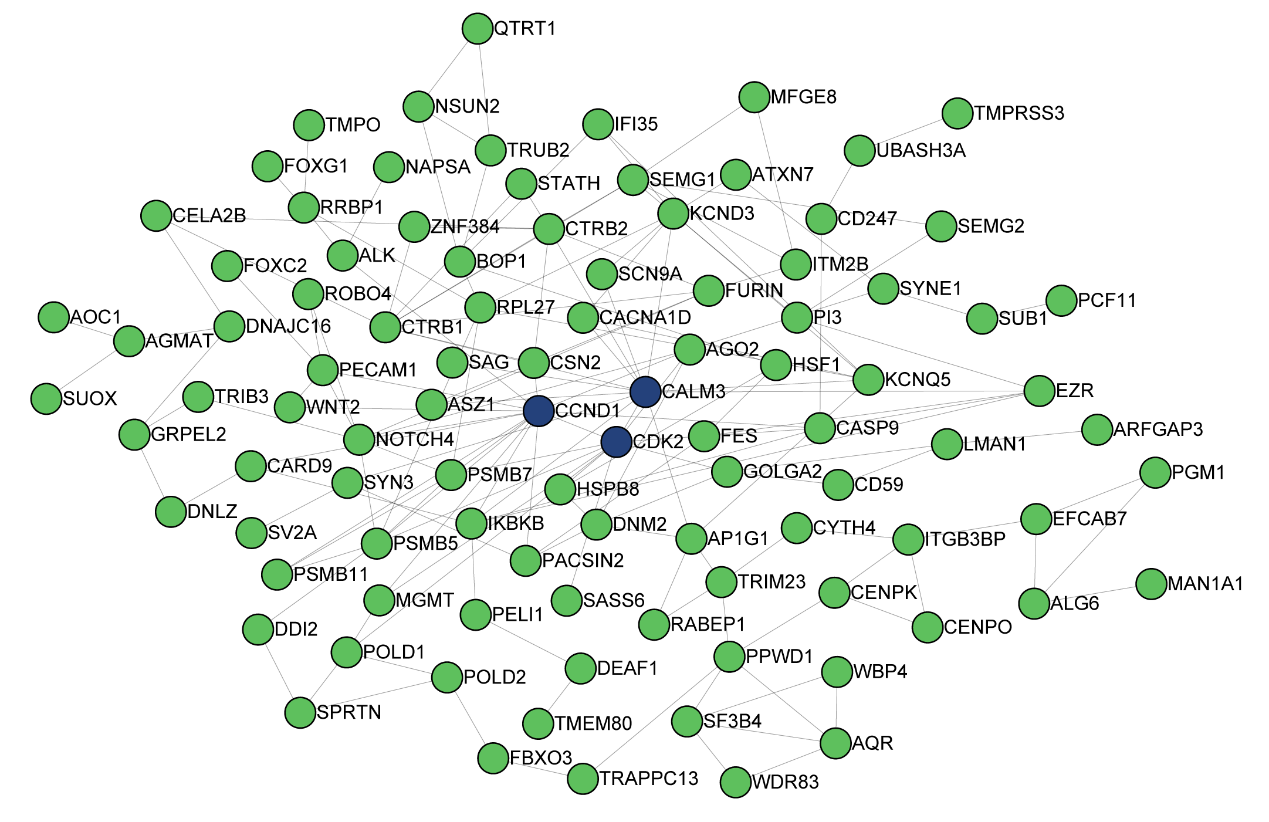


**Fig. S5. PPI analysis of genes interacting with urbanicity exposures on brain IDPs.** The network displays genes with the top 100 highest degrees of connectivity. The top three hub genes are highlighted with blue dots.

**
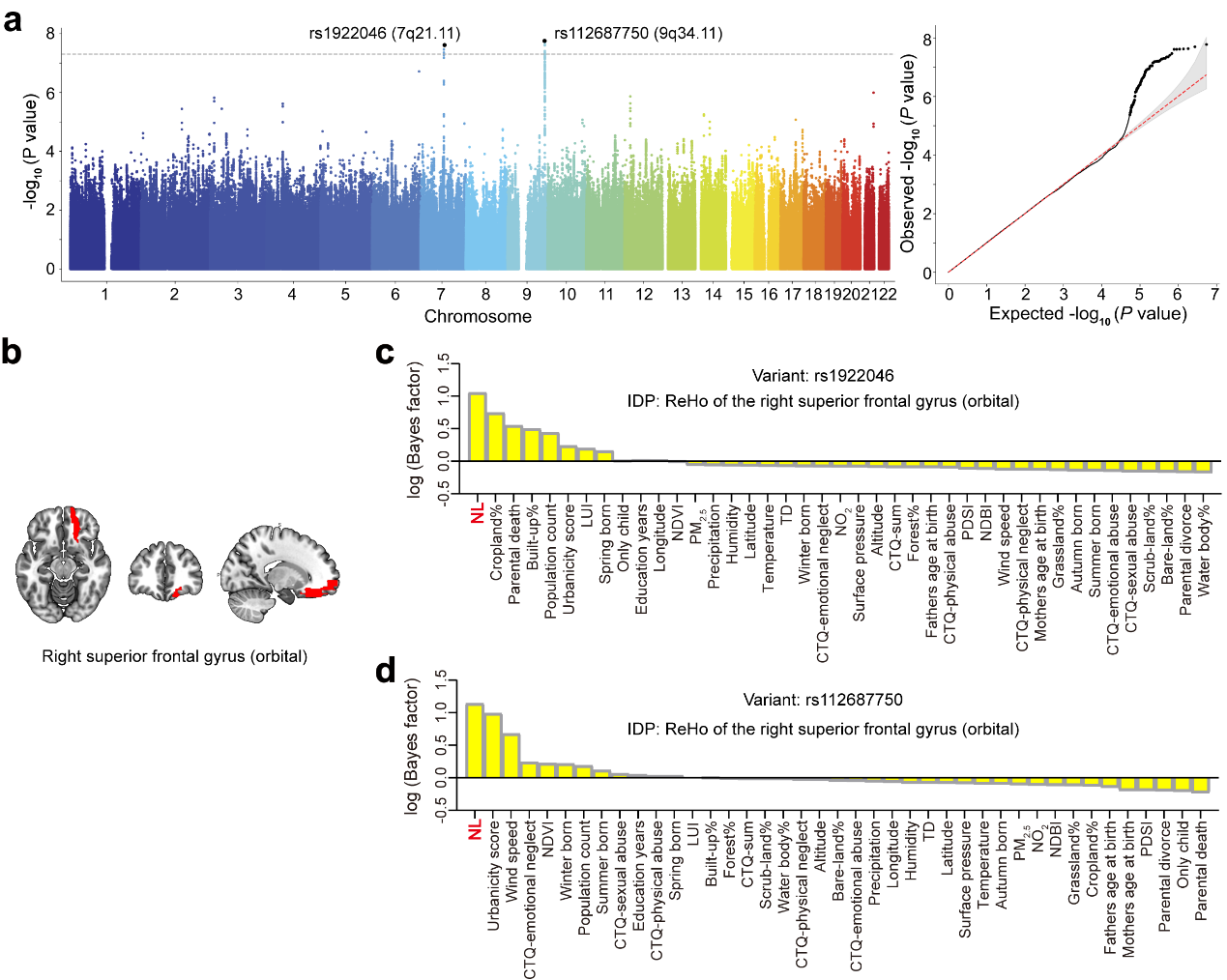
**

**Fig. S6*.* An example of the same exposure showing interactions with different genetic variants on the same brain IDP. a, b,** The Manhattan and quantile-quantile (QQ) plots (**a**) show two significant G × E interactions (*P* < 5 × 10^-8^, gray line) between rs1922046 and rs112687750 and NL on the ReHo of the orbital part of superior frontal gyrus (**b**). **c, d,** NL shows the largest Bayes factor among the 41 exposures in both multivariate models for the variant-IDP pairs of rs1922046 (**c**) and rs112687750 (**d**) with this IDP. Abbreviation: CTQ, childhood trauma questionnaire; IDP, imaging-derived phenotype; LUI, land use intensity; NDBI, normalized difference built-up index; NDVI, normalized difference vegetation index; NL, night-time light; NO_2_, nitrogen dioxide; PDSI, Palmer drought severity index; PM, particular matter; ReHo, regional homogeneity; TD, temperature difference.

**
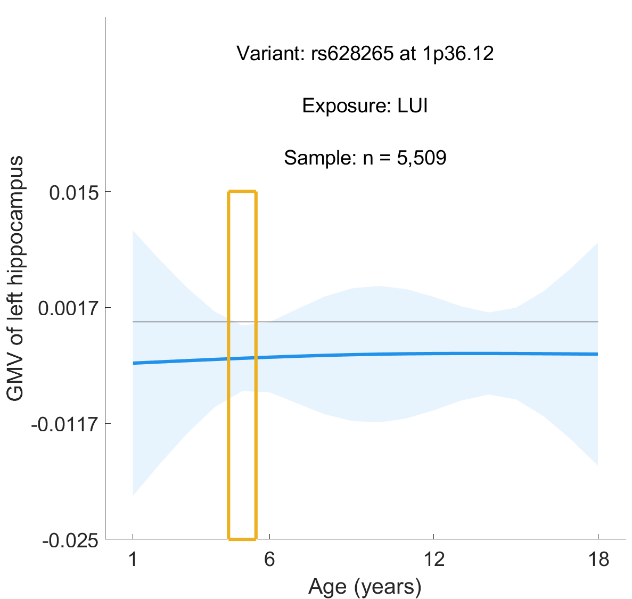

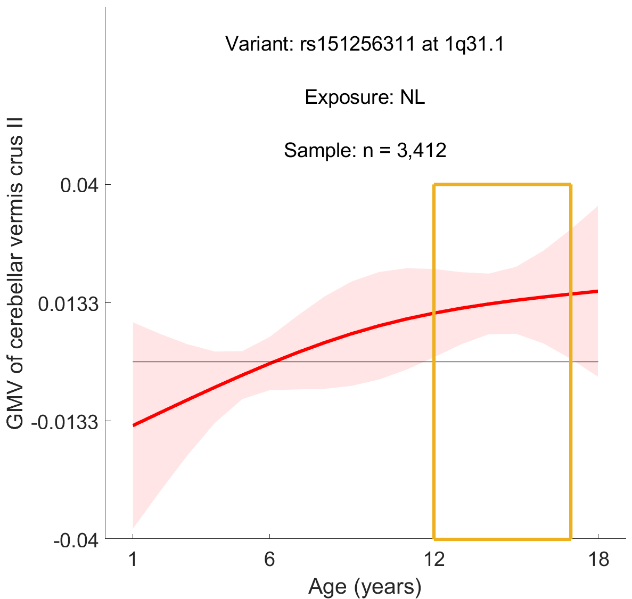

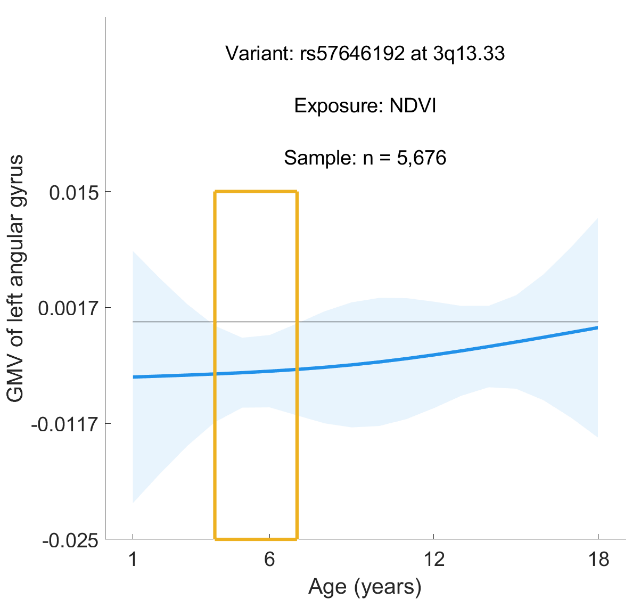

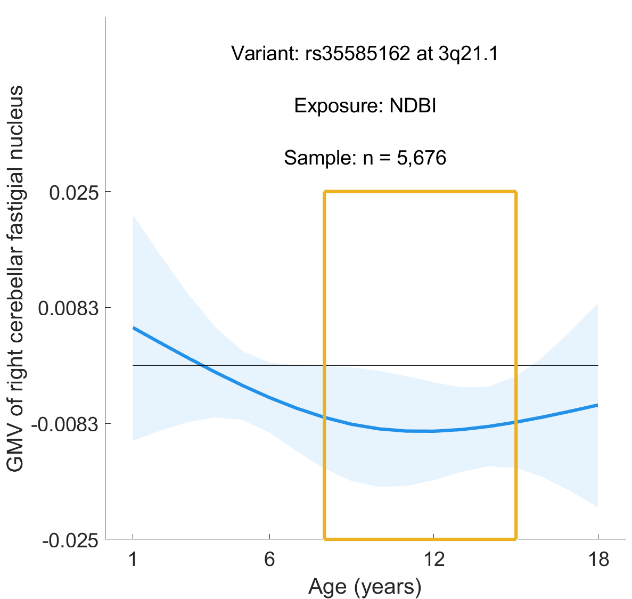

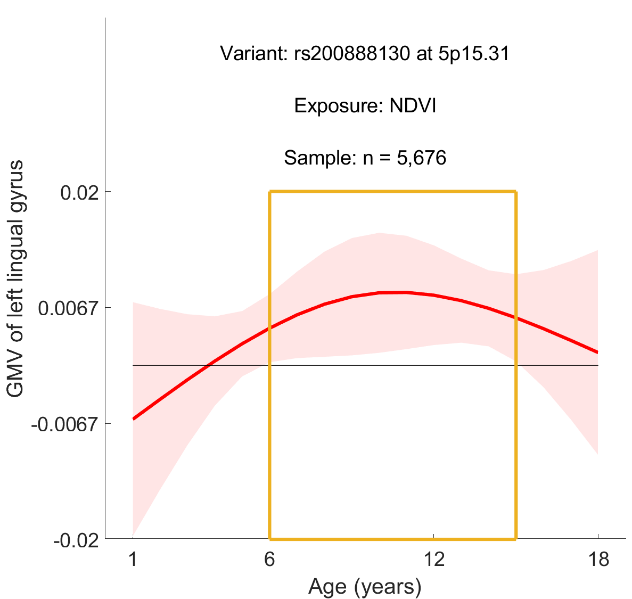

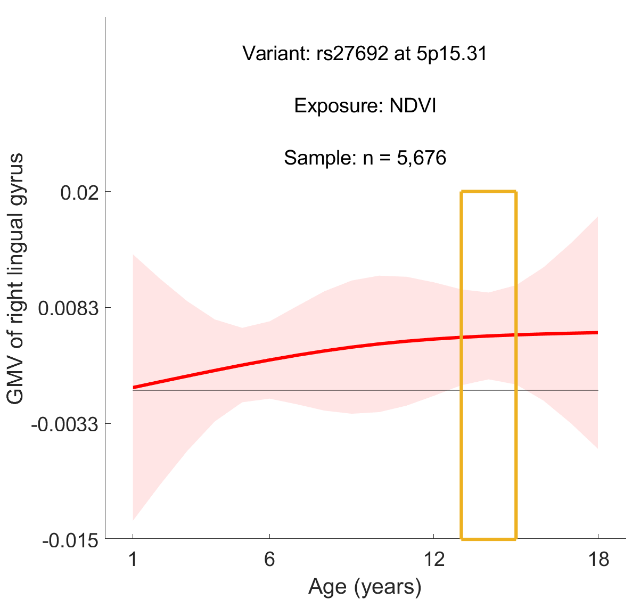

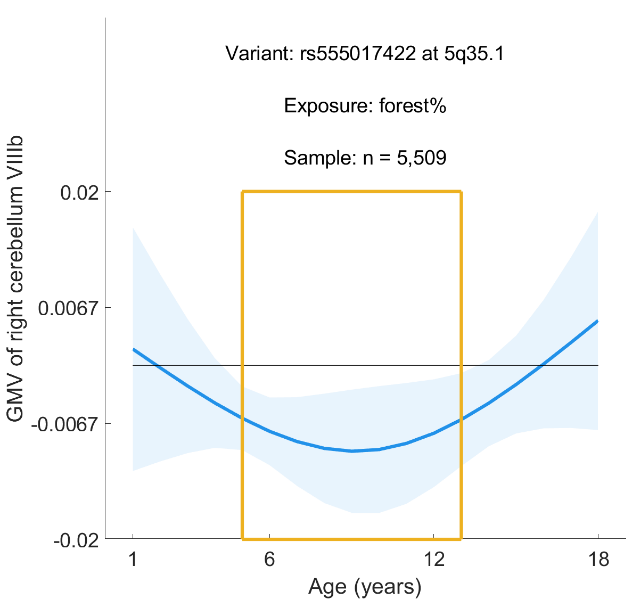

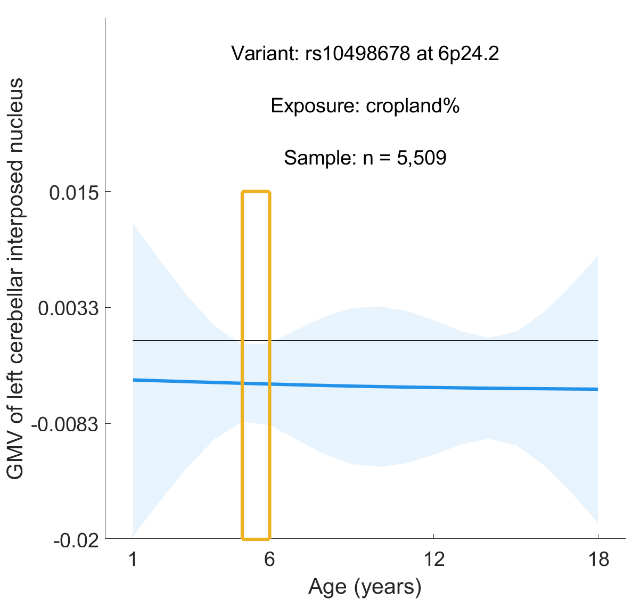

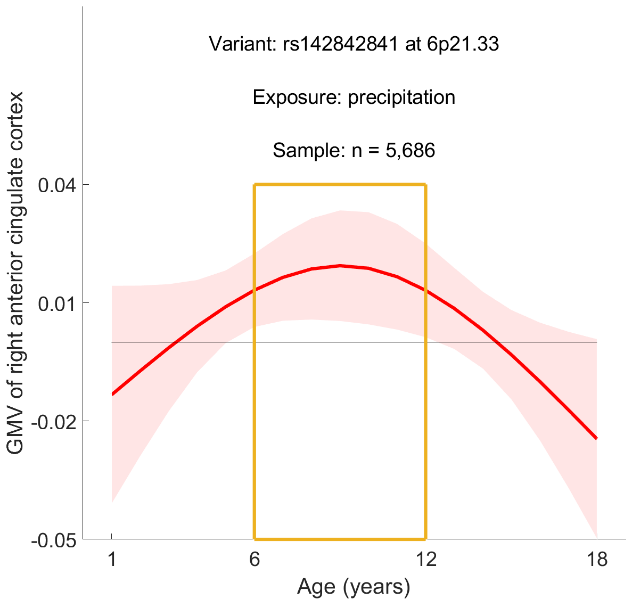

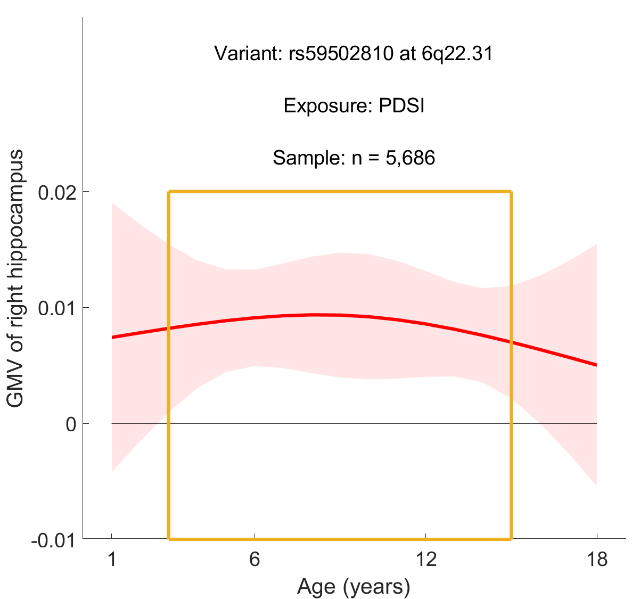

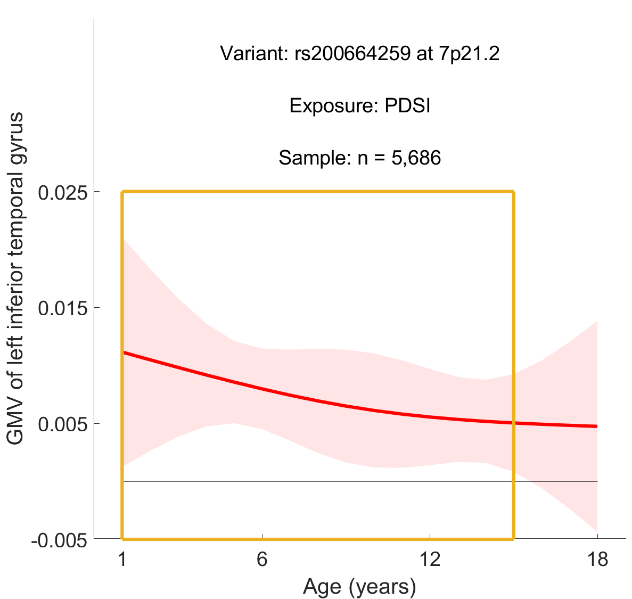

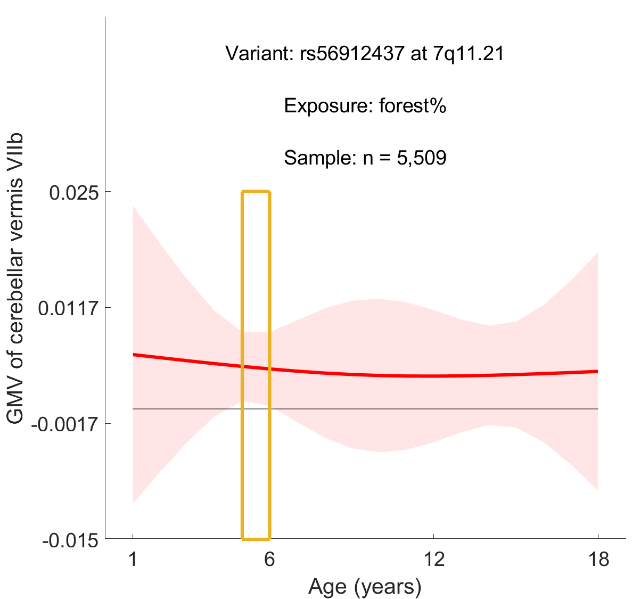

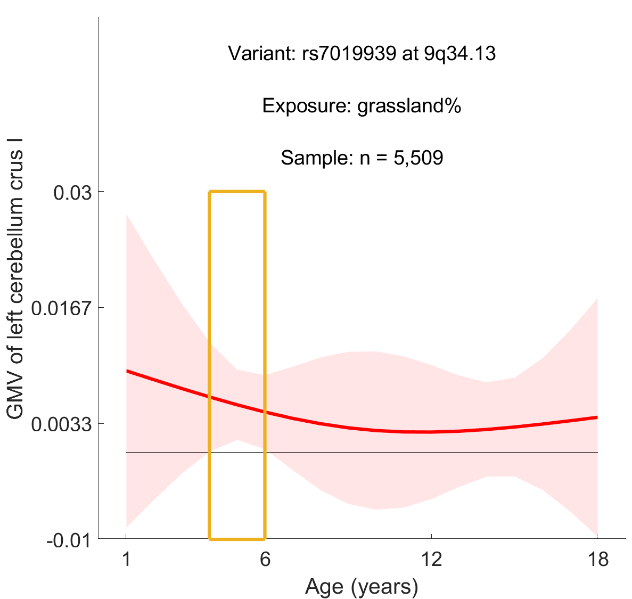

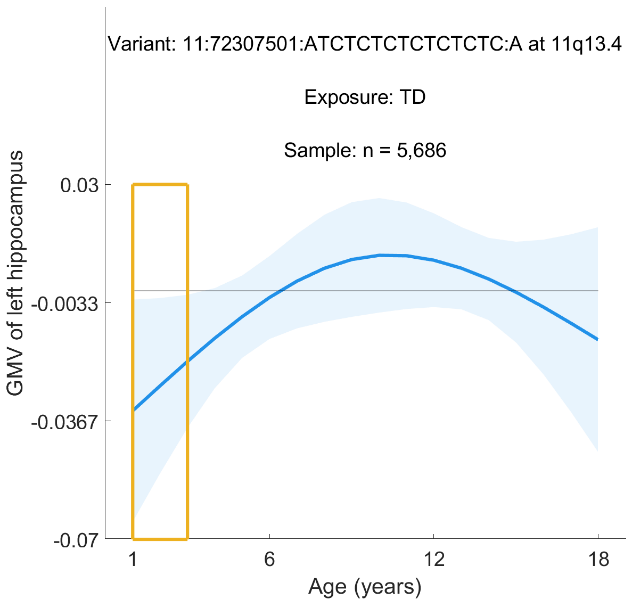

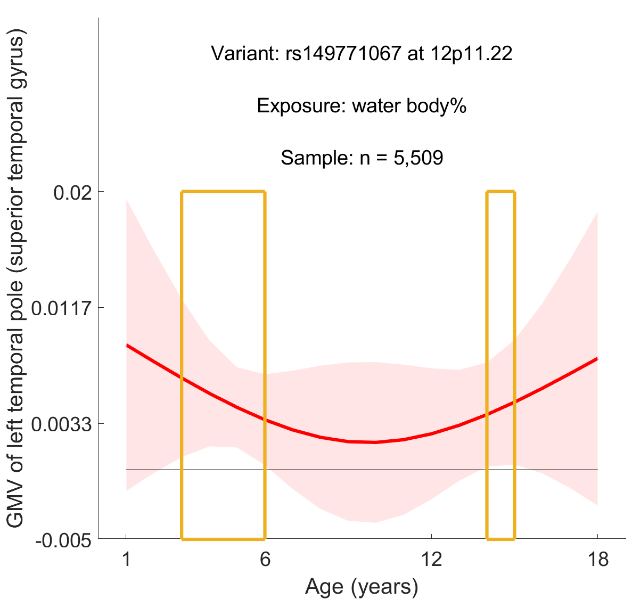

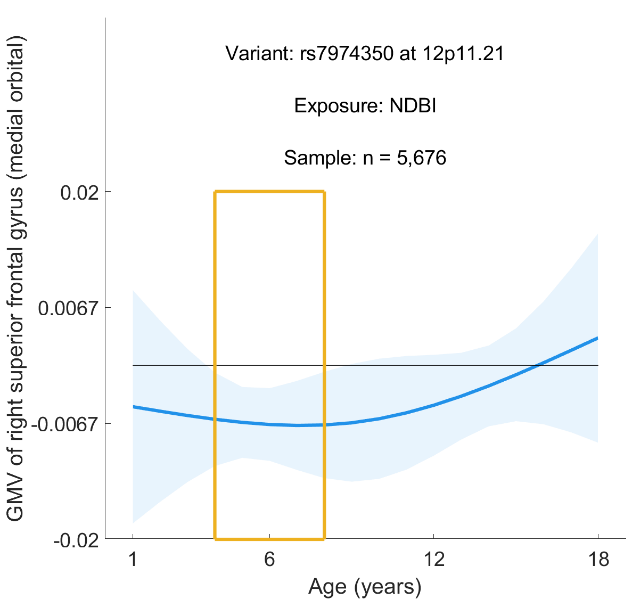

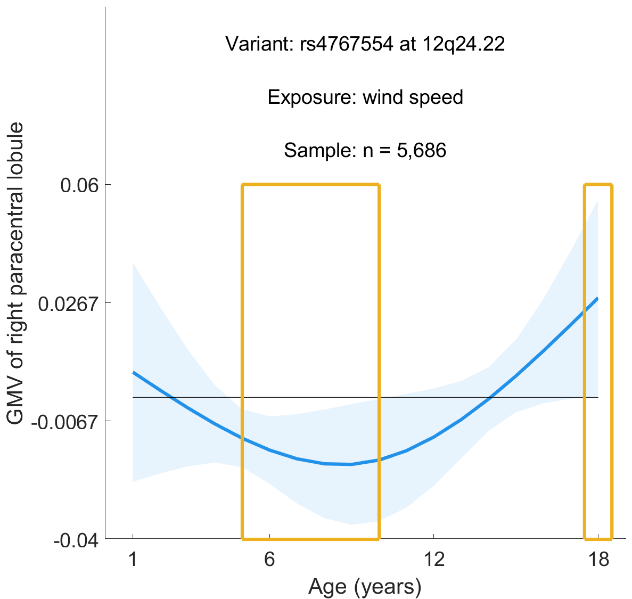

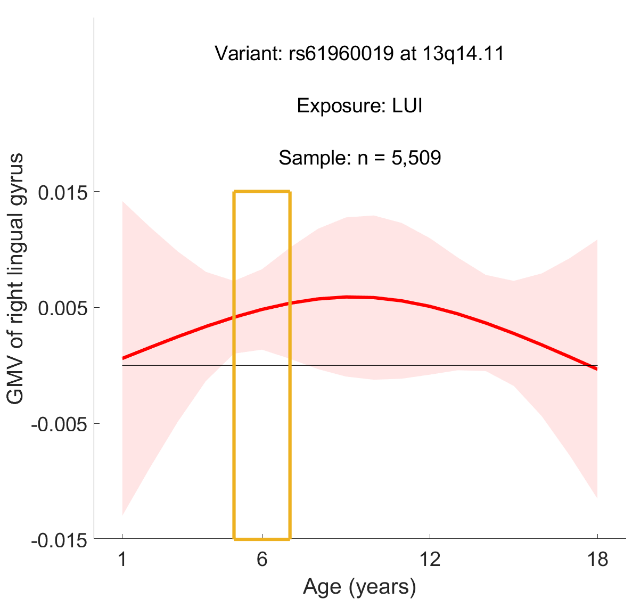

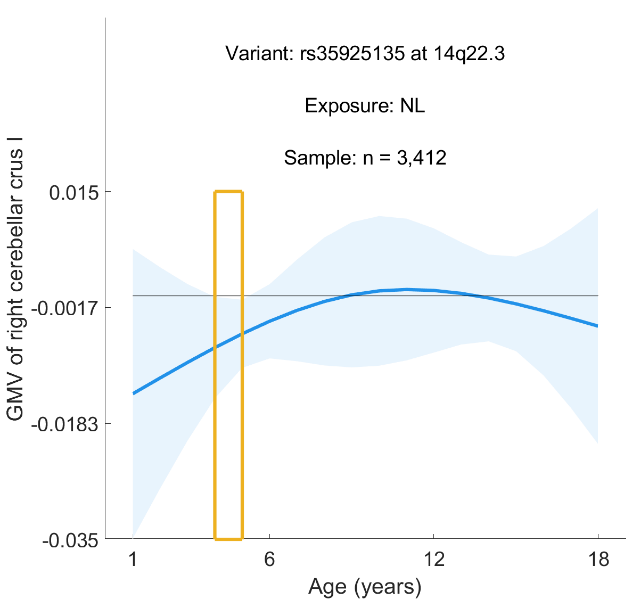

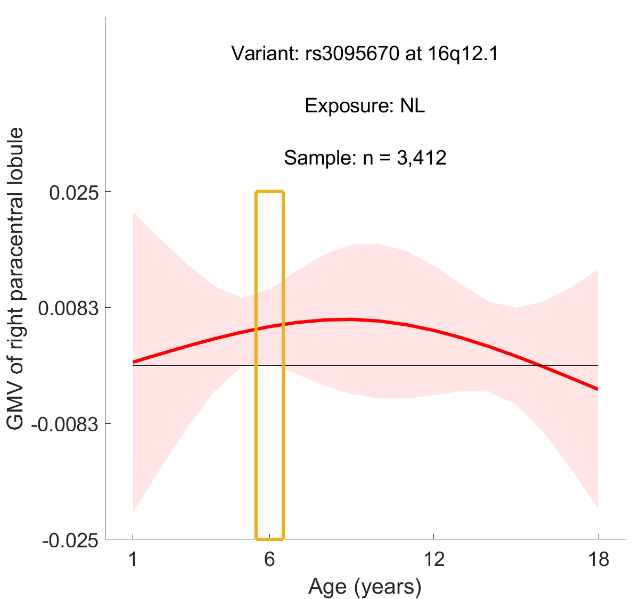

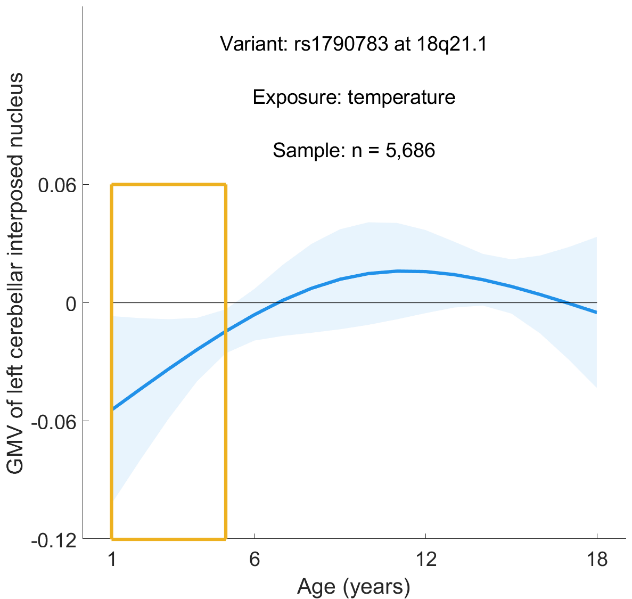

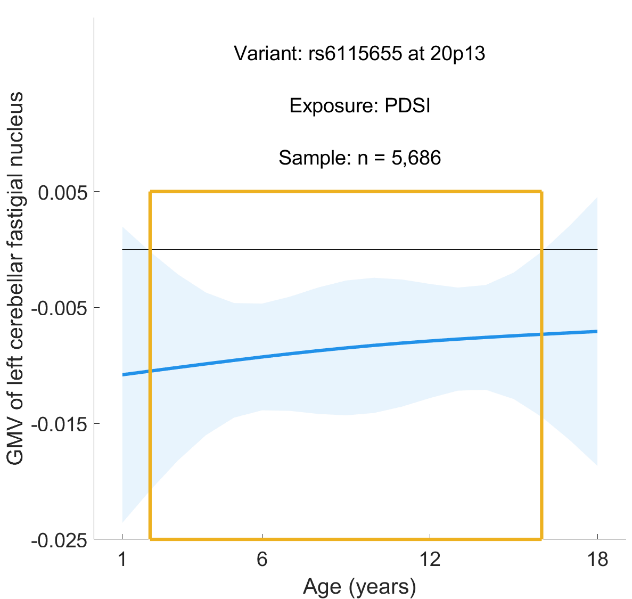

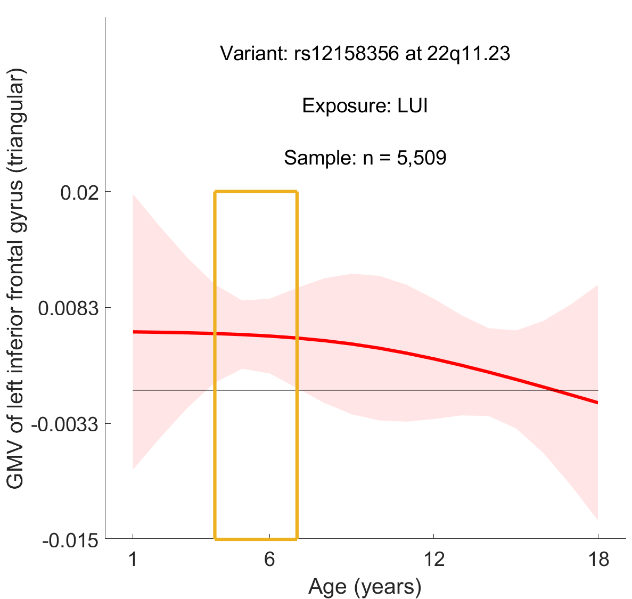
**

**Fig. S7. Sensitive periods for G × E interactions on GMV-IDPs.** Each plot shows the sensitive period of one G × E interaction on a given GMV-IDP. A sensitive period is defined as the period during which the estimated pointwise 95% CI does not include zero. Abbreviations: CI, confidence interval; GMV, gray matter volume; IDP, imaging-derived phenotype; LUI, land use intensity; NDBI, normalized difference built-up index; NDVI, normalized difference vegetation index; NL, night-time light; PDSI, Palmer drought severity index; TD, temperature difference.

**
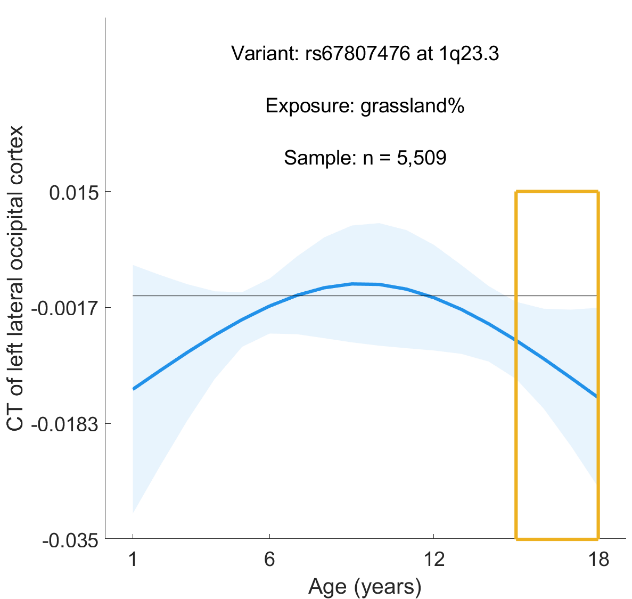

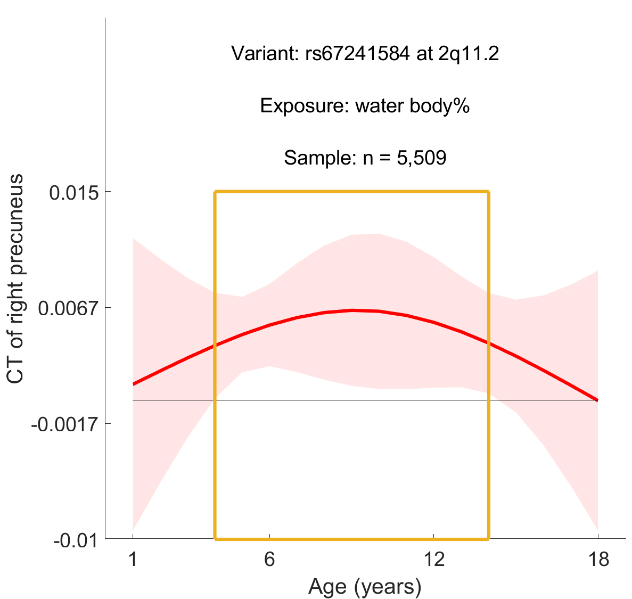

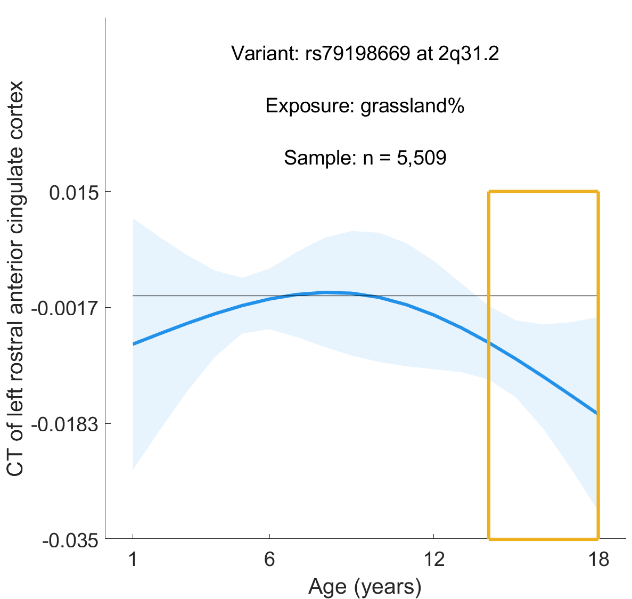

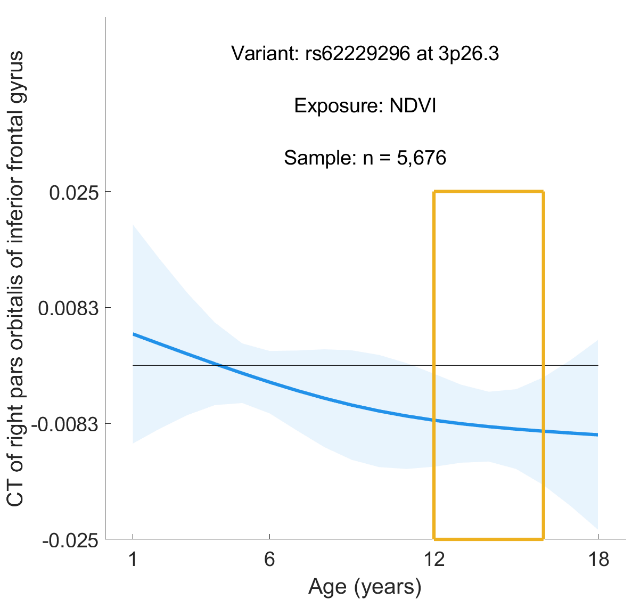

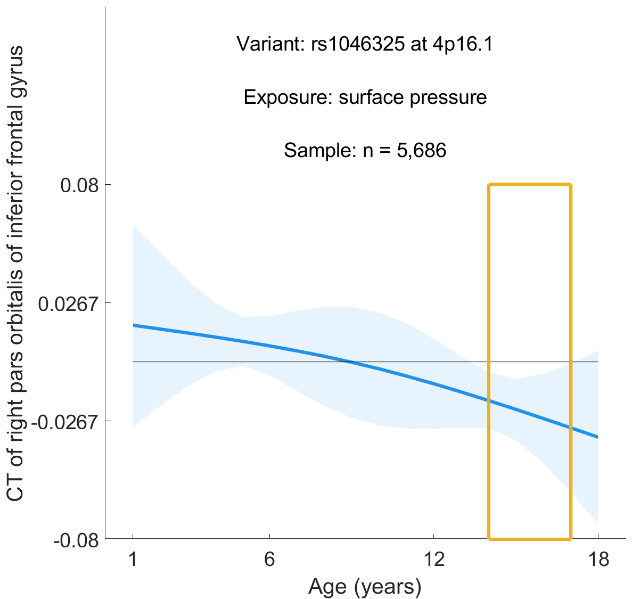

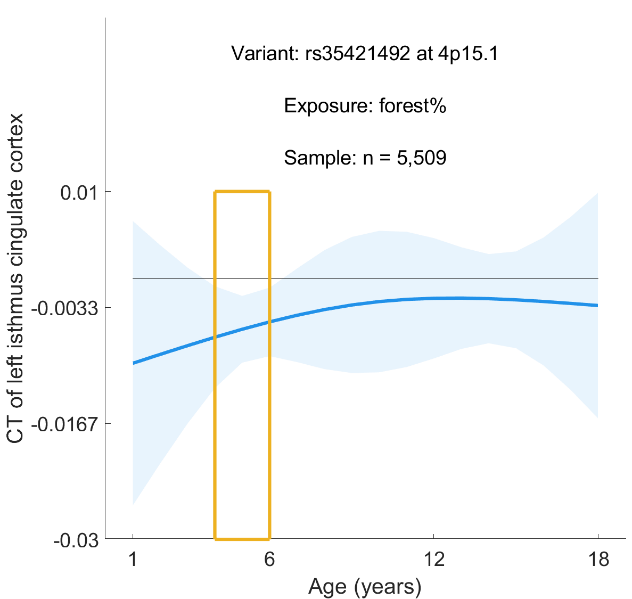

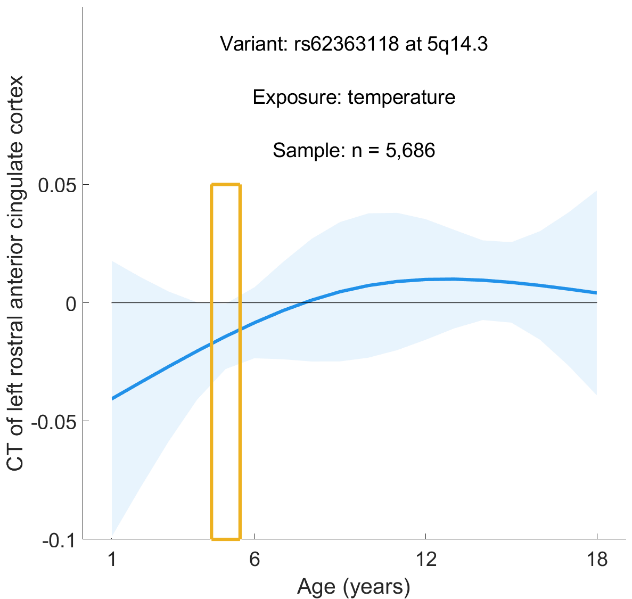

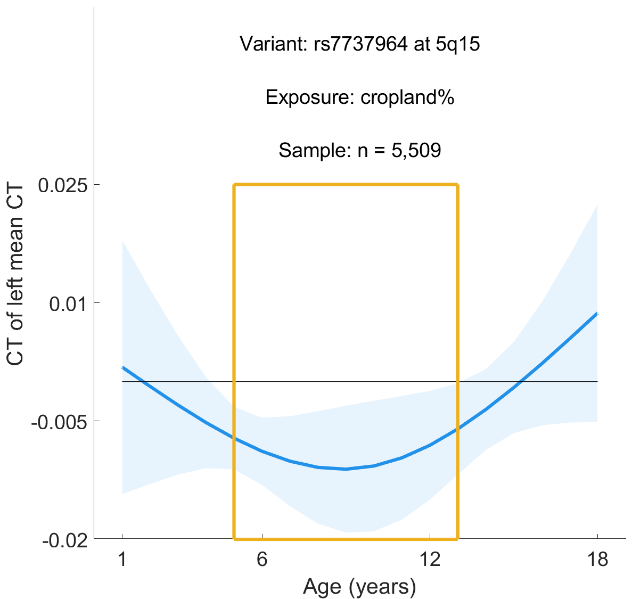

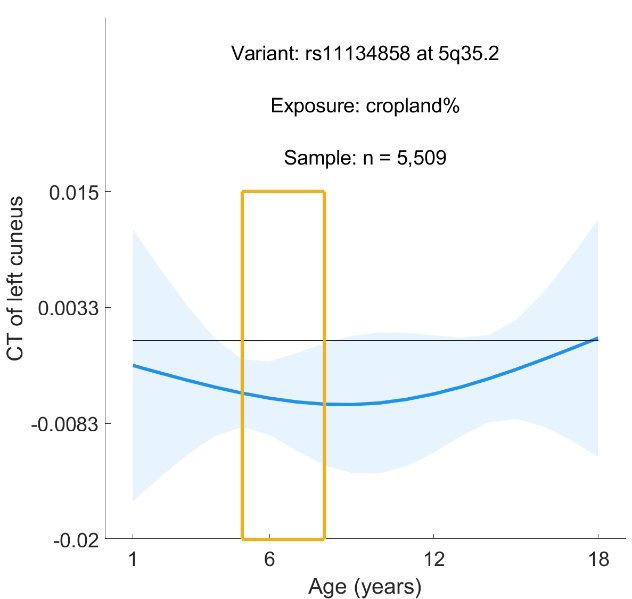

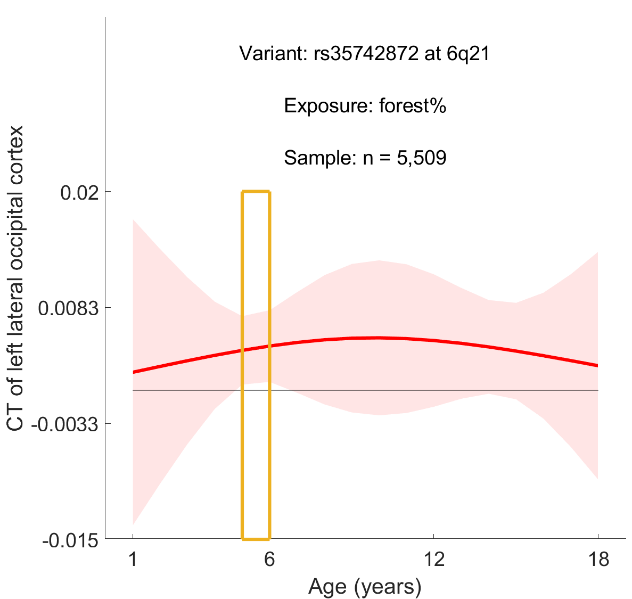

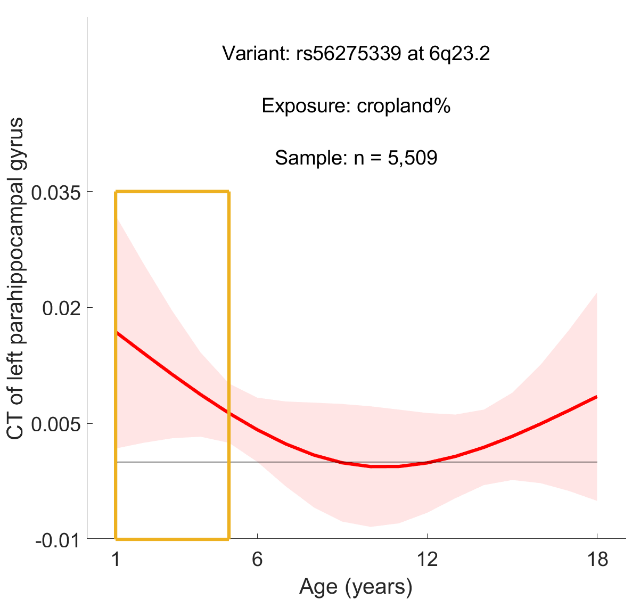

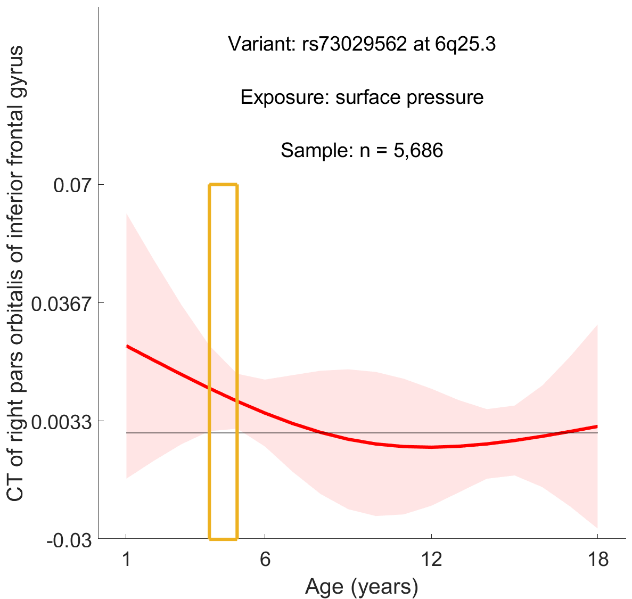

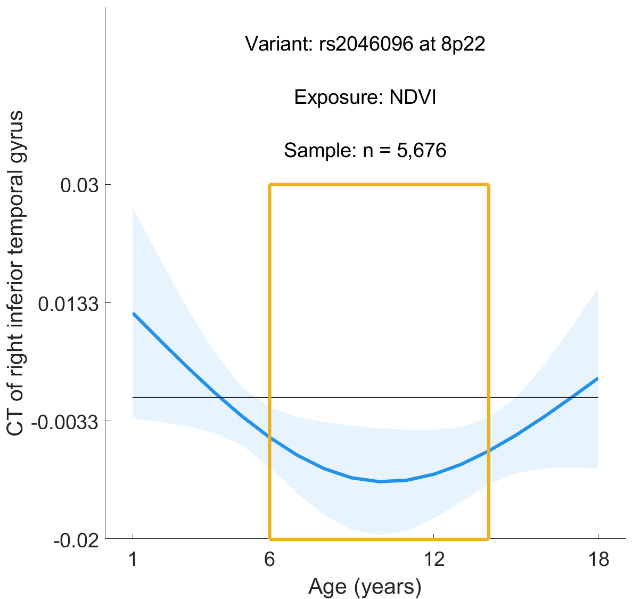

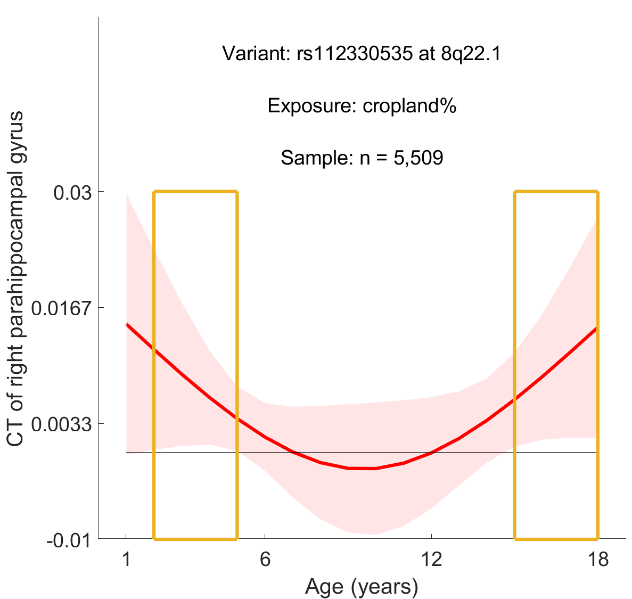

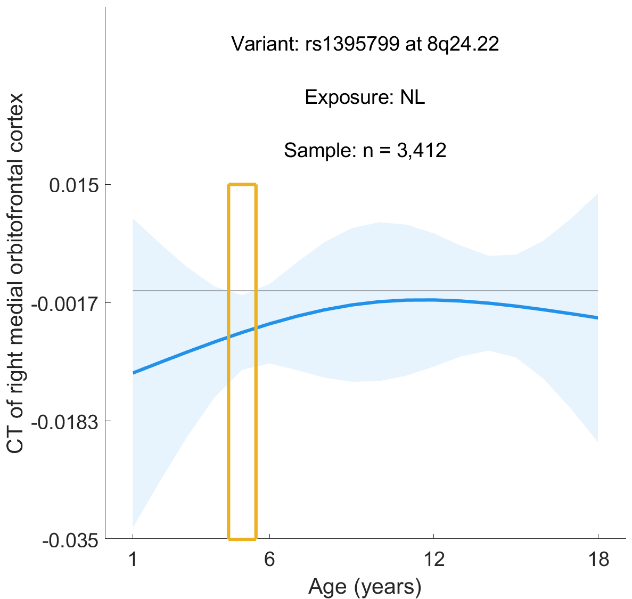

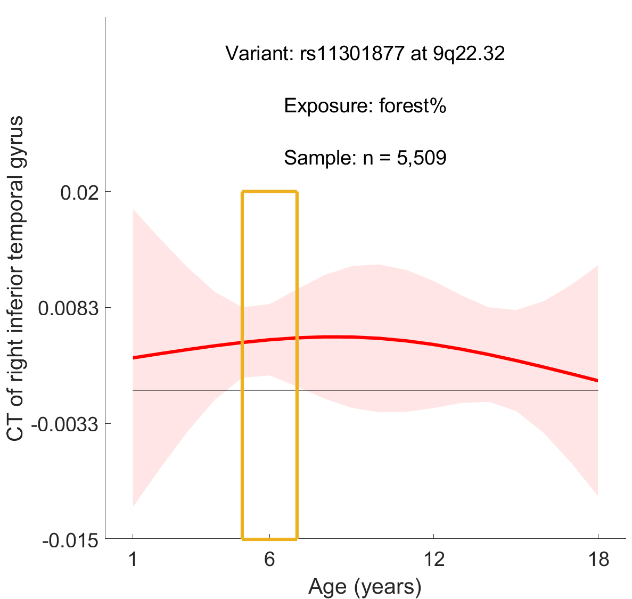

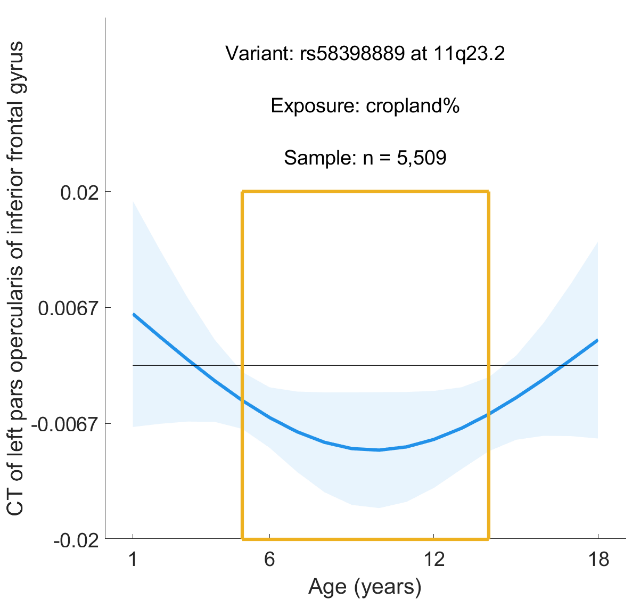

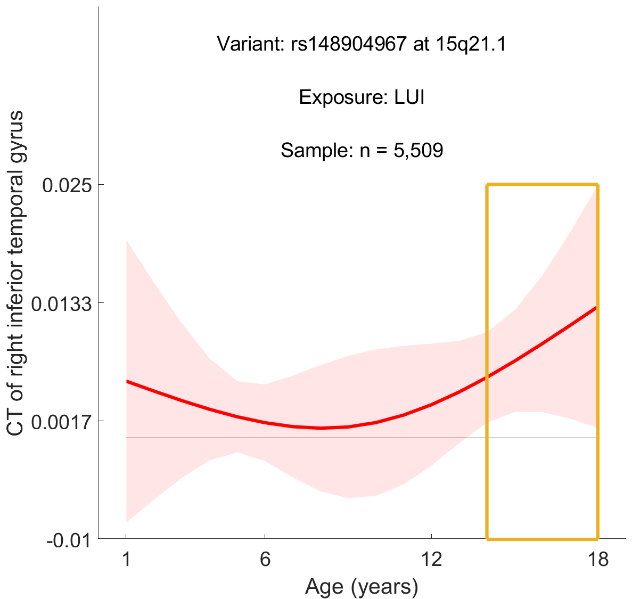

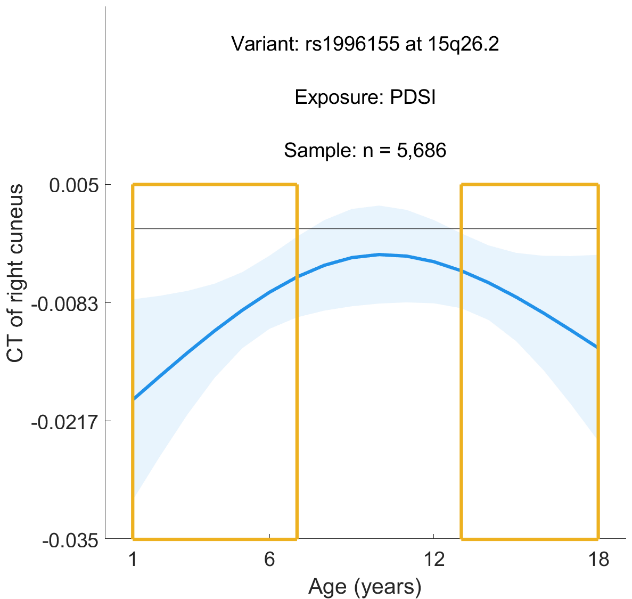

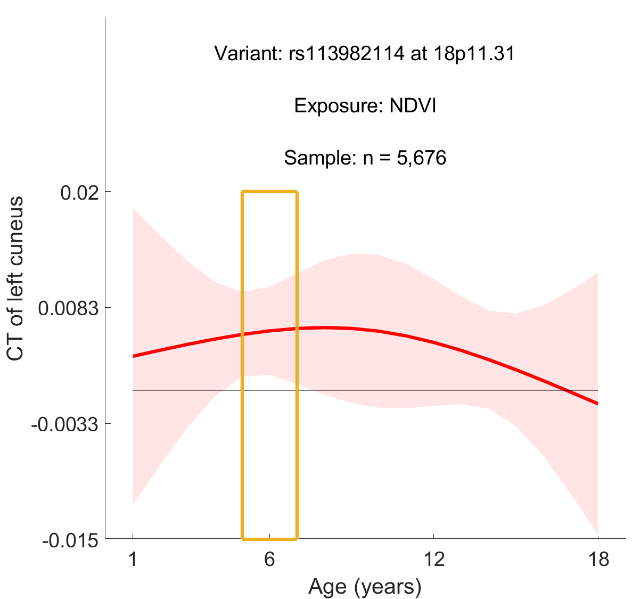

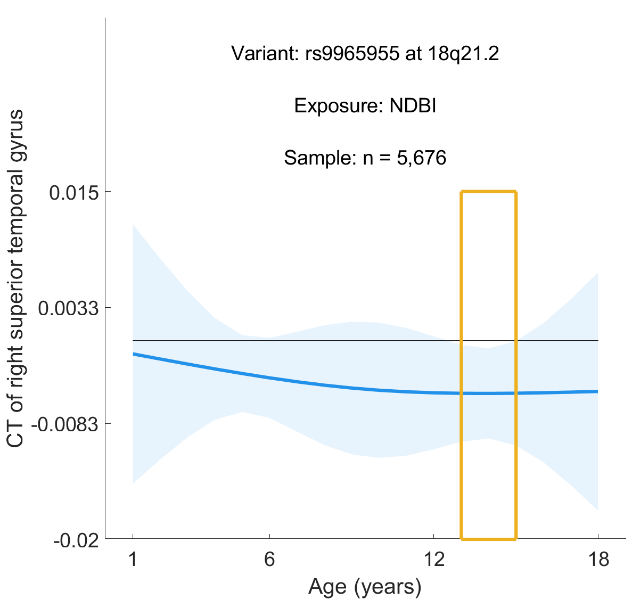

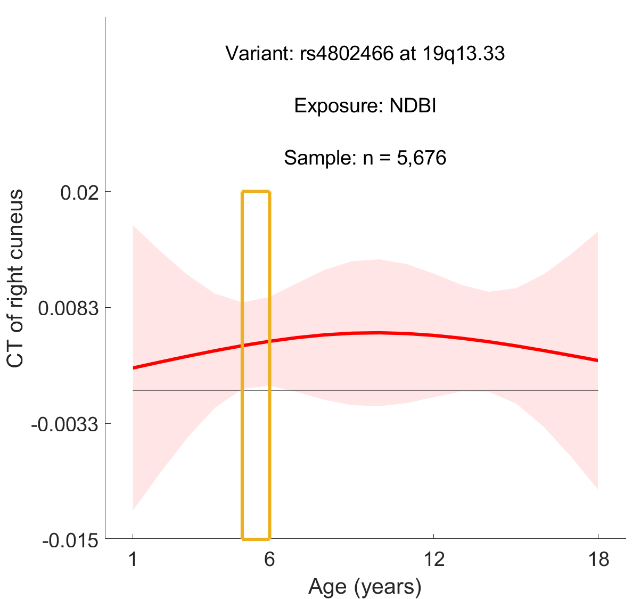
**

**Fig. S8. Sensitive periods for G × E interactions on CT-IDPs.** Each plot shows the sensitive period of one G × E interaction on a given CT-IDP. A sensitive period is defined as the period during which the estimated pointwise 95% CI does not include zero. Abbreviations: CI, confidence interval; CT, cortical thickness; IDP, imaging-derived phenotype; LUI, land use intensity; NDBI, normalized difference built-up index; NDVI, normalized difference vegetation index; NL, night-time light; PDSI, Palmer drought severity index.

**
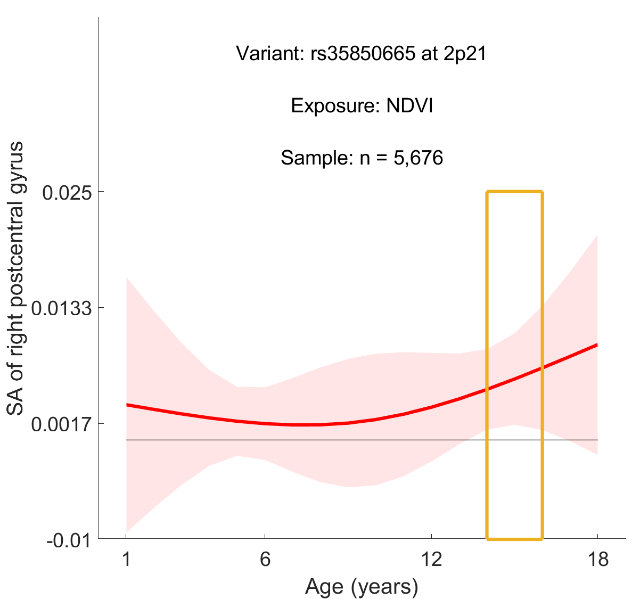

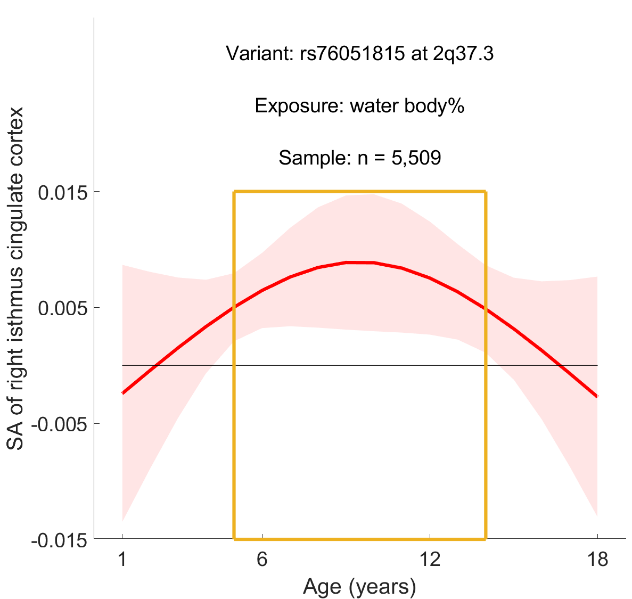

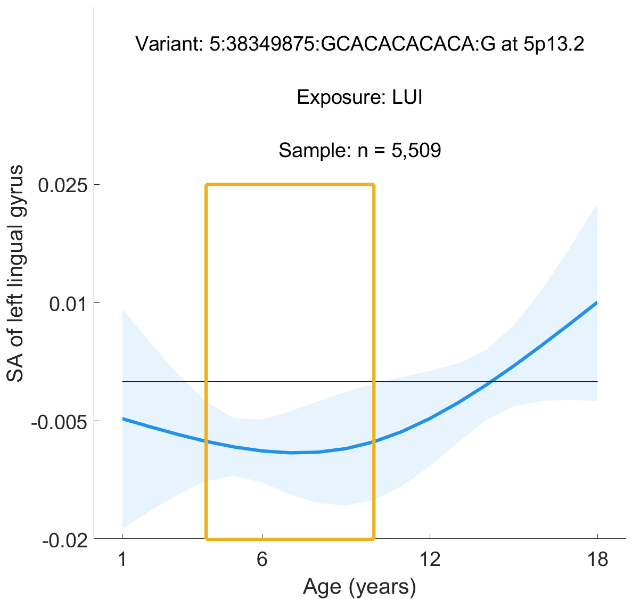

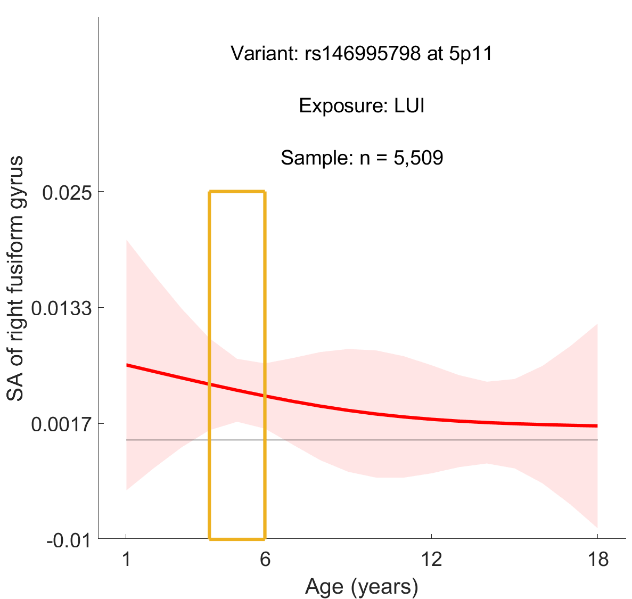

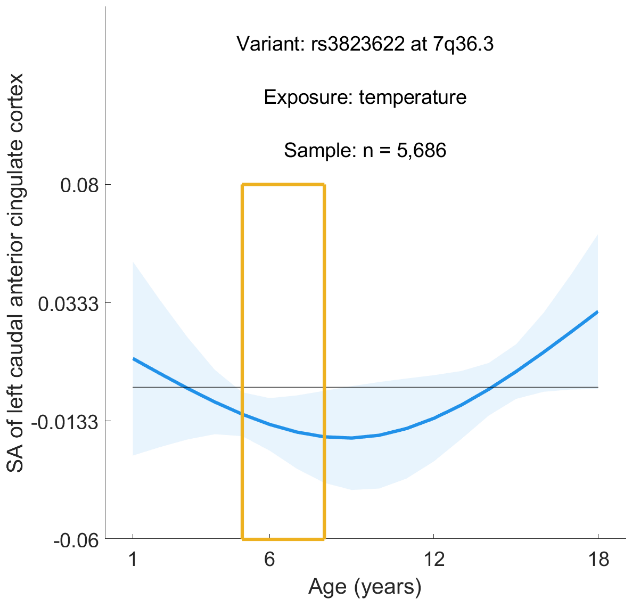

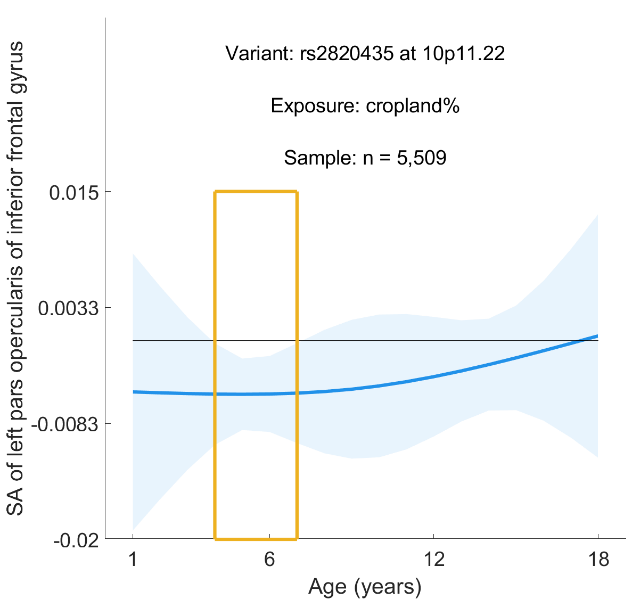

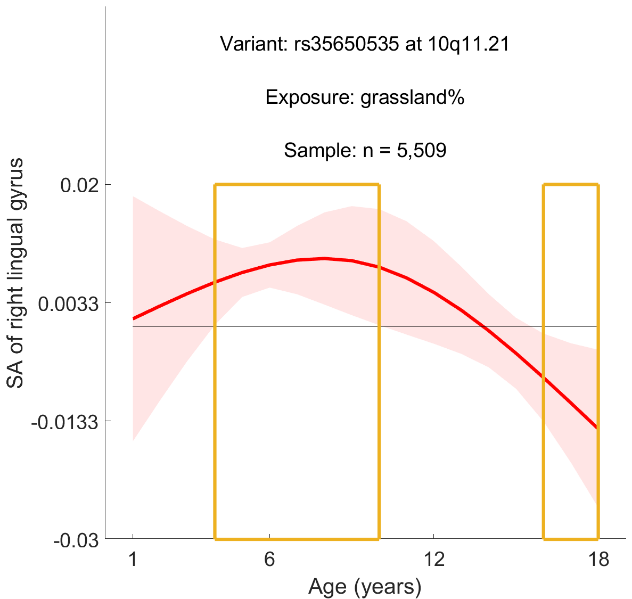

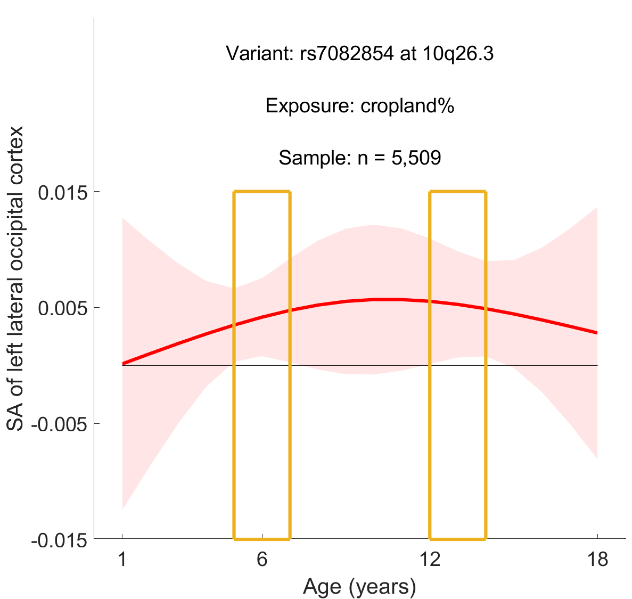

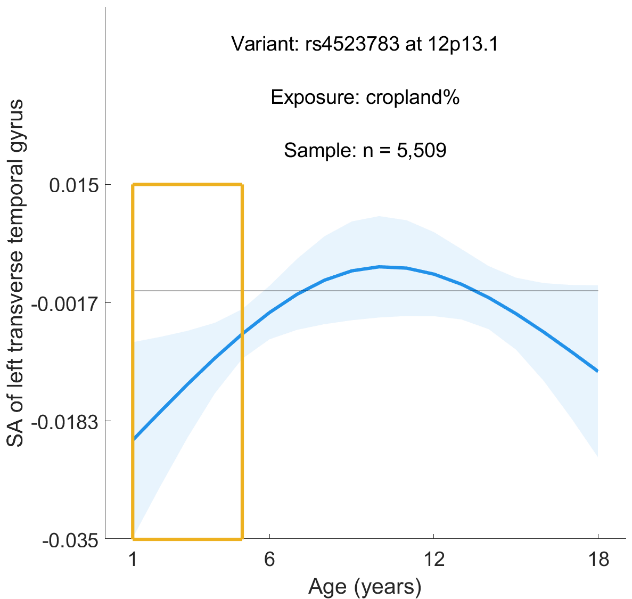

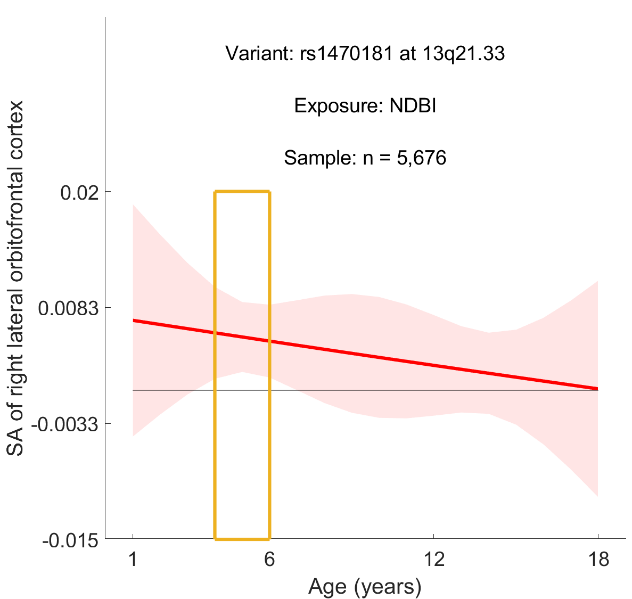

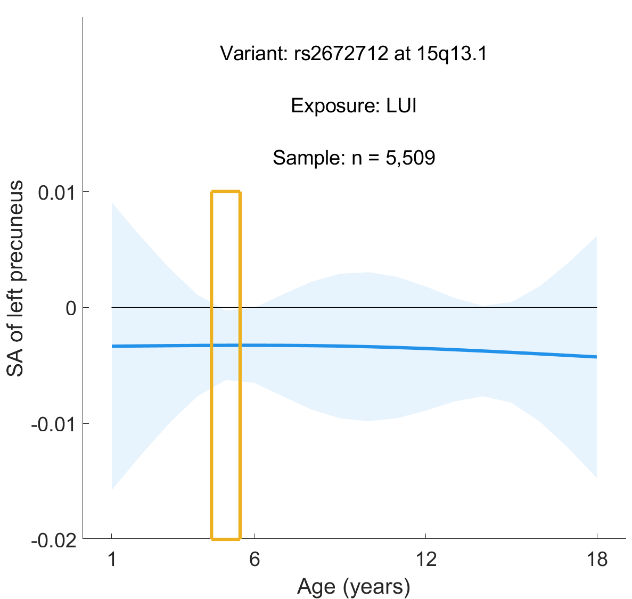

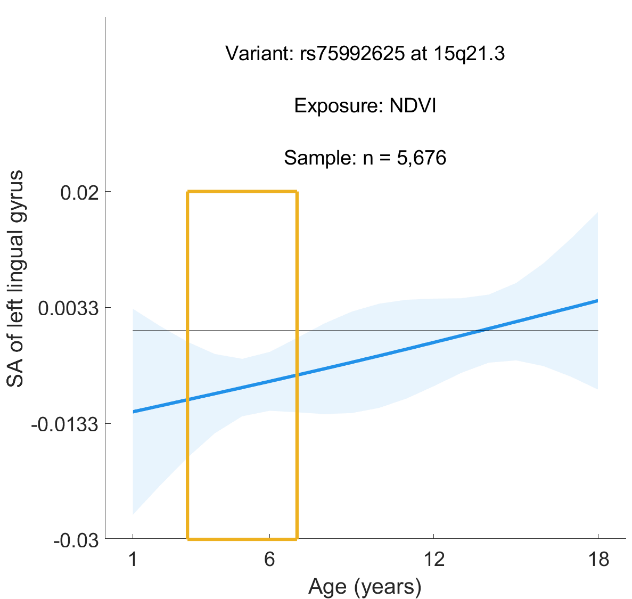

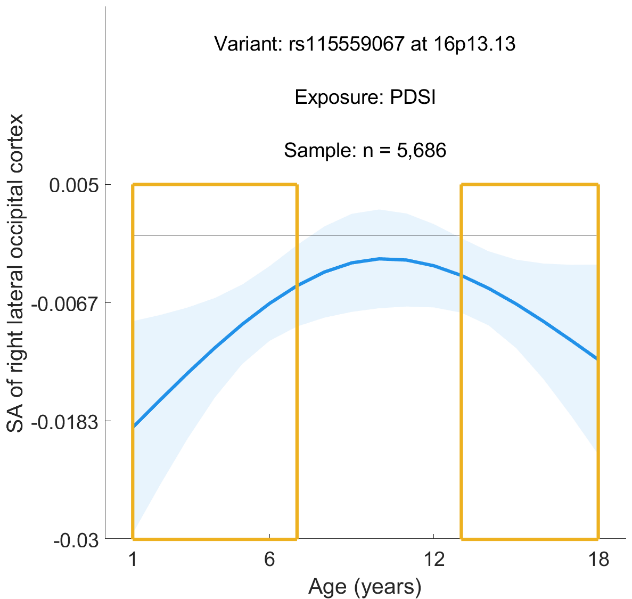

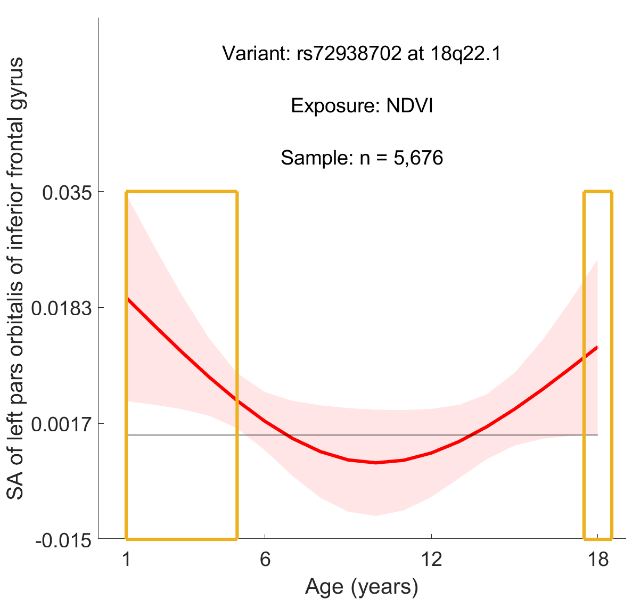
**

**Fig. S9. Sensitive periods for G × E interactions on SA-IDPs.** Each plot shows the sensitive period of one G × E interaction on a given SA-IDP. A sensitive period is defined as the period during which the estimated pointwise 95% CI does not include zero. Abbreviations: CI, confidence interval; IDP, imaging-derived phenotype; LUI, land use intensity; NDBI, normalized difference built-up index; NDVI, normalized difference vegetation index; PDSI, Palmer drought severity index; SA, surface area.

**
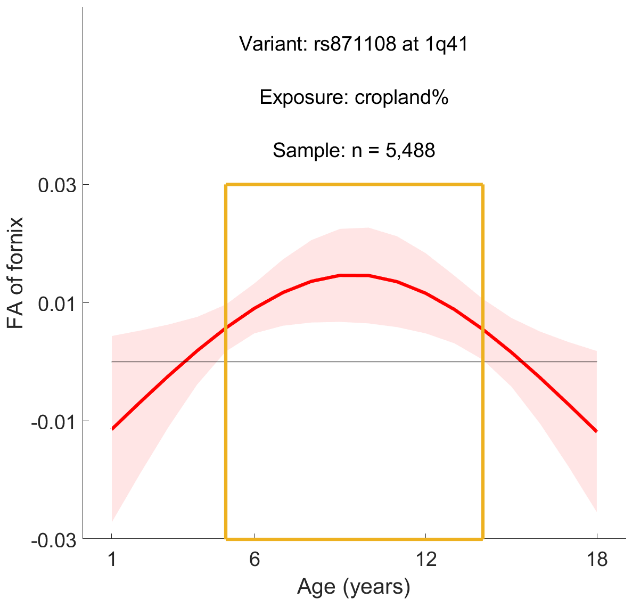

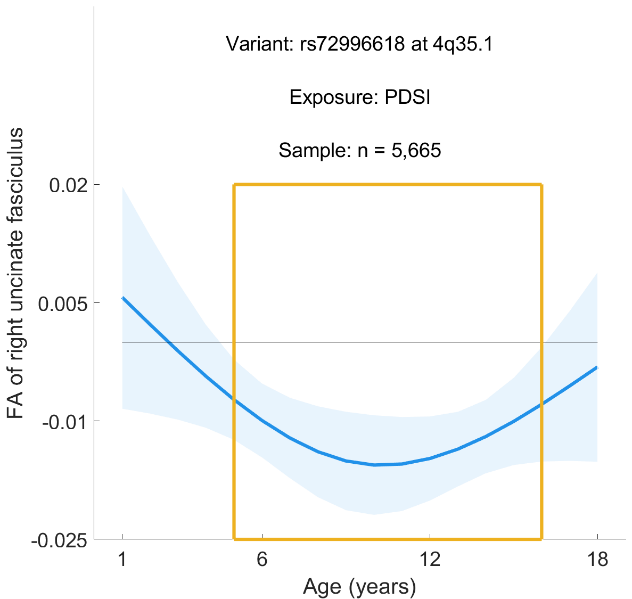

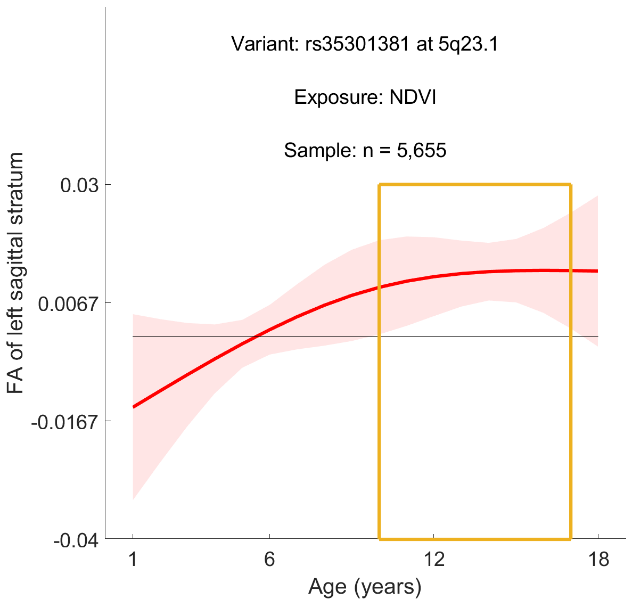

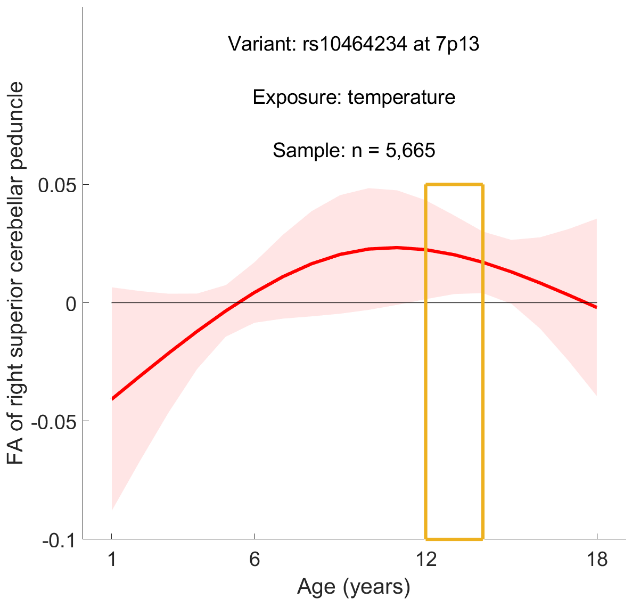

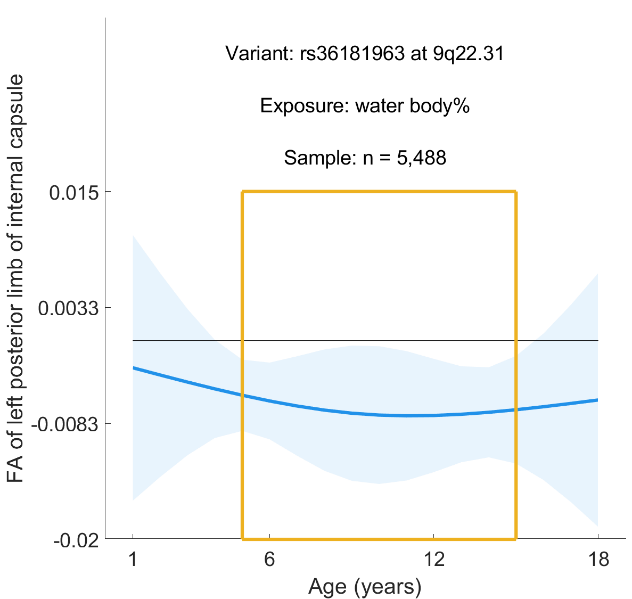

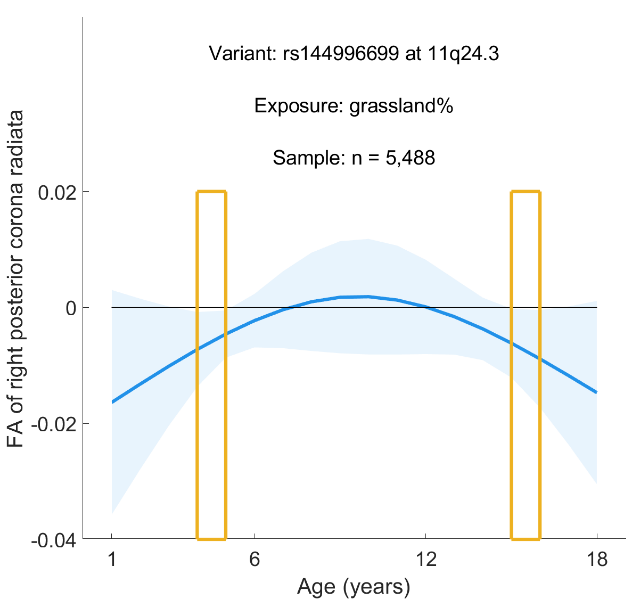

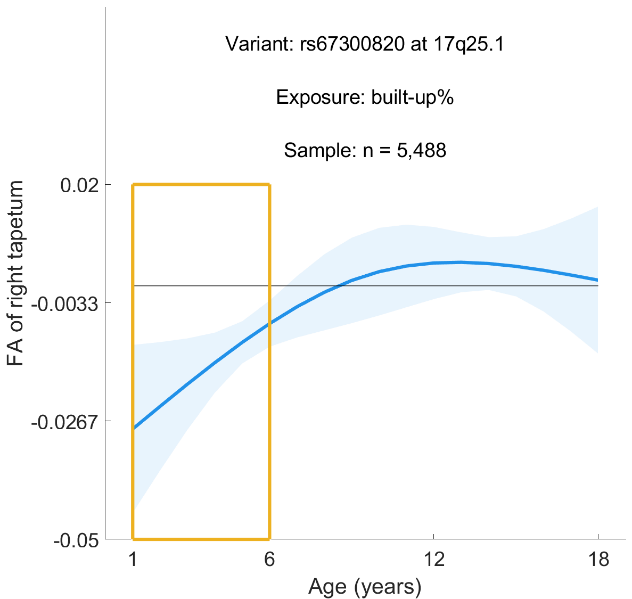
**

**Fig. S10. Sensitive periods for G × E interactions on FA-IDPs.** Each plot shows the sensitive period of one G × E interaction on a given FA-IDP. A sensitive period is defined as the period during which the estimated pointwise 95% CI does not include zero. Abbreviations: CI, confidence interval; FA, fractional anisotropy; IDP, imaging-derived phenotype; NDVI, normalized difference vegetation index; PDSI, Palmer drought severity index.

**
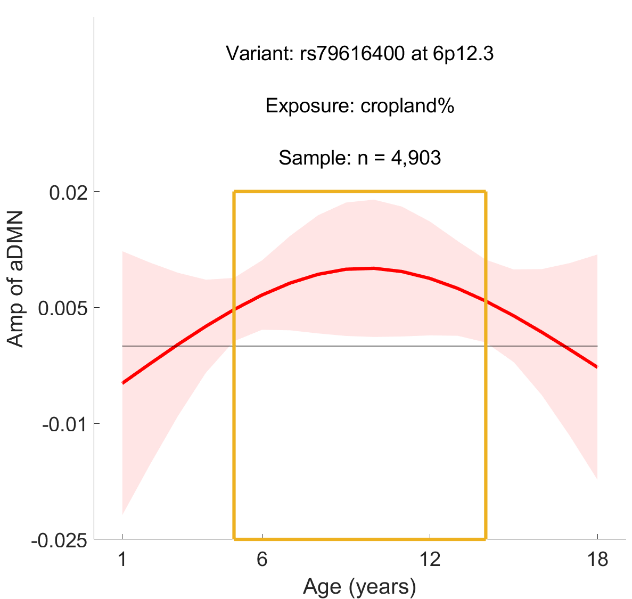

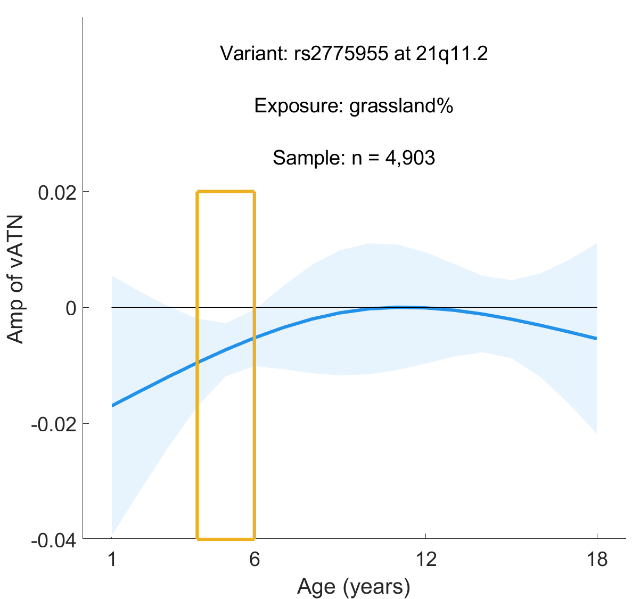
**

**Fig. S11. Sensitive periods for G × E interactions on Amp-IDPs.** Each plot shows the sensitive period of one G × E interaction on a given Amp-IDP. A sensitive period is defined as the period during which the estimated pointwise 95% CI does not include zero. Abbreviations: aDMN: anterior default-mode network; Amp, functional activity amplitude; CI, confidence interval; IDP, imaging-derived phenotype; vATN: ventral attentional network.

**
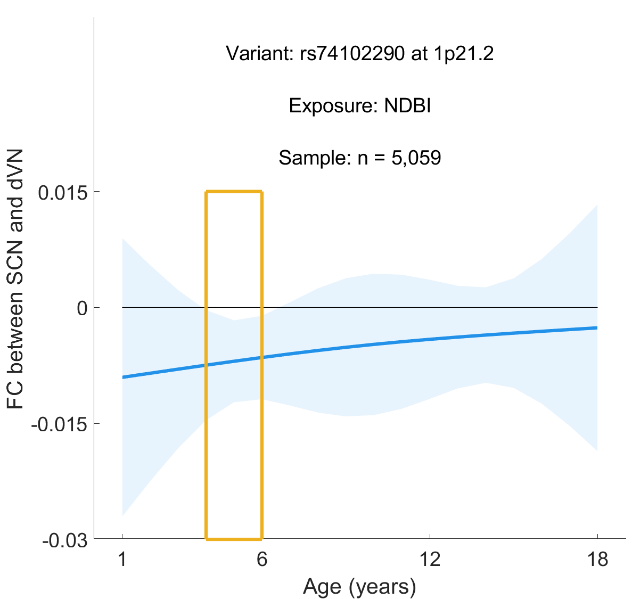

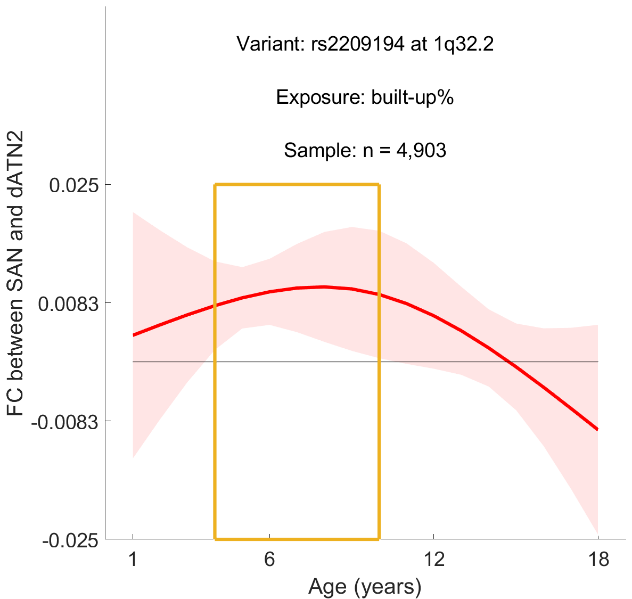

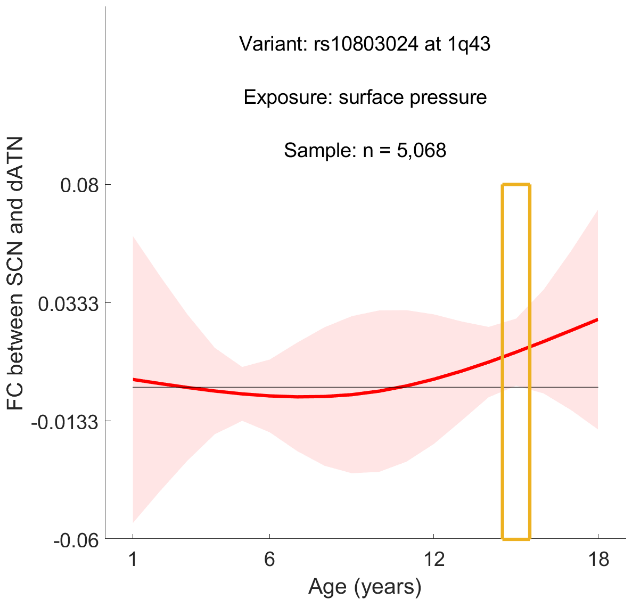

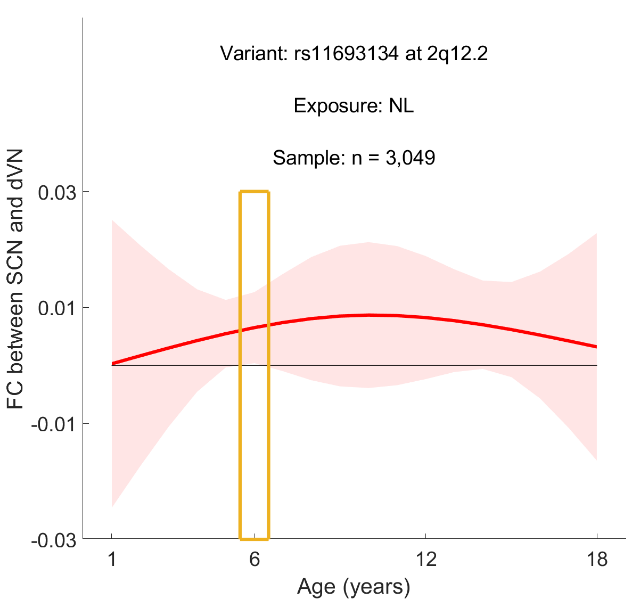

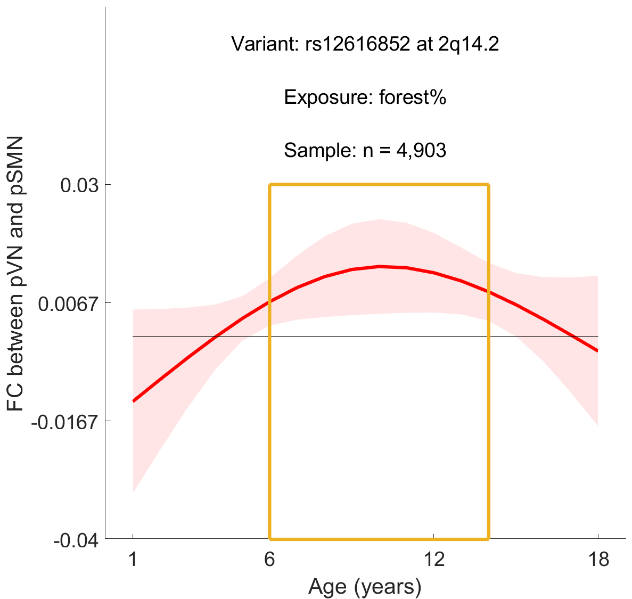

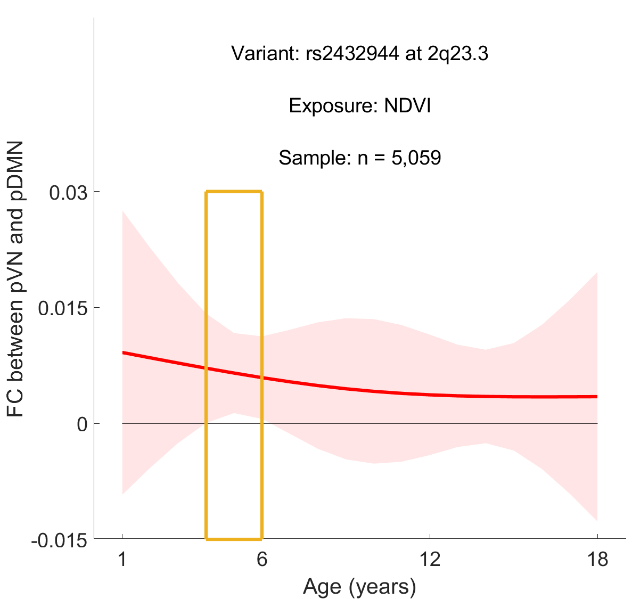

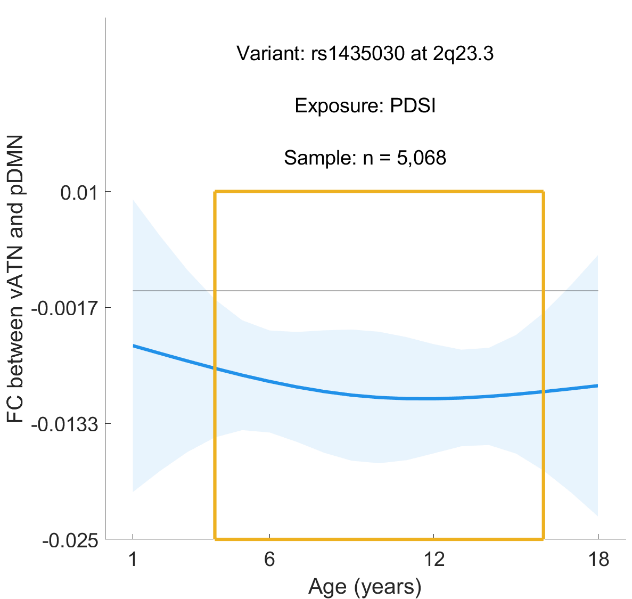

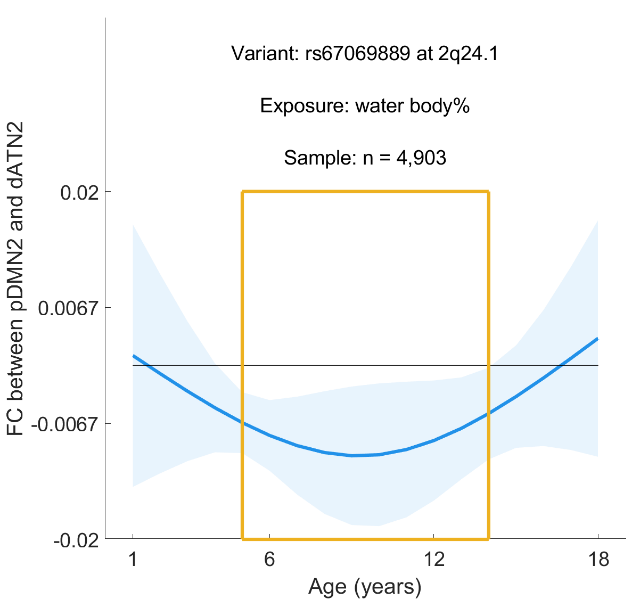

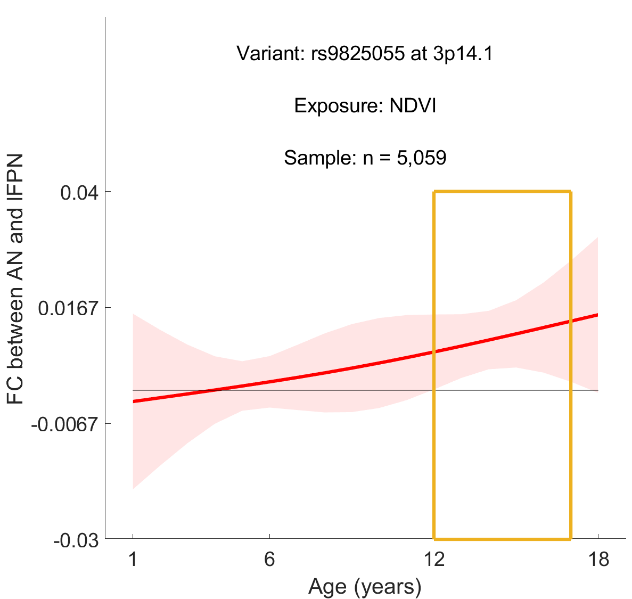

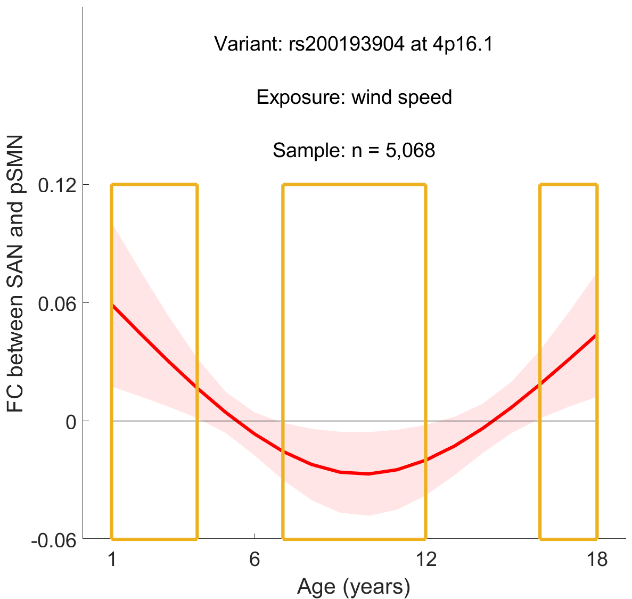

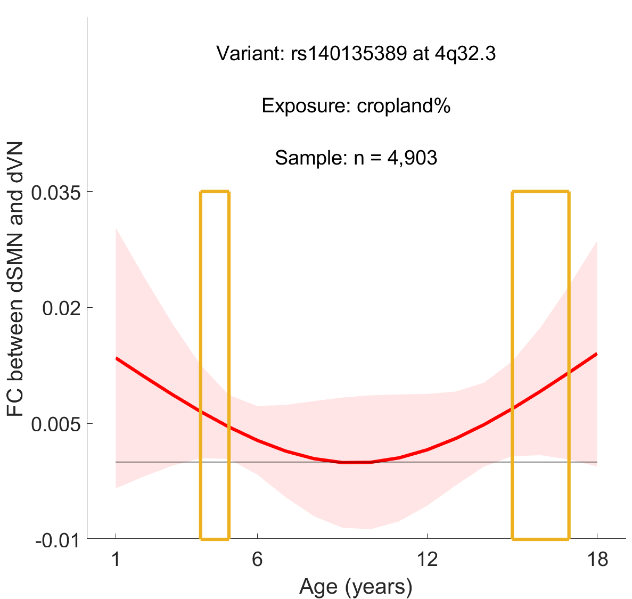

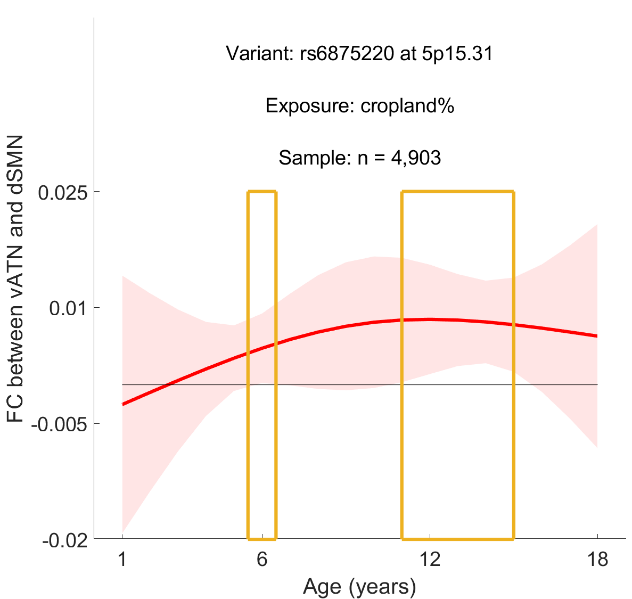

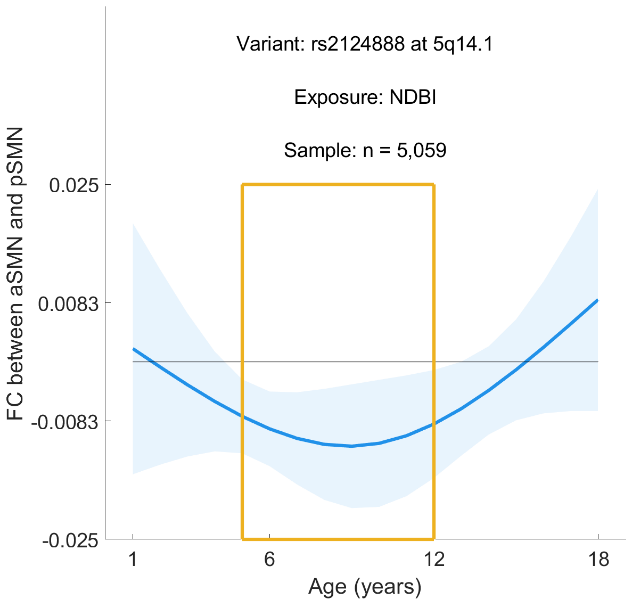

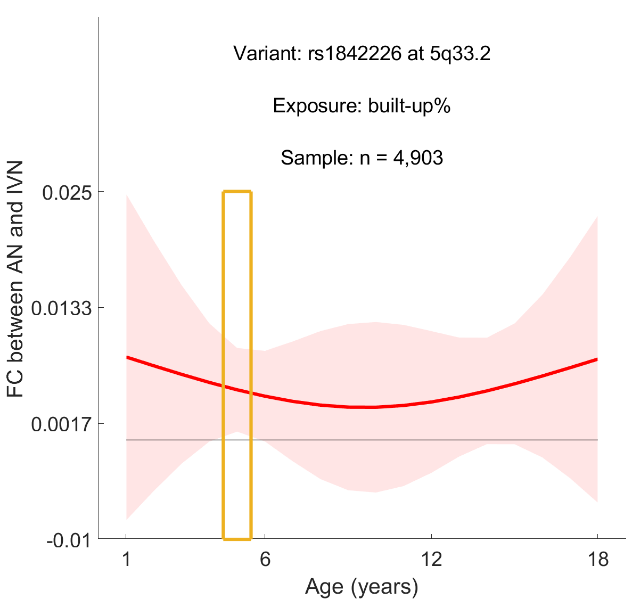

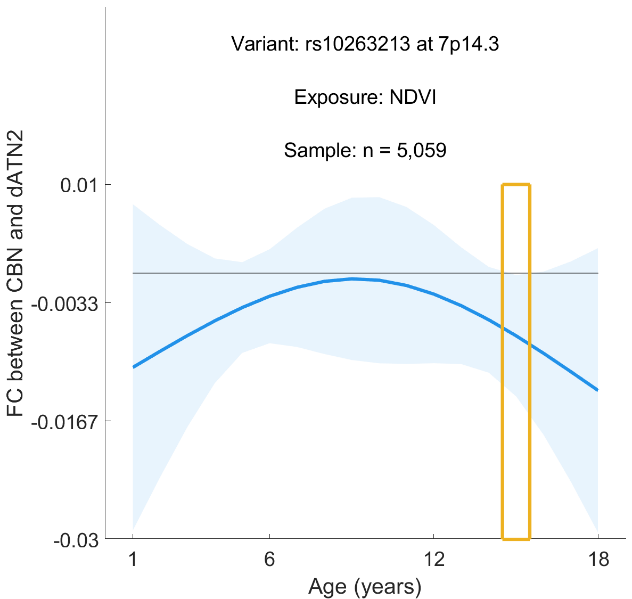

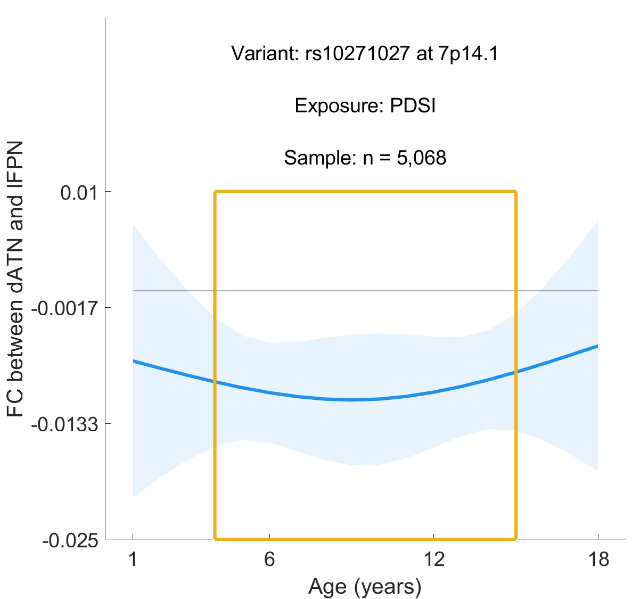

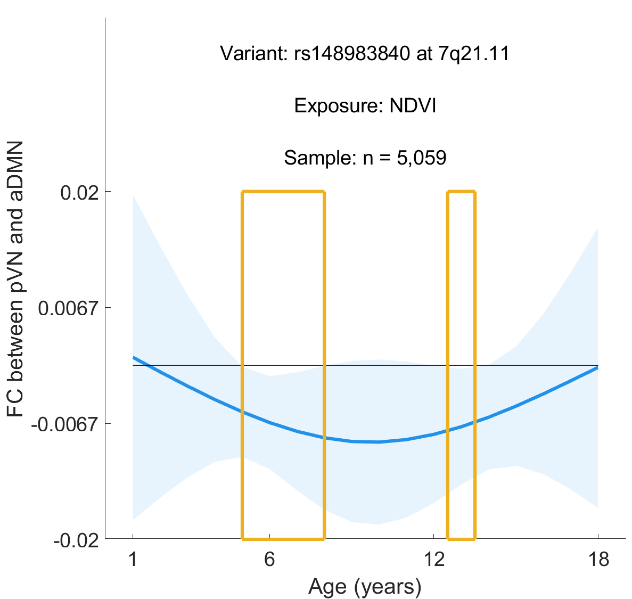

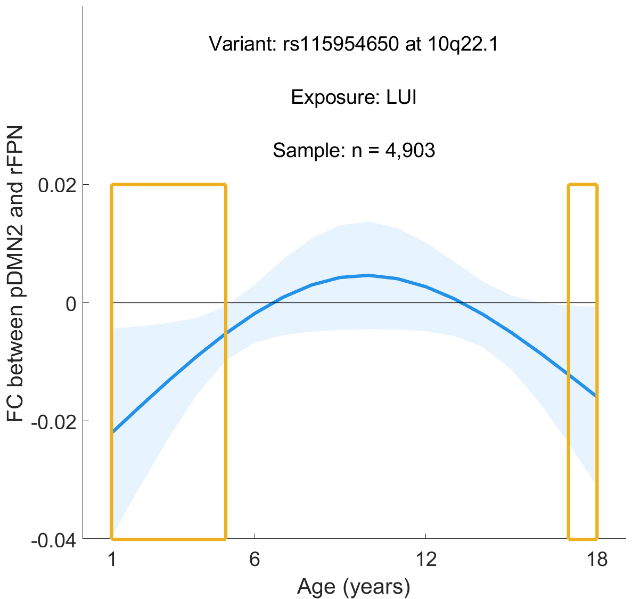

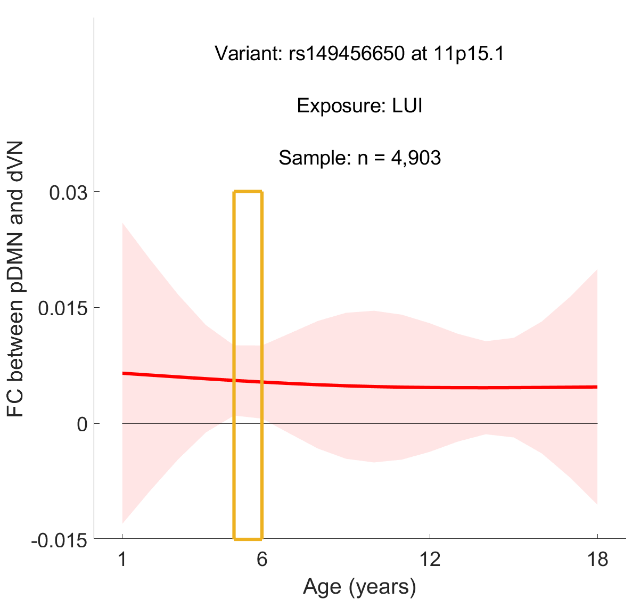

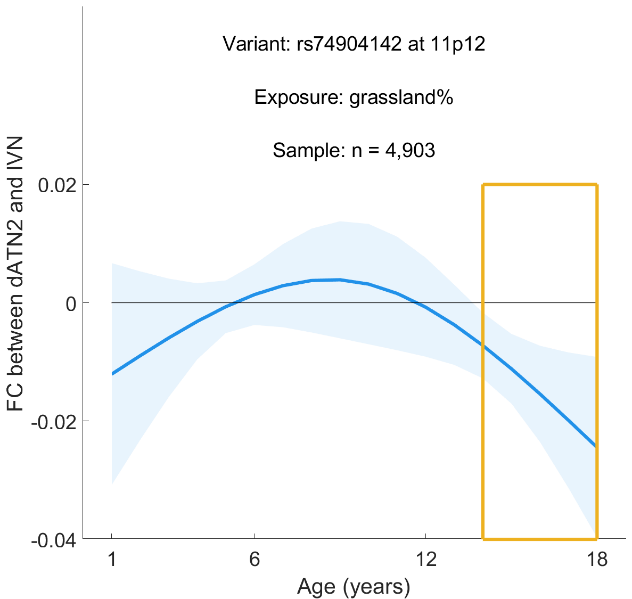

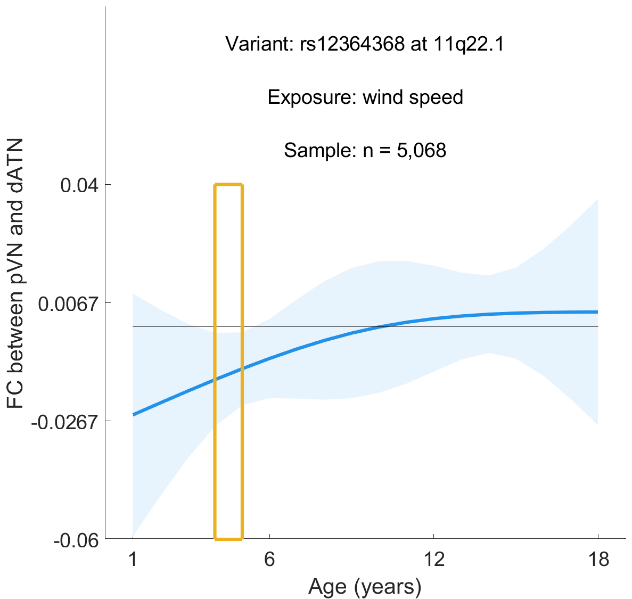

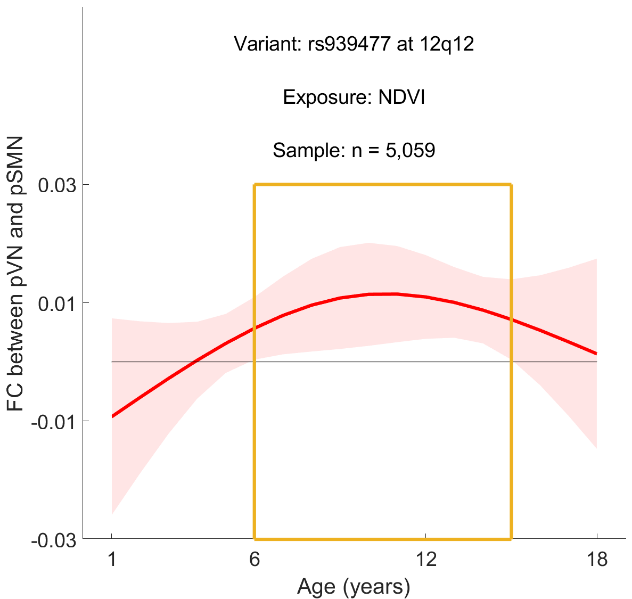

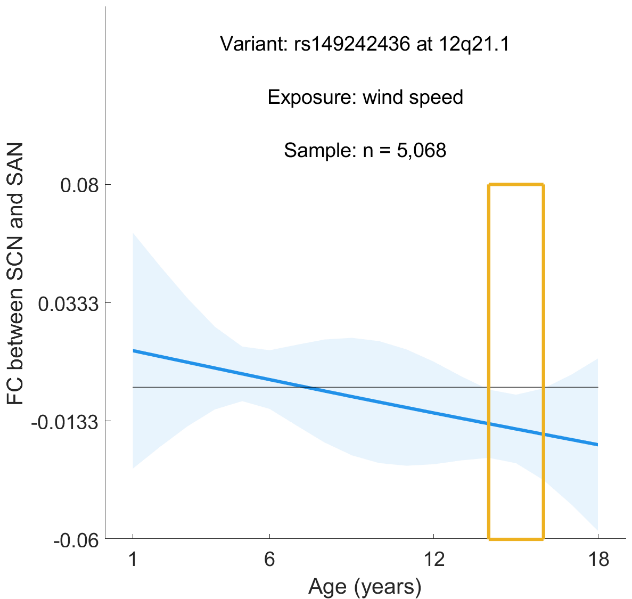

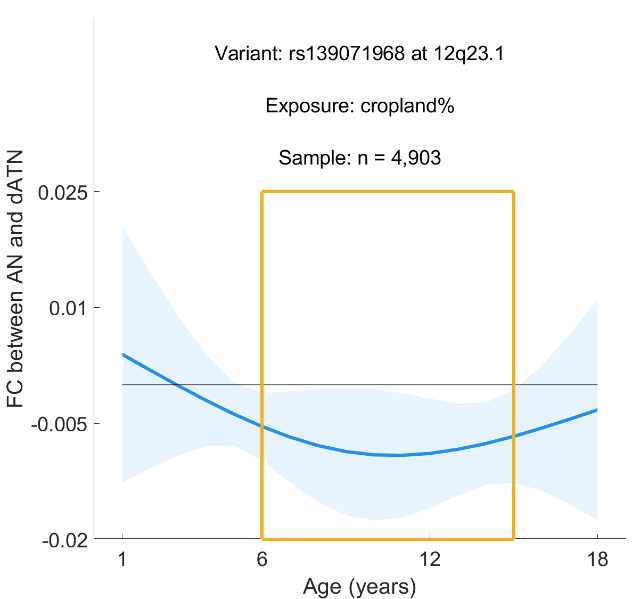

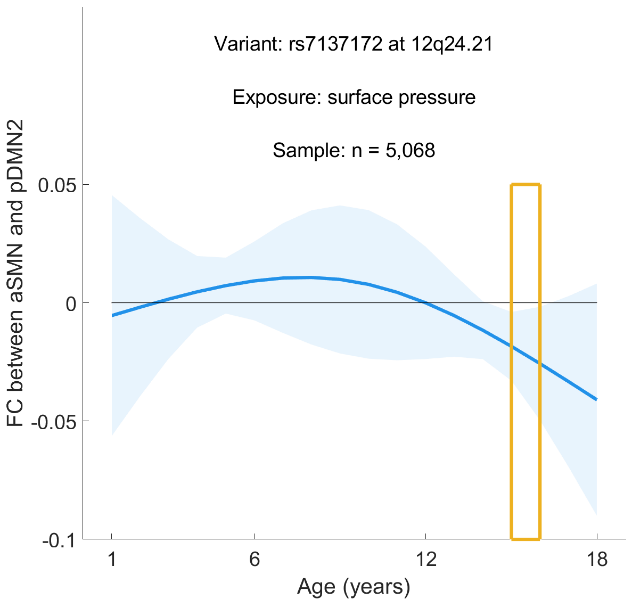

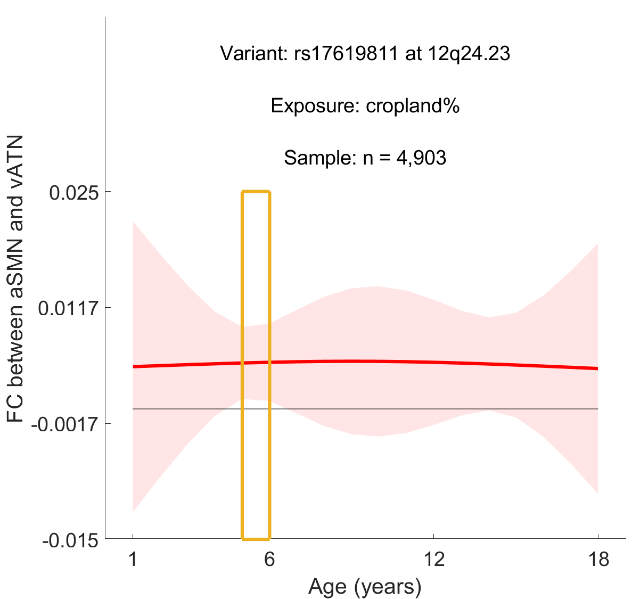
**

**Fig. S12. Sensitive periods for G × E interactions on FC-IDPs.** Each plot shows the sensitive period of one G × E interaction on a given FC-IDP. A sensitive period is defined as the period during which the estimated pointwise 95% CI does not include zero. Abbreviations: aDMN: anterior default-mode network; AN: auditory network; aSMN: anterior sensorimotor network; CBN: cerebellar network; CI, confidence interval; dATN: dorsal attentional network; dSMN: dorsal sensorimotor network; dVN: dorsal visual network; FC, functional connectivity; IDP, imaging-derived phenotype; lFPN: left frontal parietal network; LUI, land use intensity; lVN: lateral visual network; NDBI, normalized difference built-up index; NDVI, normalized difference vegetation index; NL, night-time light; pDMN: posterior default mode network; PDSI, Palmer drought severity index; pSMN: posterior sensorimotor network; pVN: primary visual network; rFPN: right frontal parietal network; SAN: salience network; SCN: subcortical network; vATN: ventral attentional network.

**Fig. S13. Sensitive periods for G × E interactions on ReHo-IDPs.** Each plot shows the sensitive period of one G × E interaction on a given ReHo-IDP. A sensitive period is defined as the period during which the estimated pointwise 95% CI does not include zero. Abbreviations: CI, confidence interval; IDP, imaging-derived phenotype; LUI, land use intensity; NDBI, normalized difference built-up index; NDVI, normalized difference vegetation index; NL, night-time light; PDSI, Palmer drought severity index; ReHo, regional homogeneity; TD, temperature difference.

**Fig. S14*.* The frequency distribution of sensitive periods of G × E interactions for** **each IDP category.** Each histogram shows the frequency distribution of sensitive periods of G × E interactions for each IDP category in the first 18 years of age. The seven categories of brain IDPs include GMV, CT, SA, FA, Amp, FC, and ReHo. Abbreviation: Amp, functional activity amplitude; CT, cortical thickness; FA, fractional anisotropy; FC, functional connectivity; GMV, gray matter volume; IDP, imaging-derived phenotype; ReHo, regional homogeneity; SA, surface area.

**Fig. S15*.* The frequency distribution of sensitive periods of G × E interactions for each time-varying exposure.** Each histogram shows the frequency distribution of sensitive periods of G × E interactions for each time-varying exposure in the first 18 years of age. The statistics only include the 16 time-varying exposures with complete data of the first 18 years in more than 3,000 participants. Abbreviations: LUI, land use intensity; NDBI, normalized difference built-up index; NDVI, normalized difference vegetation index; NL, night-time light; PDSI, Palmer drought severity index; TD, temperature difference.

**Fig. S16. Sensitive periods for G × E interactions involving NO_2_, PM_2.5_ and population count on brain IDPs.** Each plot shows the sensitive period of one G × E interaction on a given brain IDP. A sensitive period is defined as the period during which the estimated pointwise 95% CI does not include zero. Abbreviations: aDMN: anterior default-mode network; CI, confidence interval; CT, cortical thickness; FA, fractional anisotropy; FC, functional connectivity; GMV, gray matter volume; IDP, imaging-derived phenotype; NO_2_, nitrogen dioxide; PM, particular matter; pVN: primary visual network; ReHo, regional homogeneity; rFPN: right frontal parietal network; SA, surface area; SCN: subcortical network; vATN: ventral attentional network.

**Fig. S17. Raw data distribution of 41 environmental exposures.** Abbreviations: CTQ, childhood trauma questionnaire; LUI, land use intensity; NDBI, normalized difference built-up index; NDVI, normalized difference vegetation index; NL, night-time light; NO_2_, nitrogen dioxide; PDSI, Palmer drought severity index; PM, particular matter; TD, temperature difference.

**Fig. S18*.* The inter-scanner consistency of brain IDPs from the same subject before and after Combat harmonization.** Two CHIMGEN subjects travel to different centers and are scanned with 28 MRI scanners. In each subject, we calculate 64 CT-IDPs and 124 ReHo-IDPs based on the MRI data acquired from each scanner and computed Spearman correlation coefficients (dots) of the CT-IDPs (**a**) and ReHo-IDPs (**b**) derived from every two MRI scanners, respectively. After applying Combat harmonization to the brain IDPs, we re-calculate the inter-scanner correlations of these brain IDPs (64 CT-IDPs or 124 ReHo-IDPs). The Wilcoxon sum rank test is used to investigate the differences in correlation coefficients before (blue dots) and after (pink dots) harmonization. The inter-scanner correlations of CT-IDPs (**a**) and ReHo-IDPs (**b**) from both subjects (left and right panels) after harmonization are much higher than those before harmonization. Abbreviations: CHIMGEN, Chinese imaging genetics; CT, cortical thickness; IDP, imaging-derived phenotype; MRI, magnetic resonance imaging; ReHo, regional homogeneity.

**Fig. S19*.* Distribution of 127 GMV-IDPs across scanners before and after Combat harmonization.** The horizontal axis represents the 30 scanners. For each scanner, each point indicates the GMV of one of the 127 brain regions from a participant scanned by this equipment. The three lines in the box plots, from bottom to top, indicate the first quartile, median, and third quartile. Notably, MRI data of the 32 research sites are acquired using 30 scanners, as MRI data of center 15 are acquired at center 1 and MRI data of center 20 are acquired at center 2.

**Fig. S20*.* Distribution of 64 CT-IDPs across scanners before and after Combat harmonization.** Please refer to the specifications of these plots in **Fig. S19**.

**Fig. S21*.* Distribution of 64 SA-IDPs across scanners before and after Combat harmonization.** Please refer to the specifications of these plots in **Fig. S19**.

**Fig. S22*.* Distribution of 48 FA-IDPs across scanners before and after Combat harmonization.** Please refer to the specifications of these plots in **Fig. S19**.

**Fig. S23*.* Distribution of 18 Amp-IDPs across scanners before and after Combat harmonization.** Please refer to the specifications of these plots in **Fig. S19**.

**Fig. S24*.* Distribution of 64 153 FC-IDPs across scanners before and after Combat harmonization.** Please refer to the specifications of these plots in **Fig. S19**.

**Fig. S25*.* Distribution of 124 ReHo-IDPs across scanners before and after Combat harmonization.** Please refer to the specifications of these plots in **Fig. S19**.

**Fig. S26. Data availability of the 21 time-varying exposures in the first 18 years of age in the 5,851 participants.** Abbreviations: LUI, land use intensity; NDBI, normalized difference built-up index; NDVI, normalized difference vegetation index; NL, night-time light; NO_2_, nitrogen dioxide; PDSI, Palmer drought severity index; PM, particular matter; TD, temperature difference.

**Fig. S27. The spatial distribution of each RSN in the brain.** The 18 meaningful RSNs are identified by the 25-component group-ICA in 6,283 CHIMGEN participants. Abbreviations: aDMN: anterior default-mode network; AN: auditory network; aSMN: anterior sensorimotor network; CBN: cerebellar network; dSMN: dorsal sensorimotor network; dATN: dorsal attentional network; dVN: dorsal visual network; ICA, independent component analysis; lFPN: left frontal parietal network; lVN: lateral visual network; pDMN: posterior default mode network; pSMN: posterior sensorimotor network; pVN: primary visual network; rFPN: right frontal parietal network; RSN, resting-state network; SAN: salience network; SCN: subcortical network; vATN: ventral attentional network.
